# Genome-Wide Identification and Characterization of *Salvia miltiorrhiza* Laccases Reveal Potential Targets for Salvianolic Acid B Biosynthesis

**DOI:** 10.3389/fpls.2019.00435

**Published:** 2019-04-05

**Authors:** Qing Li, Jingxian Feng, Liang Chen, Zhichao Xu, Yingjie Zhu, Yun Wang, Ying Xiao, Junfeng Chen, Yangyun Zhou, Hexin Tan, Lei Zhang, Wansheng Chen

**Affiliations:** ^1^Department of Pharmacy, Changzheng Hospital, Second Military Medical University, Shanghai, China; ^2^Institute of Medicinal Plant Development, Chinese Academy of Medical Sciences, Peking Union Medical College, Beijing, China; ^3^Institute of Chinese Materia Medica, China Academy of Chinese Medical Sciences, Beijing, China; ^4^Department of Pharmaceutical Botany, School of Pharmacy, Second Military Medical University, Shanghai, China; ^5^State Key Laboratory of Subtropical Silviculture, Zhejiang A & F University, Hangzhou, China

**Keywords:** *Salvia miltiorrhiza*, laccase, genome-wide, bioinformatics, salvianolic acid B

## Abstract

Laccases are widely distributed in plant kingdom catalyzing the polymerization of lignin monolignols. Rosmarinic acid (RA) has a lignin monolignol-like structure and is converted into salvianolic acid B (SAB), which is a representatively effective hydrophilic compound of a well-known medicinal plant *Salvia miltiorrhiza* and also the final compound of phenolic acids metabolism pathway in the plant. But the roles of laccases in the biosynthesis of SAB are poorly understood. This work systematically characterizes *S. miltiorrhiza* laccase (SmLAC) gene family and identifies the SAB-specific candidates. Totally, 29 laccase candidates (SmLAC1-SmLAC29) are found to contain three signature Cu-oxidase domains. They present relatively low sequence identity and diverse intron–exon patterns. The phylogenetic clustering of laccases from *S. miltiorrhiza* and other ten plants indicates that the 29 SmLACs can be divided into seven groups, revealing potential distinct functions. Existence of diverse *cis* regulatory elements in the *SmLAC*s promoters suggests putative interactions with transcription factors. Seven *SmLAC*s are found to be potential targets of miR397. Putative glycosylation sites and phosphorylation sites are identified in SmLAC amino acid sequences. Moreover, the expression profile of *SmLAC*s in different organs and tissues deciphers that 5 *SmLAC*s (*SmLAC7/8/20/27/28*) are expressed preferentially in roots, adding the evidence that they may be involved in the phenylpropanoid metabolic pathway. Besides, silencing of *SmLAC7*, *SmLAC20* and *SmLAC28*, and overexpression of *SmLAC7* and *SmLAC20* in the hairy roots of *S. miltiorrhiza* result in diversification of SAB, signifying that *SmLAC7* and *SmLAC20* take roles in SAB biosynthesis. The results of this study lay a foundation for further elucidation of laccase functions in *S. miltiorrhiza*, and add to the knowledge for SAB biosynthesis in *S. miltiorrhiza*.

## Introduction

Laccase (*p*-diphenol: dioxygen oxidoreductase, EC.1.10.3.2), originally found in *Rhus vernicifera*, widely exists in fungi, bacteria, insects and plants (Yoshida, 1883; Wang et al., 2015). As a multicopper glycoprotein oxidase, laccase (LAC) mainly works in catalyzing one-electron oxidation of a wide range of substrates, coupled with the reduction of oxygen to water (Mot and Silaghi-Dumitrescu, 2012). LACs typically contain three conserved Cu-oxidase sites, named Type 1 (T1), Type 2 (T2), and binuclear Type 3 (T3) Cu sites respectively. When a substrate is bound and oxidized at T1, an electron is released and transferred to T2/T3 trinuclear copper cluster (TNC), consequently the free hydrogens are combined with molecule oxygens (O_2_) and reduced to water molecules (H_2_O) (Jones and Solomon, 2015). Due to the ability of oxidizing a variety of substrates, such as phenols, aromatic amines and metal ions, LACs have the potential to be used in industrial processes (Forootanfar and Faramarzi, 2015).

In recent years, great achievements have been made on the studies of LACs in lignin biosynthesis in plants (Liang et al., 2006a; Berthet et al., 2011; Cesarino et al., 2013; Zhao et al., 2013). In *Arabidopsis*, through T-DNA insertional mutagenesis, Berthet et al. (2011) found that the xylem was collapsed and the soluble constituents were detected in both laccase 4 (*AtLAC4*) and laccase 17 (*AtLAC17*) knockout mutants. By knocking down *AtLAC4* and *AtLAC17* along with *AtLAC11* (laccase 11), Zhao et al. (2013) observed serious physiological changes in the living plants, such as growth inhibition, narrowed stems and lack of lignified vascular bundles, indicating that *AtLAC11* may also be involved in the lignin polymerization. In addition to their functions in lignin biosynthesis (Cai et al., 2006; Liang et al., 2006a,b; Berthet et al., 2011; Zhao et al., 2013), LACs may perform other roles as some LACs are expressed in non-woody tissues and participate in oxidation of flavonoids (Pourcel et al., 2007; Turlapati et al., 2011).

**FIGURE 1:**
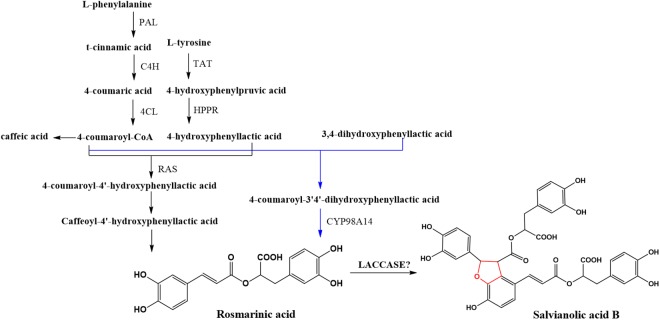
Scheme of the general biosynthetic pathway of SAB in *S. miltiorrhiza*.

*Salvia miltiorrhiza* (Dan-Shen) is one of the most commonly used medicinal plants in traditional Chinese medicine for treatment of cardiovascular and cerebrovascular diseases. Salvianolic acid B (SAB) is a representatively effective hydrophilic compound in *S. miltiorrhiza*. According to Chinese pharmacopeia (Chinese Pharmacopoeia Commission, 2015), it is also the quality control component of *S. miltiorrhiza*. Understanding the biosynthetic pathway of SAB will help improve the quality of *S. miltiorrhiza* by breeding improvement or quality control during the growth of *S. miltiorrhiza.* It will also benefit the metabolic engineering in *S. miltiorrhiza* such as increasing the abundance of SAB in the plant for extraction. Based on our studies of the biosynthetic pathway of salvianolic acids (Figure 1), a similar pathway to that of lignin and flavonoids, we suspected that LACs in the plant might be candidates in catalyzing rosmarinic acid (RA) to SAB (Di et al., 2013). In fact, five candidate LACs (SMil_00009266, SMil_00023004, SMil_00000484, SMil_00003461, and SMil_00018228) were claimed to participate in the salvianolic acids pathway (Xu Z. et al., 2016) in *S. miltiorrhiza*. However, SMil_00000484, SMil_00003461 and SMil_00018228 are non-members of LAC family. SMil_00000484 is a monocopper oxidase-like protein while SMil_00003461 and SMil_00018228 are both L-ascorbate oxidase homologs.

Under the umbrella of *S. miltiorrhiza* genome data, 80 LACs were annotated (Xu H. et al., 2016; Xu Z. et al., 2016). However, confirmation is required in urgent since not only could the annotation of LACs be easily mixed with other multi-copper oxidases and peroxidases, but the functions of LACs also vary in plants. Since there is no comprehensive analysis of LAC multigene family in *S. miltiorrhiza*, the aim of this work is to characterize *S. miltiorrhiza* laccases (SmLACs), with the long-term goal to identify *bona fide* LACs involved in SAB biosynthesis. For this purpose, we characterized the annotated LACs in *S. miltiorrhiza* through a genome-wide comprehensive analysis of the gene family including analysis of the gene structures, protein domains as well as putative promoter *cis* regulatory elements. A phylogenetic tree was constructed using the neighbor-joining method. In addition, the expression patterns of *SmLAC* genes were evaluated and confirmed by quantitative real-time PCR. Silencing and overexpression of the candidate *SmLACs* in the hairy roots of *S. miltiorrhiza* were carried out to detect the variation of SAB. To sum up, 29 LAC candidates were identified in *S. miltiorrhiza* genome. All of them have conserved copper-binding domains but are different in gene structures, indicating similar genetic origin but divergent biological functions. The potential regulation mechanism of *SmLAC* genes by transcription factors, miRNAs and phosphorylation were discussed. Five *SmLAC*s (*SmLAC7/8/20/27/28*) are assumed to be involved in the SAB biosynthetic pathway. Besides, silencing of *SmLAC7*, *SmLAC20* and *SmLAC28*, and overexpression of *SmLAC7* and *SmLAC20* in the hairy roots of *S. miltiorrhiza* resulted in diversification of SAB accumulation.

## Materials and Methods

### Genome-Wide Characterization of Laccase Genes in *S. miltiorrhiza*

Based on the annotation of *S. miltiorrhiza* genome, all the peptide sequences contained Cu-oxidase domains were extracted with related coding sequences. The peptide sequences were then verified while blasted in NCBI^1^ and checked on the Conserved Domain Database in the same website. Sequences with three typical Cu-oxidase domains were classified as LAC candidates after exclusion of L-ascorbate oxidase homologs and monocopper oxidase-like proteins.

The various physical and chemical characteristics of all the candidate SmLAC proteins were analyzed using the ProtParam tool^2^. Putative signal peptide cleavage sites were predicted by SignalP 4.1 server^3^ (Petersen et al., 2011). WoLF PSORT server^4^ and TargetP 1.1 server^5^ were used to predict the subcellular localization of the mature SmLAC proteins, respectively. Potential glycosylation sites and phosphorylation sites were separately analyzed through online NetNGlyc 1.0 Server^6^, YinOYang 1.2 server^7^, and NetPhos 2.0 Sever^8^. Visualization of the intron-exon structure of *SmLAC* genes was conducted by Gene Structure Display Server (GSDS 2.0)^9^ based on each coding sequence and corresponding genomic sequence. Sequence similarity of SmLAC proteins was obtained with alignment in ClustalX 2^10^, and the conserved sites were checked manually for their corresponding amino acid residues, which were shaded by GeneDoc software (Nicholas et al., 1997). Secondary structure predictions of SmLACs were performed with Secondary Structure Prediction Method (SOPMA^11^) (Combet et al., 2000). All the performance was carried out with default settings.

### Pairwise Distances, Phylogenetic and MEME Motif Analyses

The amino acid sequence alignments of SmLACs were performed using ClustalW implemented in the MEGA 5.05 software with p-distance settings. Phylogenetic and molecular evolutionary genetics analyses were performed using the Neighbor-Joining (NJ) method (Saitou and Nei, 1987) with pairwise deletion option in MEGA 5.05 and 1000 bootstrap replicates. The bootstrap values above 50% were added to the tree branches generated from the original dataset. Conserved motifs in the complete amino acid sequences of SmLACs were identified using MEME (Multiple EM for Motif Elicitation^12^) (Bailey et al., 2006) with the maximum number of motifs setting at 10.

### Prediction of miR397 Target Laccase Genes and *in silico* Analysis of *SmLAC* Promoter Sequences

The transcript sequences of the candidate *SmLAC*s were uploaded to web-based psRNATarget server^13^ for identification of potential targets corresponding to the ptr-miR397a and ssl-miR397. Sequences with a cut-off score ≤ 5 were chosen as putative targets.

PlantCARE (Plant *cis*-Acting Regulatory Elements^14^) was appointed to investigate the promoter sequences of *S. miltiorrhiza* LACs for potential *cis*-acting regulatory elements. The identified elements were sort out based on their reported functions.

### Methyl Jasmonate Treatment

Methyl jasmonate (MeJA) was dissolved in ethanol and added into the culture medium of hairy root at the final concentration of 0.1mM for 0, 4, 8, 16, and 24 h, respectively. Three independent biological replicates for each group were performed.

### Transcript Abundance of Laccases in Different Organs and Tissues of *S. miltiorrhiza*

To get insight into the transcript abundance of *SmLAC* genes, the Illumina and PacBio RNA-Seq data provided by Xu et al. (2015) was utilized. The RNA-Seq expression profile data were generated using different organs of mature *S. miltiorrhiza* at blooming stage, including roots, stems, leaves and flowers. The periderm, phloem and xylem of roots were also included. At least three biological replicates for each kind of organ and tissue were used. Finally, the heat map of *SmLAC* gene expression patterns was constructed using the log2 transformed and normalized expression level data in Multi Experiment Viewer (MeV).

### Plant Materials

The plant of *S. miltiorrhiza* was grown at the medicinal botanical garden of Second Military Medical University in Shanghai, China. It was identified by Professor Hanming Zhang. The fresh leaves, stems and roots of the plant were treated with liquid nitrogen immediately after collection, and then stored at -80°C for subsequently use. At least three biological duplicate samples of each organ were collected.

The hairy roots were derived after the infection of 60-day-old *S. miltiorrhiza* leaves with *Agrobacterium tumefaciens* C58C1, and stocked in 1/2 MS solid medium at 25°C in the dark. They were harvested from the culture medium at the 60th day after been transferred into liquid medium, and as much as 0.2 g of harvested hairy roots were used for total RNA isolation. The rest hairy roots were dried at 40°C in an oven until constant dry weight was reached.

### Preparation of RNA and cDNA

Total RNA of *S. miltiorrhiza* was isolated from stored roots, stems and leaves respectively using the *TransZol* Up Plus RNA Kit (TransBionovo Co., Ltd., Beijing, China). The integrity and quality of the RNA were confirmed by 1% agarose gels stained with ethidium bromide, and the RNA concentration was determined by a Nanodrop 2000 spectrophotometer (Thermo, Waltham, MA, United States). One μg of RNA for each sample was used for reverse transcription following the *TransScript* First-Strand cDNA Synthesis SuperMix operating procedures (TransBionovo Co., Ltd., Beijing, China).

### Quantitative Real-Time PCR

Quantitative real-time PCR was performed on a TaKaRa TP800 PCR system (TaKaRa, Japan) using *TransStart* Top Green qPCR SuperMix Kit (TransBionovo Co., Ltd., Beijing, China) according to the manufacturer’s instructions with three technical replicates. Primer Express 3.0^15^ was used to design the gene-specific primers (see Supplementary Table 1) for each *SmLAC*. Specificity of each primer pair was verified by 2% agarose gels and dissociation curve analysis. The transcript abundance of each *SmLAC* gene was normalized to *SmACTIN* as control and compared with roots as reference using 2^-ΔΔCt^ method.

### Construction of Recombinant Vectors

The candidate *SmLAC* cDNAs were cloned from S. *miltiorrhiza* through *pEASY*-Blunt Zero Cloning Vector (TransBionovo Co., Ltd., Beijing, China) respectively according to the manufacturer’s instructions. Then the fragments of cloned *SmLAC*s without the termination codons were PCR amplified using Primer 1 F/R and Primer 2 F/R (see Supplementary Table 2) respectively. They were cleaved with the correspondent restriction enzymes, and cloned into pCambia-1300 or pPHB-flag vectors to yield the RNAi or overexpression vectors.

### Extraction and Analysis of Phenolic Acids

The dried hairy roots were ground into powder, and 1 mL methanol: water (70:30, v/v) per 10 milligrams of powder was added into each sample. The mixture was sonicated for 30 min, 3 times with an output amplitude 50%. The solution was centrifuged at 13000 rpm for 10 min in an Eppendorf centrifuge 5418R rotor, and the supernatant was diluted with the same solvent to 5 mL per milliliter. Then the extract solution was filtered through a 0.2 μm organic membrane. Liquid chromatography-MS/MS (LC-MS/MS) was carried out to analyze the metabolites content using a triple-quadrupole mass spectrometer (Agilent G6410A). The separation was performed on a 2.1 × 50 mm 2.5 μm C18 column (Waters). The mobile phase consisted of acetonitrile: water (60:40 v/v).

## Results

### Genome-Wide Identification of Laccases in *S. miltiorrhiza*

To identify LAC genes in *S. miltiorrhiza* at the genome level, the genome database and the genome annotation information of *S. miltiorrhiza* were downloaded from http://www.ndctcm.org/shujukujieshao/2015-04-23/27.html (Xu H. et al., 2016). According to the annotation information, 80, 80 and 79 genes models were identified containing Cu-oxidase, Cu-oxidase_2 and Cu-oxidase_3 domain respectively, which are the typical domains of LACs (see Supplementary Table 3).

After combining the Cu-oxidase domain contained genes, 101 sequences were obtained. They were then blasted in NCBI and checked on via the Conserved Domain Database in NCBI. The results showed that 32 genes were non-laccases, and 40 ones were partial laccases because of low quality sequencing or assembling, and only 29 were full-length LACs (see Supplementary Table 4). SMil_00000484, SMil_00003461, and SMil_00018228 are all Cu-oxidase domain contained genes, but they are not LACs as mentioned in previous studies (Xu Z. et al., 2016). SMil_00000484 is a monocopper oxidase-like protein while SMil_00003461 and SMil_00018228 are both L-ascorbate oxidase homologs.

The 29 full-length LACs of *S. miltiorrhiza* [named SmLAC1 to SmLAC29 randomly (see Supplementary Text 1)] were applied for bioinformatics analysis. Generally, SmLACs consist of 500–600 amino acids (aa) and most between 560 and 580 aa. The molecular masses of the 29 SmLAC proteins range from circa 57.15 kDa (SmLAC15) to 67.70 kDa (SmLAC16) and the predicted theoretical isoelectric points (*p*I) range from 5.83 (SmLAC12) to 9.47 (SmLAC17). The signal peptide prediction showed that 23 of the SmLACs had a 20–30 aa length signal peptide at the N-terminus, indicating that most SmLACs are probably secreted proteins. This agrees with McCaig’s finding that most plant LACs have a cleavable N-terminal signal peptide targeting themselves to the secretory pathway (McCaig et al., 2005). The subcellular prediction showed that most of SmLAC proteins are localized in the secretory pathway and a few of them in mitochondria (SmLAC17 and SmLAC23) or nucleus (SmLAC5), indicating that most SmLACs are extracellular proteins. In addition, variable *N*- or *O-glycosylation* sites and phosphorylation sites were predicted to present in all SmLAC proteins, indicating potential post-translational modifications (Table 1).

**Table 1:** Physical, chemical characterization and the prediction of signal peptide and protein location of 29 SmLACs.

Designate name	Sequence ID	Amino acid length (aa)	MW (kD)	*p*I	Signal peptide length (aa)	Cleavage site	Protein location	Potential glycosylation sites		Potential phosphorylation sites		
								*N*-glyc	*O*-glyc	Serine	Threonine	Tyrosine
SmLAC1	SMil_00001393	573	64.64	6.79	24	VHA-LV	Secretory	4	8	18	16	6
SmLAC2	SMil_00001395	575	64.80	6.79	24	VDA-LV	Secretory	5	12	20	12	9
SmLAC3	SMil_00006361	564	62.37	9.45	23	AMA-KL	Secretory	10	10	13	17	9
SmLAC4	SMil_00008399	573	63.12	9.21	28	ANA-IT	Secretory	12	8	13	23	5
SmLAC5	SMil_00008533	588	64.86	8.91	–	–	Nuclear	9	15	17	18	6
SmLAC6	SMil_00009265	523	59.04	8.38	22	VHG-KI	Secretory	7	8	16	12	8
SmLAC7	SMil_00009266	562	63.21	6.21	22	VHG-KI	Secretory	9	8	19	16	8
SmLAC8	SMil_00009822	557	60.73	9.29	21	VDG-AV	Secretory	7	12	19	20	3
SmLAC9	SMil_00011367	579	66.22	8.46	–	–	Extracellular	5	5	23	17	8
SmLAC10	SMil_00012308	573	63.31	8.54	24	VHG-IK	Secretory	8	8	19	17	6
SmLAC11	SMil_00013111	571	63.98	6.55	21	VHA-VV	Secretory	5	11	20	15	10
SmLAC12	SMil_00017786	562	63.39	5.83	26	VNA-SI	Secretory	4	8	14	22	10
SmLAC13	SMil_00019237	548	62.46	8.62	–	–	Extracellular	5	6	20	15	7
SmLAC14	SMil_00020653	562	62.95	8.84	23	VKA-SD	Secretory	6	6	19	13	8
SmLAC15	SMil_00020657	528	57.15	9.21	25	VDS-RI	Secretory	5	19	19	20	3
SmLAC16	SMil_00021274	595	67.70	8.22	–	–	Extracellular	3	9	24	16	7
SmLAC17	SMil_00021810	593	65.11	9.47	–	–	Mitochondrial	9	9	24	16	8
SmLAC18	SMil_00022417	543	60.48	9.27	23	ANA-ET	Secretory	8	16	14	21	8
SmLAC19	SMil_00023003	565	63.50	7.29	26	SYA-MT	Secretory	11	8	13	21	13
SmLAC20	SMil_00023004	568	63.37	6.46	26	IHA-KT	Secretory	11	13	23	21	8
SmLAC21	SMil_00023210	551	61.77	6.42	30	VHA-VV	Secretory	5	12	14	17	7
SmLAC22	SMil_00023712	577	63.50	9.17	30	ASC-IT	Secretory	15	11	16	23	9
SmLAC23	SMil_00023714	556	63.33	8.05	-	-	Mitochondrial	4	5	22	16	11
SmLAC24	SMil_00023969	542	59.61	9.05	25	AEA-IT	Secretory	11	7	12	21	6
SmLAC25	SMil_00024180	570	62.14	5.99	24	ASA-RI	Secretory	12	12	17	20	7
SmLAC26	SMil_00025257	565	63.23	6.62	26	SHA-AT	Secretory	11	12	19	18	7
SmLAC27	SMil_00026282	559	61.10	8.87	22	VES-RV	Secretory	11	14	16	28	4
SmLAC28	SMil_00028093	575	65.09	8.02	28	VHA-LV	Secretory	2	10	20	20	7
SmLAC29	SMil_00028534	566	62.21	8.54	24	ASA-AI	Secretory	11	13	20	28	5

### Gene Structure Analysis of *S. miltiorrhiza* Laccase Family

**FIGURE 2:**
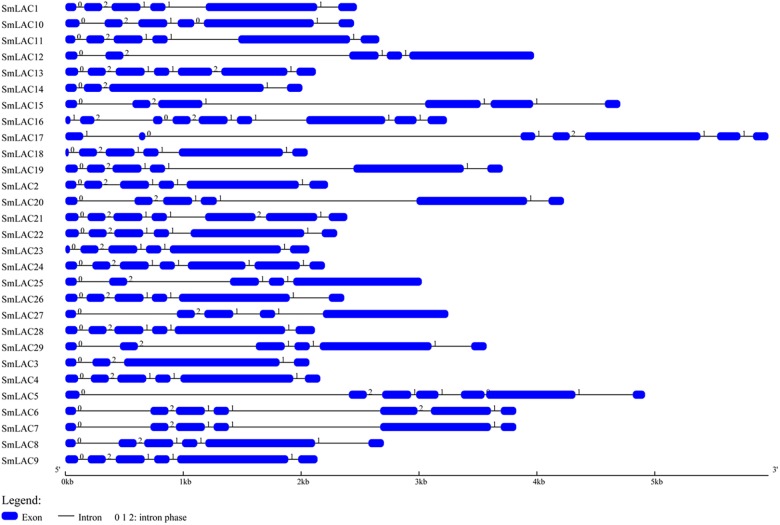
The structural features of each *SmLAC* gene. The blue round-cornered boxes represent exons and the black lines connecting two exons represent introns. The numbers above the line are the intron phase.

The gene structure of *SmLAC*s was investigated by using Gene Structure Display sever (Figure 2). The number of exons in the 29 *SmLAC*s varied from 4 to 9, indicating a diverse intron-exon pattern within *SmLAC* genes. In general, there were 17 genes containing 5 introns, 6 genes containing 6 introns, 3 genes containing 4 introns and 2 genes containing 3 introns. Gene *SmLAC16* contained 8 introns. It is noteworthy that all the genes consisted of an intron phase 0 at the initiating terminal (except *SmLAC16* and *SmLAC17*) and an intron phase 1 at the C-terminal, which indicates that they are relatively conserved.

The secondary structures of SmLACs were predicted on SOPMA. The results showed that random coil element was the main unit in SmLACs, followed by extended strand and α-helix (see Supplementary Table 5). The proportion of the α-helix structure in the 29 SmLACs ranges from 12.74% (SmLAC26) to 21.43% (SmLAC5), and the β-turn structure ratio from 8.66% (SmLAC18) to 12.63% (SmLAC25). The extended strand and random coil are from 26.52% (SmLAC15) to 32.23% (SmLAC18), and 39.27% (SmLAC10) to 46.90% (SmLAC26) respectively.

### Protein Sequence Similarity Analysis

**FIGURE 3:**
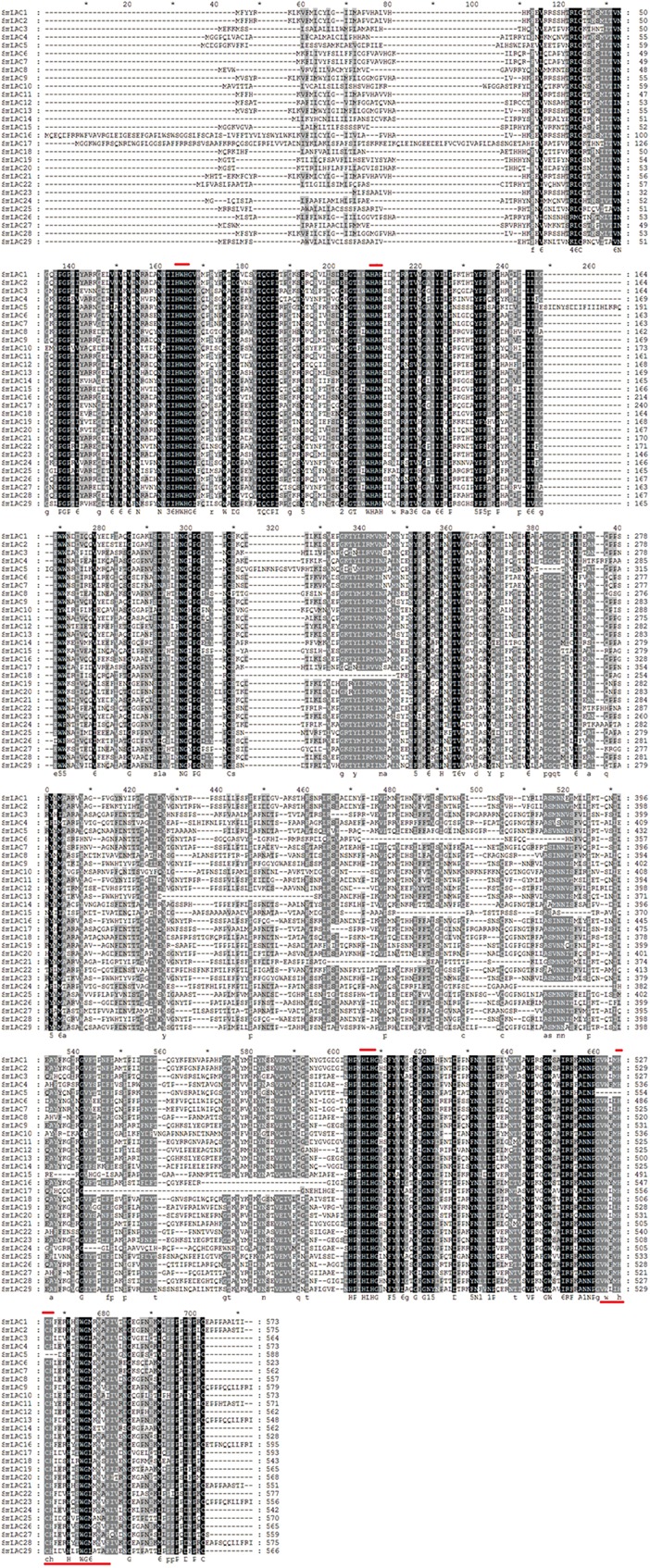
Alignment of amino acid sequences of SmLACs. Dark boxes refer to amino acid identity or similarity between the 29 sequences. Bars placed at the top of the sequences correspond to the four highly conserved ligands for copper whereas the bar placed on the bottom corresponds to the blue copper oxidase signature. Amino acid residue number is indicated on the right side of the figure for each protein.

Protein sequence similarity of the 29 SmLACs was first carried out through sequence comparisons. According to the result of sequence alignment (Figure 3), the highly conserved residues with 4 coordinated copper atoms were found, except SmLAC5 which lacks the T1 copper binding site (H-C-H) near the C-terminus. It was believed that the axial ligand near the T1 copper binding site (H-C-H-X3-H-X3-G-[LMI(F)]) proximal to the C-terminus partially influenced LAC redox potential (Turlapati et al., 2011). Functions of SmLAC5 might be different and need further examination.

Pairwise sequence similarities among the predicted 29 peptide sequences of SmLACs ranged from a low of 39.7% (SmLAC10 vs. SmLAC16) to a high of 98.2% (SmLAC9 vs. SmLAC13) (see Supplementary Table 6). For most ones, the identity percentage varied from 40 to 70%. SmLAC9 and SmLAC13 are examples of closely related proteins sharing amino acid identity greater than 98% that may represent within-species alleles.

### Phylogenetic Analysis and Conserved Motifs Identification

**FIGURE 4:**
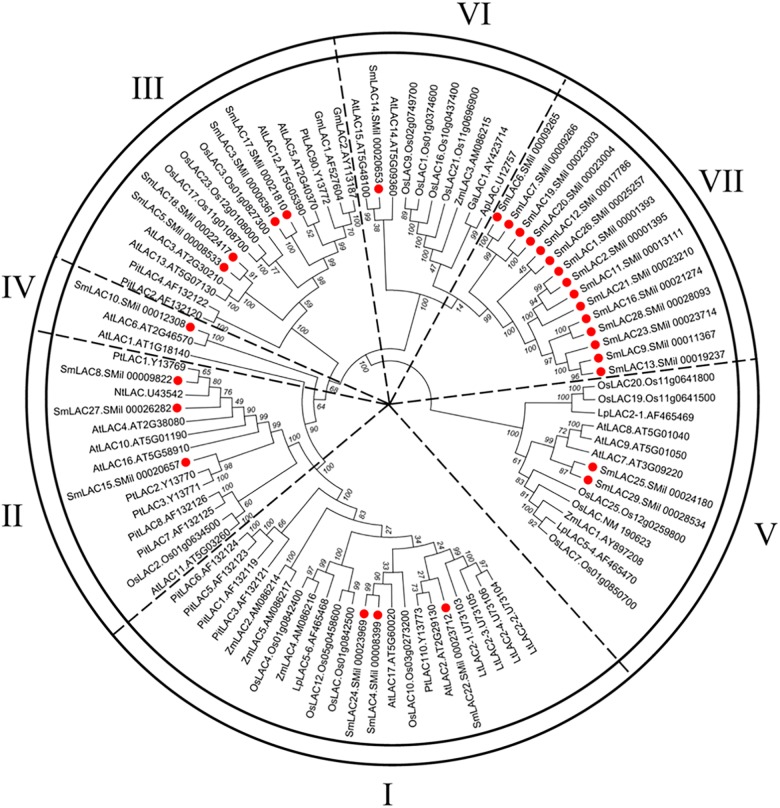
Neighbor-Joining (NJ) phylogenetic tree of laccases from *S. miltiorrhiza* and other 10 plants. The amino acid sequences are aligned using ClustalW, and the phylogenetic tree is constructed using neighbor-joining criteria in MEGA 5.05. The values on the branches are bootstrap proportions, which indicate the percentage values for obtaining this particular branching in 1000 repetitions of the analysis. Only bootstrap values larger than 50% support are indicated. The letters (I-VII) represent the main groups. The red dots represent laccases from *S. miltiorrhiza.*

To obtain the evolutionary relationships among the 29 SmLACs and other LACs from 10 selected plants (*Zea mays*, *Nicotiana tabacum*, *Populus trichocarpa*, *Pinus taeda*, *Gossypium arboreum*, *Glycine max*, *Acer pseudoplatanus*, *Liriodendron tulipifera*, *A. thaliana* and *Oryza sativa*), a neighbor-joining tree was constructed by MEGA 5.05 with 1000 bootstrap reconstruction and pairwise deletion gaps/missing data treatment and clustered into seven phylogenetic groups (Figure 4). Both group I and II contained 3 SmLACs respectively. Group III consisted of 4 SmLACs. There were 1, 2 and 1 SmLACs in group IV, V, and VI, respectively. Group VII included nearly half of the total SmLACs (15 SmLACs).

It is commonly accepted that proteins usually bear similar functions in their respective species if they share a high degree of sequence similarity. Although the functions of SmLACs are unknown, our construction of the SmLACs phylogenetic tree with different plants can help to derive the functions of SmLACs and lay a solid foundation for future functional studies. As shown in Figure 4, SmLAC10 shares 100% similarity with AtLAC6 and SmLAC14 shares 99% similarity with AtLAC15, strongly suggesting that SmLAC10 and SmLAC14 may have similar functions with AtLAC6 and AtLAC15 respectively. AtLAC15 is known involving in lignin synthesis, seed germination, root elongation (Liang et al., 2006a), oxidizing epicatechin into the oligomers as well as the synthesis of flavonoids in the seed coat of *A. thaliana* (Pourcel et al., 2005), SmLAC14 therefore may have the same functions in *S. miltiorrhiza*’s development. It was reported that when *AtLAC8* was knocked out, the flowering of plant was delayed and the number of the leaves was decreased (Cai et al., 2006). SmLAC25 and SmLAC29 are close to AtLAC8 in the phylogenetic tree, they might play similar roles in controlling the flowering and leaves growth of *S. miltiorrhiza* as AtLAC8 does in *Arabidopsis*. SmLAC4 and SmLAC24 are the closest homologs to AtLAC17 (up to 90% similarity), which was strongly expressed in *Arabidopsis*’s stems and participated in lignin synthesis (mainly participate in guaiacol radical accumulation) (Berthet et al., 2011), indicating that SmLAC4 and SmLAC24 may participate in *S. miltiorrhiza*’s lignin synthesis too. SmLAC22 is close to AtLAC2, whose function is related to root elongation in *Arabidopsis* (Cai et al., 2006). Group VII contains 15 SmLACs but without any members from other species, indicating that they might have species specificity and drive the unknown functions in *S. miltiorrhiza*.

**FIGURE 5:**
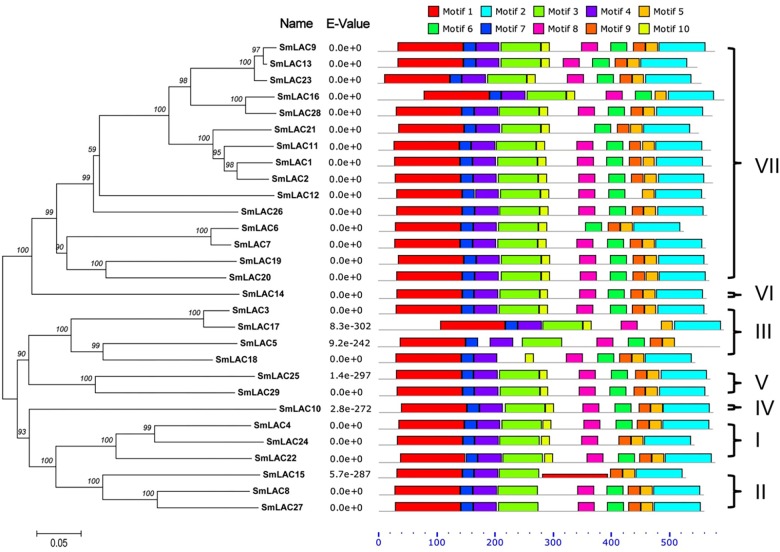
Distributions of conserved motifs in each SmLAC. The NJ phylogenetic tree and the combined *P-values* are shown on the left side of the figure. The relative positions of each conserved motif within SmLACs are shown in different colors.

To further analyze the sequence features of these 29 SmLAC proteins, a conserved motif search was conducted by MEME (Figure 5). The result suggested that most SmLAC proteins in the same group have similar motifs. Members in group IV, V, and VI contained 10 different types of conserved motifs. Eleven SmLACs in group VII held 10 kinds of motifs, while the rest four contained 9 motifs. Eight types of conserved motifs were found in some members of group II and group III (SmLAC15/5/17).

### Prediction of Diverse *cis* Regulatory Elements in SmLAC Promoters

Various numbers of putative *cis*-acting elements, including the core CAAT box and TATA box, were detected in the promoters of each *S. miltiorrhiza* LAC genes by PlantCARE (see Supplementary Table 7). All 29 SmLAC promoter sequences have many light responsive elements, such as G-box (Argüello Astorga and Herrera Estrella, 1998), revealing an essential role of SmLACs in plant morphogenesis. Besides, there are three types of representative DNA regulatory elements: stress responsive elements responding to diverse abiotic (anaerobic induction, defense and stress, cold and dehydration, heat stress, low temperature, drought, and wound) and biotic (fungal elicitor) stresses; hormone responsive elements involved in response to various plant hormones, such as ABA, MeJA, GA, SA, auxin, and ethylene; tissue specific expressed elements related to meristem-, endosperm-, seed- or shoot-specific activation and regulation. Moreover, two classes of MYB binding site elements (MBS I and MBS II), which are the flavonoid biosynthetic genes regulation sites were discovered in the promoters of five SmLAC genes (*SmLAC3/9/10/13/28*).

### Seven *SmLAC*s Were Found to Be Potential Targets of miR397

It is reported that ptr-miR397a is a negative regulator of LAC genes in *Populus trichocarpa* (Lu et al., 2013). Since miR397 sequence of *S. miltiorrhiza* was not available, ptr-miR397a from *P. trichocarpa* was used to search the transcript sequences of the 29 candidate *SmLAC*s, and 7 *SmLAC*s (*SmLAC8/24/4/27/5/25/29*) were predicted to be the potential ptr-miR397a targets (see Supplementary Table 8). Ssl-miR397 of *Salvia sclarea*, which is the congener plant of *S. miltiorrhiza* from the same genus, was also used to search the 29 *SmLAC*s transcript sequences, the same seven *SmLAC*s turned out to be the potential ssl-miR397 targets. Thus, *SmLAC8/24/4/27/5/25/29* may be negatively regulated by miR397 in *S. miltiorrhiza*.

### Differential Expression Profiles of *SmLACs* in Different Organs and Tissues

**FIGURE 6:**
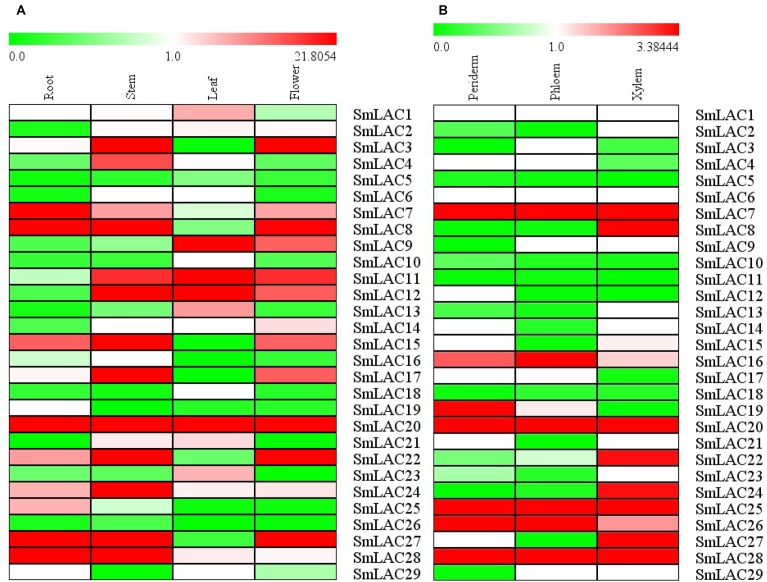
Expression patterns of 29 *SmLAC* genes in different organs and tissues. **(A)** different organs; **(B)** different tissues.

The relative constitutive abundance of the 29 *SmLACs* was quantified in roots, stems, leaves and flowers through Illumina and PacBio sequencing technology (Xu et al., 2015). Besides, the abundance of *SmLAC*s in different tissue parts of roots including periderm, phloem and xylem was also tested. The expression level of each *SmLAC* was estimated according to RPKM (reads per kilobase per million) values and presented in the heatmap in Figure 6. The results showed that the expression levels of the 29 *SmLAC*s varied with organs. For instance, *SmLAC7/8/20/27/28* were highly expressed in roots while *SmLAC3/8/11/12/15/17/20/22/24/27/28* were highly expressed in stems. *SmLAC9/11/12/20* were highly expressed in leaves, and *SmLAC3/8/20/22/27* were highly expressed in flowers. The expression level of *SmLAC20* was high in all the four organs. *SmLAC8* and *SmLAC27* were highly expressed in roots, stems and flowers. However, the expression level of 5 *SmLACs* (*SmLAC5*/*10*/*18/19*/*26*) was very low in the four organs.

As to the three different tissues of roots, 5 *SmLACs* (*SmLAC7/20/25/26/28*) displayed the highest transcript abundance in all xylem, epidermis and periderm. *SmLAC16* was found to be highly expressed in phloem followed by periderm and xylem. *SmLAC19* was highly expressed in periderm and more than in phloem and xylem. *SmLAC8/22/24/27* were highly expressed in xylem. These results indicated the functional conservation and diversity of *SmLAC*s.

### Confirmation of Five Highly Expressed *SmLACs* in Roots

**FIGURE 7:**
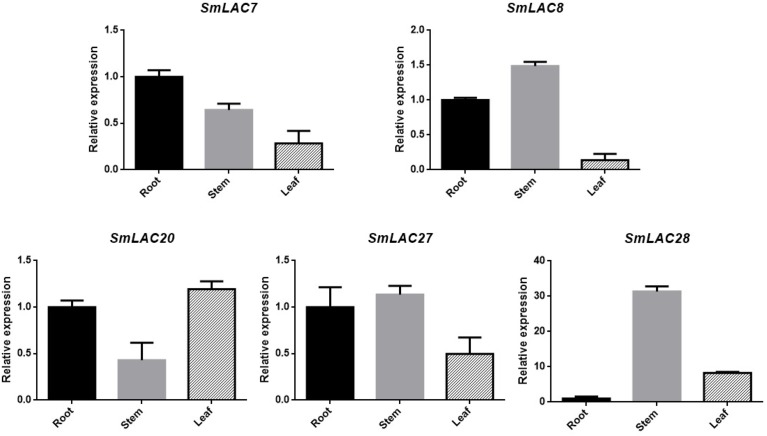
Expression patterns of 5 selected genes in different organs. The transcript levels are normalized by the results of that in roots. Each column is the average of three biological replicates.

Transcript abundance of a gene often correlates to its function. Considering the fact that hydrophilic compounds such as SAB of *S. miltiorrhiza* are more in roots than in other organs, the highly expressed five *SmLAC*s (*SmLAC7/8/20/27/28*) in roots may participate in salvianolic acids biosynthesis. To verify their expression levels, real-time PCR was performed (Figure 7) and the results showed that the expression levels of *SmLAC8*, *SmLAC27* and *SmLAC28* were higher in stems than in roots and leaves. The expression level of *SmLAC7* was high in roots, followed by stems and leaves. It was consistent with its expression in the heatmap. *SmLAC20* exhibited a high expression pattern in leaves and its expression in stems was much lower than that in roots and in leaves.

### Effects of Methyl Jasmonate on Expression of the Five Targeted *SmLAC*s

**FIGURE 8:**
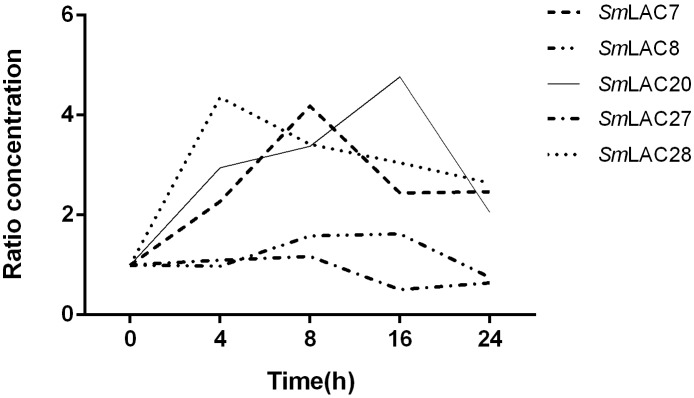
Relative expression of the 5 target genes subjected to MeJA treatment. The expression levels are normalized to corresponding values of MeJA treatments at 0 h. Each data point is the average of three biological replicates.

Methyl jasmonate (MeJA) has been used in plant cell engineering for inducing gene expression (Xiao et al., 2009). Previous studies have shown that genes in the metabolic pathway of *S. miltiorrhiza* can be significantly induced by MeJA and thus increase the content of SAB (Xiao et al., 2009). In order to obtain preliminary information about the effect of MeJA on *SmLAC*s, different expression levels of the five *SmLAC*s in MeJA treated hairy roots at different times, including 0, 4, 8, 16, and 24 h were analyzed through real-time PCR using gene-specific primers (see Supplementary Table 1). The results (Figure 8) showed that *SmLAC7*, *SmLAC20* and *SmLAC28* were significantly induced by MeJA, and the expressions of the genes were increased more than 3-fold. *SmLAC28* reached its peak at the 4th hour, while the maximum expressions of *SmLAC7* and *SmLAC20* appeared at the 8th and the 16th hour. However, there was no significant arising trend in *SmLAC8* and *SmLAC27*.

### Silencing of *SmLAC7, SmLAC20*, and *SmLAC28* in Hairy Roots of *S. miltiorrhiza*

**FIGURE 9:**
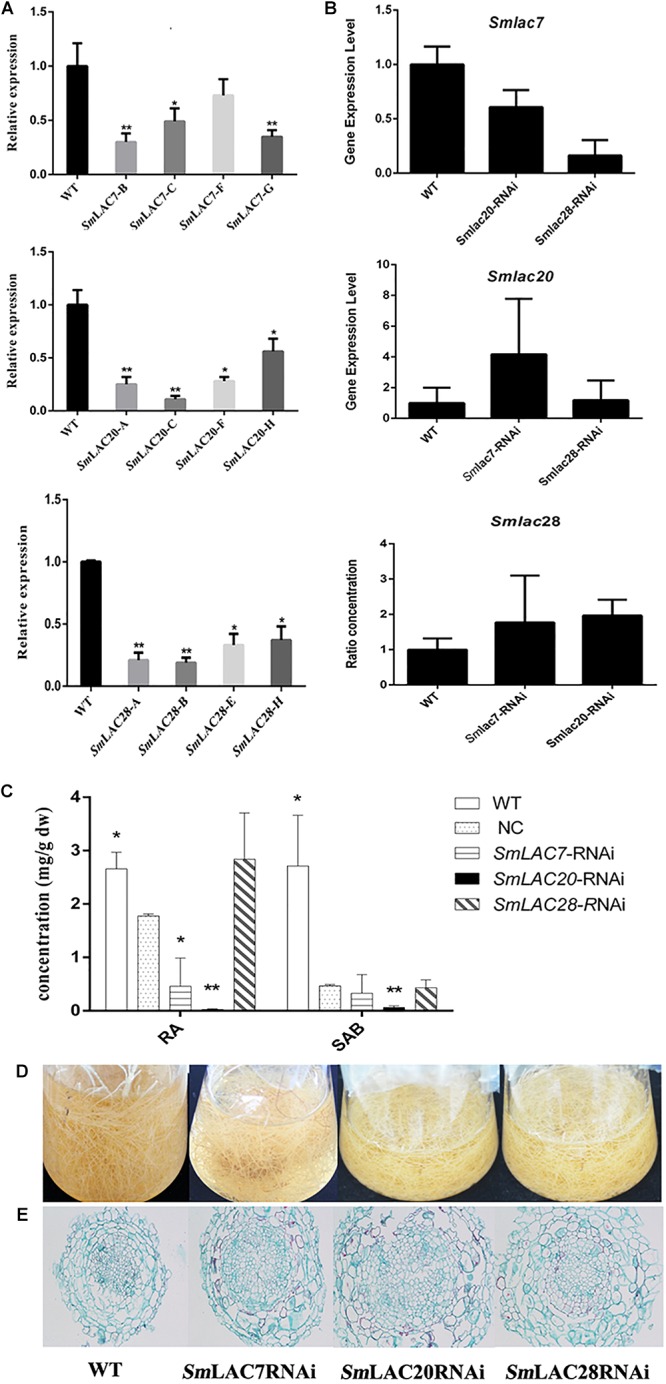
Comparative phenotypic analysis in wild type (WT) and *SmLACs*-RNAi transgenic hairy roots of *S. miltiorrhiza.* **(A)** Relative expression of *SmLAC7*, *SmLAC20*, and *SmLAC28* in WT and their correspondent lines of RNAi hairy roots. **(B)** The average transcript level of *SmLAC7*, *SmLAC20*, and *SmLAC28* in fellow RNAi hairy roots. The fellow groups are named on the horizontal axis. **(C)** The content of RA and SAB in the experimental hairy root cultures. The statistical differences are compared with NC. **(D)** Effects of laccases RNAi on the biomass of hairy root cultures. **(E)** Cross sections of hairy roots. The level of significance obtained by using the Student *t-test* is marked by the following: ^∗^*P* < 0.05, ^∗∗^*P* < 0.01, means ± SD, *n* = 3.

To explore the roles of MeJA responded genes (*SmLAC7*, *SmLAC20* and *SmLAC28*) in phenolic acid synthetic pathway in *S. miltiorrhiza*, RNAi transgenic hairy roots were generated by RNAi strategy under the control of the CaMV35S promoter. Real-time PCR was performed to confirm the transcript levels of these genes (Figure 9A). In contrast with the wild type (WT) line, the transcript levels of *SmLAC7*, *SmLAC20*, and *SmLAC28* were all decreased in the RNAi lines (Figure 9A). It also resulted in reductions of RA and SAB content in the *SmLAC7* or *SmLAC20* silenced lines (Figure 9C). The content of RA and SAB in the negative control (NC) line was dramatically different with the WT line. Since the NC line was induced by *A. tumefaciens* with the empty vector of RNAi, the accumulation of compounds might be affected. Therefore, the NC line was used as control. Compared to NC, the average reduction of SAB was 87% in line *SmLAC20*, 29.6% and 7.45% in line *SmLAC7* and SmLAC28, respectively (Figure 9C).

Besides, the transcriptional expression levels of the other two genes showed different performance in every single RNAi line. In *SmLAC7* silence line, the expression levels of *SmLAC20* and *SmLAC28* were higher than that in WT (4.18 folds and 1.78 folds respectively for the most), while in silence lines *SmLAC20* and *SmLAC28*, the expression of *SmLAC7* was lower than that in WT (about 0.6 folds and 0.16 folds of that in WT, respectively). However, the behavior of *SmLAC20* in *SmLAC28* silence line did not show obvious discrepancy when contrasted with that in WT, even though *SmLAC28* in *SmLAC20* silence line could reach to 1.97 folds compared with that in WT (Figure 9B).

To further investigate the putative impact of the transgenes at lignin biosynthesis, the vascular development of the hairy roots was inspected. The cross sections of hairy roots of both wild type and RNAi samples were dyed by Safranine O-Fast green FCF. The diameters of the transgenic cultures were much greater than that of the WT ones, so was the width of xylem (Figure 9E). More significantly, the xylem cells in RNAi samples appeared larger and looser than that in WT samples. There existed holes in the middle of the RNAi samples except the *SmLAC28* silence line. What’s more, the biomass of the hairy roots with *SmLACs* silenced in shake-flask cultures exhibited an obviously decreasing growth trend after 30 days cultivation compared with the wild type (Figure 9D).

### Overexpression of *SmLAC7* and *SmLAC20* in Hairy Roots of *S. miltiorrhiza*

**FIGURE 10:**
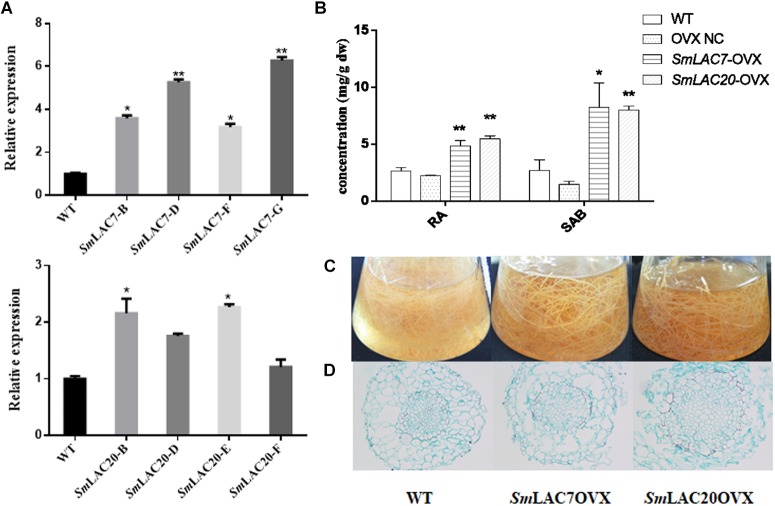
Comparative phenotypic analysis in wild type (WT) and *SmLACs* overexpressed transgenic hairy roots of *S. miltiorrhiza*. **(A)** Relative expression of *SmLAC7* and *SmLAC20* in WT and their correspondent lines of overexpression hairy roots. **(B)** The content of RA and SAB in the experimental hairy root cultures. **(C)** Effects of laccases overexpressed on the biomass of hairy root cultures. The statistical differences are compared with NC. **(D)** Cross sections of hairy roots. The level of significance obtained by using the Student *t-test* is marked by the following: ^∗^*P* < 0.05, ^∗∗^*P* < 0.01, means ± SD, *n* = 3.

*SmLAC7*, *SmLAC20*, and *SmLAC28* were all overexpressed in *S. miltiorrhiza* hairy roots. However, hairy roots with *SmLAC28* overexpressed didn’t grow well and were excluded during this analysis. To explore the *in vivo* roles of *SmLAC7* and *SmLAC20* in SAB biosynthesis pathway, their transcript levels were determined by real-time PCR and showed strikingly increase in the transgenic lines (Figure 10A), accordingly, the contents of RA and SAB (Figure 10B). The accumulation of SAB in *SmLAC20* overexpressed line was 5.45 folds higher than that of NC. It was 5.61 folds higher in *SmLAC7* overexpressed line than the NC one.

Unlike the LACs silencing lines, the *SmLAC7* and the *SmLAC20* overexpressed lines (Figure 10C,D) showed increased biomass, as well as larger diameter of vascular compared to WT lines. Interestingly, the lignification degree in LACs overexpressed hairy root lines was increased compared with the wild type. The area of xylem in the *SmLAC20* overexpressed lines turned to be larger than that in the wild type (Figure 10D).

## Discussion

As a multigene family, LACs widely exist in fungi, bacteria, plants and animals. Because of their special catalytic properties, function variations have been found in fungi and bacteria. However, functions of plant LACs are still poorly understood. Recent studies in structural and functional genomics in higher plant model species such as *Arabidopsis* revealed 17 LACs which are involved in stress response and lignin biosynthesis (Turlapati et al., 2011). The availability of the whole genome sequences of *S. miltiorrhiza* facilitates a comprehensive characterization of LAC genes in the commonly used herb. Here, we identified and characterized 29 LAC candidates in *S. miltiorrhiza*. They all exhibit the typical characteristics of three conserved Cu-oxidase domains, four signature sequences and 12 housed copper ligands.

Through utilization of a number of bioinformatics methods, we systematically analyzed all the 29 SmLACs. Although they all share similar coding domain structures, their sequence similarities are relatively low and intron–exon structures are quite different. Further differences in terms of number of amino acids and values of *p*I, implicate potentially functional divergence. In agreement with previous reports on *Arabidopsis* LACs (Turlapati et al., 2011), we find out that *S. miltiorrhiza* LAC genes are mostly located in the secretory pathway and contain glycosylation sites ensuring protein stability, folding, and formation of the cell wall.

Based on the phylogenetic relationships of *S. miltiorrhiza* and other ten plants, 29 SmLACs are mainly distributed into seven groups. The group VII contains 15 LACs all from *S. miltiorrhiza*, suggesting that these 15 SmLACs might hold species specificity. The homology of SmLAC8 and SmLAC27 is very close to AtLAC4. Also both SmLAC4 and SmLAC24 are just next to AtLAC17 in the phylogenetic tree. Since the two AtLACs function in lignin synthesis (Berthet et al., 2011; Zhao et al., 2013), we anticipate that all the four SmLAC4/8/24/27 participate in the synthesis of lignin in *S. miltiorrhiza.* This could be supported by the potential involvement of MYB58 in regulating *SmLAC4/8/24/27* since *SmLAC4/8/24/27* promoters contain the MYB binding site (MBS). MYB members MYB58 and MYB63 are known transcription activators in lignin biosynthesis, and MYB58 is particularly capable of activating *AtLAC4* (Zhou et al., 2009).

*SmLACs* may be negatively regulated by miR397. In *P. trichocarpa*, overexpressed ptr-miR397a negatively regulates LAC genes and decreases lignin content (Lu et al., 2013). We contrast the 29 *SmLAC*s with ptr-miR397a and ssl-miR397 one by one and find out that 7 *SmLAC*s (*SmLAC4/5/8/24/25/27/29*) can be combined with miR397 tightly, reflecting miR397’s roles in regulating the expressions of *SmLAC*s.

Expressions of the 29 LACs are tissues and organs dependent as supported by the analysis of transcriptome sequencing. We also observed SAB is mainly accumulated in the roots and the accumulation is positively correlated with the overexpressions of *Smlac7/8/20/27/28*, thus we speculate that these LACs may be involved in the synthesis of lignin and salvianolic acid in roots. This is supported by a combination of an early report that the content of SAB as well as RA are affected by MeJA (Xiao et al., 2009) and our current result that MeJA responsiveness motifs appear in the promoters of more than half *SmLAC*s (*SmLAC1/3/4/5/6/7/8/9/12/13/14/15/19/20/21/23/24/25/27*), including the four highly expressed genes (*SmLAC7/8/20/27*) in roots. And indeed, MeJA significantly affected the expressions of *SmLAC7, SmLAC20*, and *SmLAC28* based on the results of real-time PCR on MeJA treated hair roots of *S. miltiorrhiza*. What’s more, when the LAC in poplar is inhibited, its lignin component is not changed, but the phenolic metabolites are altered (Ranocha et al., 2002). This indicates that the highly expressed genes in roots likely participate in the biosynthesis of secondary metabolites in the phenylpropanoid pathway. Therefore, the three MeJA affected and highly expressed genes in roots were chosen for further study to illustrate their roles in the biosynthesis of SAB.

As expected, the contents of SAB in both *SmLAC7* and *SmLAC20* silenced lines were lower than in the wild type and negative control. Conversely, in the *SmLACs* overexpressed transgenic hairy root lines, SAB content increased with the expression of LACs. These observations strongly support that both *SmLAC7* and *SmLAC20* participate in the synthesis progress of SAB despite the exact SAB biosynthesis mechanism remains to be revealed.

In short, we comprehensively characterized LACs of *S. miltiorrhiza* and analyzed their molecular regulation functions. The results provide a solid ground for further exploring LACs in *S. miltiorrhiza* and other species as well. Our work adds to the knowledge for unveiling the formation of SAB and demonstrates a promising future in *S. miltiorrhiza* metabolic regulation in quality control.

## Author Contributions

QL and JF conceived the study. QL participated in data mining, data analysis, and proofreading the manuscript. JF performed the RNAi and overexpression experiment and wrote the manuscript. LC carried out the qRT-PCR experiment. ZX and YZhu provided the genome and transcription information of *S. miltiorrhiza.* YW, YZhou, and HT prepared the figures. YX initiated the project. JC helped to analysis the data. LZ participated in the design of the study. WC helped to conceive the study and participated in its design and coordination. All authors read and approved the final manuscript.

## Conflict of Interest Statement

The authors declare that the research was conducted in the absence of any commercial or financial relationships that could be construed as a potential conflict of interest. The reviewer YC declared a shared affiliation, with no collaboration, with one of the authors, YiZ, to the handling Editor at the time of the review.

## Footnotes

1 https://blast.ncbi.nlm.nih.gov/Blast.cgi?PROGRAM=blastp&PAGE_TYPE=BlastSearch&LINK_LOC=blasthome

2 http://web.expasy.org/protparam

3 http://www.cbs.dtu.dk/services/SignalP/

4 http://www.genscript.com/psort/wolf_psort.html

5 http://www.cbs.dtu.dk/services/TargetP/

6 http://www.cbs.dtu.dk/services/NetNGlyc/

7 http://www.cbs.dtu.dk/services/YinOYang/

8 http://www.cbs.dtu.dk/services/NetPhos/

9 http://gsds.cbi.pku.edu.cn/index.php

10 http://www.ebi.ac.uk/Tools/clustalw2/

11 http://npsa-pbil.ibcp.fr/cgi-bin/npsa_automat.pl?page=/NPSA/npsa_sopma.html

12 http://meme-suite.org/tools/meme

13 http://plantgrn.noble.org/psRNATarget/?function=3

14 http://bioinformatics.psb.ugent.be/webtools/plantcare/html/

15 http://primer3.ut.ee/

## Abbreviations

ABA: abscisic acid
GA: gibberellins
LAC: laccase
MeJA: methyl jasmonate
RA: rosmarinic acid
SA: salicylic acid
SAB: salvianolic acid B
