# Supplementary material for: Genome-Wide Identification and Characterization of Salvia miltiorrhiza Laccases Reveal Potential Targets for Salvianolic Acid B Biosynthesis
Source: Front Plant Sci. 2019 Apr 5;10:435. doi: 10.3389/fpls.2019.00435 (PMC6463009; doi:10.3389/fpls.2019.00435)
Supplement: Supplementary file 1 [file Data_Sheet_1.pdf]

Supplementary Material

Genome-wide identification and characterization of *Salvia miltiorrhiza* laccases reveal potential targets for salvianolic acid B biosynthesis

Qing Li[1#], Jingxian Feng[1#], Liang Chen[1], Zhichao Xu[2], Yingjie Zhu[3], Yun Wang[1], Ying Xiao[1], Junfeng Chen[1], Yangyun Zhou[1], Hexin Tan[4], Lei Zhang[4, 5*], Wansheng Chen[1*]

*** Correspondence:** Wansheng Chen chenwansheng@smmu.edu.cn; Lei Zhang zhanglei@smmu.edu.cn.

1 Supplementary Tables and Text

1.1 Supplementary Tables

Supplementary Table 1 Primer sequences used for quantitative real-time PCR.

Gene name	Forward primer (5'to3')	Reverse primer (5'to3')
SmLAC7	TTCCCTCAGAATCCGCCTTT	GCCTCTTCCGACCACATAGA
SmLAC8	AGGTGTATAGGCTCGCGTAC	TCTGTGGATCGGTTTTGGGA
SmLAC20	TAGGGGAGTGGTGGAAAAGTGATAT	GTGGTCCACACTCAGCTTGAACGTG
SmLAC27	GTCATCATCACGGCCAACAAG	TGCGGGATAGGTTTTGGAGT
SmLAC28	AGCACCCCATCAAAGTTCCT	GCGTGAAAACTCCTCCGATC
SmACTIN	AGGAACCACCGATCCAGACA	GGTGCCCTGAGGTCCTGGT

Supplementary Table 2. The gene-specific primers.

name		sequence
Primer 1		
pCambia-1300- Smlac7	F	AAGAGCTCTCTAGACTCGAGTACACCGGAAAC
	R	AAGGTACCGGATCCATCTTCCTCGTAGACTC
pCambia-1300- Smlac20	F	AACCATGGGTCGACAACCCTCCCCGATTTC
	R	AATCTAGACCATGGGGGCTCCACGGAGCCCATCTTC
pCambia-1300- Smlac28	F	AATCTAGACCATGGCCTCATCACTGGAGTTTC
	R	AAGGATCCGGTACCGAAGTCGAATATTGTTG
Primer 2		
pPHB-flag-Smlac7	F	AGGATCCATGAAGllTCTCATCGTGTAT
	R	AAACTAGTGCATCTGGGCATGTCTGCAG
pPHB-flag-Smlac20	F	AAAGATCTATGGGATCCACAAAGACGACGAG
	R	AAGCTAGCGCACATAGGGAAATCCGACGGT

Supplementary Table 3. Cu-oxidase domain contained genes in *S. miltiorrhiza* genome.

Pfam family accession	PF07732.9	PF00394.16	PF07731.8
Domain	Cu-oxidase_3	Cu-oxidase	Cu-oxidase_2
No.	79	80	80
Gene	SMil_00000484	SMil_00000484	SMil_00000484
	SMil_00001032	SMil_00001032	SMil_00001032
	SMil_00001393	SMil_00001393	SMil_00001393
	SMil_00001394	/	/
	SMil_00001395	SMil_00001395	SMil_00001395
	SMil_00003156	/	/
	SMil_00003157	SMil_00003157	SMil_00003157
	SMil_00003460	SMil_00003460	SMil_00003460
	SMil_00003461	SMil_00003461	SMil_00003461
	/	SMil_00004144	SMil_00004144
	/	/	SMil_00005175
	SMil_00005350	SMil_00005350	/
	/	/	SMil_00005351
	/	SMil_00005352	SMil_00005352
	SMil_00006094	SMil_00006094	SMil_00006094
	SMil_00006361	SMil_00006361	SMil_00006361
	SMil_00007218	SMil_00007218	SMil_00007218
	SMil_00007362	/	SMil_00007362
	/	SMil_00007673	SMil_00007673
	SMil_00008399	SMil_00008399	SMil_00008399
	SMil_00008477	/	/
	SMil_00008533	SMil_00008533	SMil_00008533
	SMil_00008578	SMil_00008578	SMil_00008578
	SMil_00009265	SMil_00009265	SMil_00009265
	SMil_00009266	SMil_00009266	/
	SMil_00009822	SMil_00009822	SMil_00009822
	SMil_00011134	/	/
	SMil_00011367	SMil_00011367	SMil_00011367
	/	/	SMil_00011750
	SMil_00012176	SMil_00012176	/
	/	SMil_00012308	SMil_00012308
	SMil_00012563	/	/
	/	SMil_00012564	/
	/	/	SMil_00012565
	SMil_00012566	/	/
	/	SMil_00012903	SMil_00012903
	SMil_00013111	SMil_00013111	SMil_00013111
	SMil_00013362	SMil_00013362	SMil_00013362

SMil_00014458	SMil_00014458	SMil_00014458
SMil_00014492	SMil_00014492	SMil_00014492
SMil_00014625	SMil_00014625	SMil_00014625
SMil_00014633	/	/
/	SMil_00014634	SMil_00014634
/	/	SMil_00014635
SMil_00014706	SMil_00014706	SMil_00014706
SMil_00014707	SMil_00014707	/
/	SMil_00016165	SMil_00016165
SMil_00016166	SMil_00016166	SMil_00016166
SMil_00016208	/	SMil_00016208
SMil_00017786	SMil_00017786	SMil_00017786
SMil_00018228	SMil_00018228	SMil_00018228
SMil_00018453	SMil_00018453	SMil_00018453
SMil_00019236	SMil_00019236	/
SMil_00019237	SMil_00019237	SMil_00019237
SMil_00020322	SMil_00020322	SMil_00020322
SMil_00020571	SMil_00020571	SMil_00020571
SMil_00020653	SMil_00020653	SMil_00020653
SMil_00020657	SMil_00020657	SMil_00020657
/	SMil_00020929	SMil_00020929
SMil_00021273	/	/
SMil_00021274	SMil_00021274	SMil_00021274
/	SMil_00021476	SMil_00021476
SMil_00021756	SMil_00021756	SMil_00021756
SMil_00021810	SMil_00021810	SMil_00021810
SMil_00022417	SMil_00022417	SMil_00022417
SMil_00022697	SMil_00022697	SMil_00022697
SMil_00023003	SMil_00023003	SMil_00023003
SMil_00023004	SMil_00023004	SMil_00023004
SMil_00023210	SMil_00023210	SMil_00023210
SMil_00023406	/	/
SMil_00023712	SMil_00023712	SMil_00023712
SMil_00023714	SMil_00023714	SMil_00023714
SMil_00023969	SMil_00023969	SMil_00023969
SMil_00023999	SMil_00023999	SMil_00023999
SMil_00024180	SMil_00024180	SMil_00024180
SMil_00024767	SMil_00024767	/
/	SMil_00024768	/
/	/	SMil_00024769
SMil_00025256	SMil_00025256	SMil_00025256
SMil_00025257	SMil_00025257	SMil_00025257
/	SMil_00026127	SMil_00026127
SMil_00026204	SMil_00026204	/

	/	/	SMil_00026205
	SMil_00026282	SMil_00026282	SMil_00026282
	SMil_00026302	/	SMil_00026302
	/	SMil_00026839	SMil_00026839
	SMil_00026840	SMil_00026840	SMil_00026840
	/	SMil_00027081	SMil_00027081
	SMil_00027743	SMil_00027743	SMil_00027743
	SMil_00028068	SMil_00028068	/
	SMil_00028093	SMil_00028093	SMil_00028093
	/	SMil_00028376	SMil_00028376
	SMil_00028534	SMil_00028534	SMil_00028534
	SMil_00028944	SMil_00028944	SMil_00028944
	SMil_00028975	/	/
	SMil_00029322	SMil_00029322	SMil_00029322
	SMil_00029573	SMil_00029573	SMil_00029573
	SMil_00029658	/	/
	SMil_00029822	SMil_00029822	SMil_00029822
	SMil_00030222	SMil_00030222	SMil_00030222
	SMil_00030284	SMil_00030284	SMil_00030284

Supplementary Table 4. Predicted protein homologs and domains of each SmLAC.

No.	Gene	Homolog	Status	Domains
1	SMil_00000484	monocopper oxidase-like protein	Non	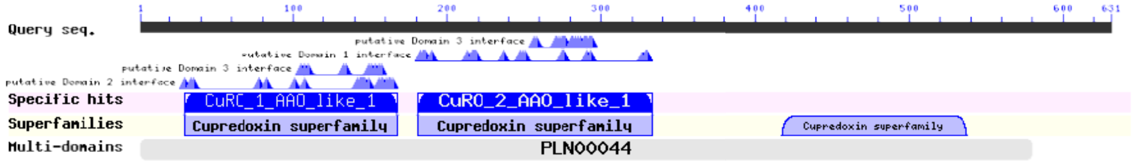
2	SMil_00001032	L-ascorbate oxidase homolog	Non	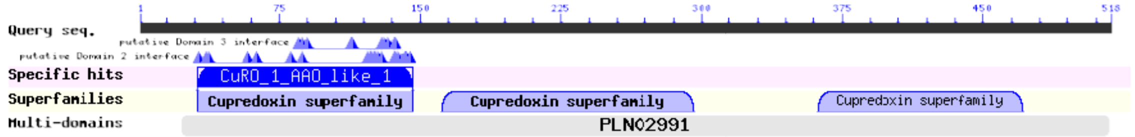
3	SMil_00001393	laccase	Full-length	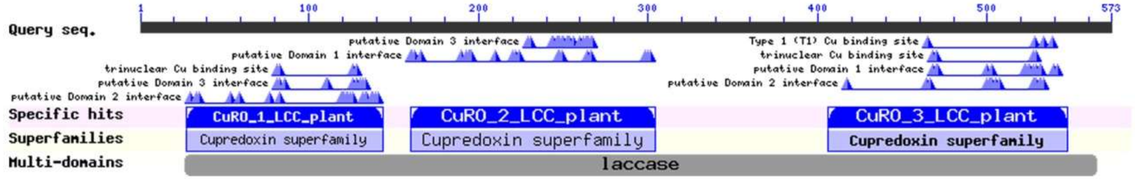
4	SMil_00001394	laccase	Partial	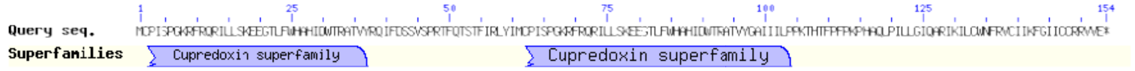
5	SMil_00001395	laccase	Full-length	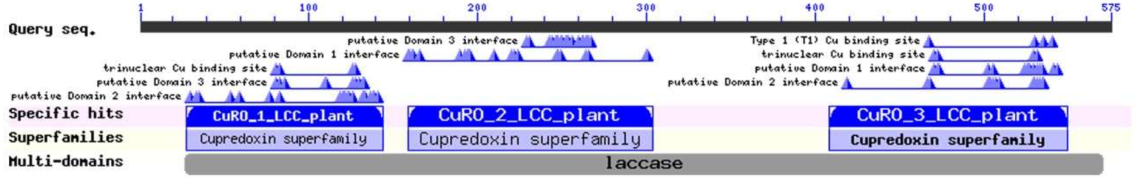
6	SMil_00003156	L-ascorbate oxidase homolog	Non	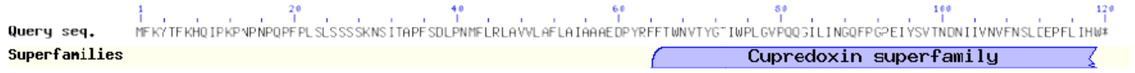

7	SMil_00003157	L-ascorbate oxidase homolog	Non	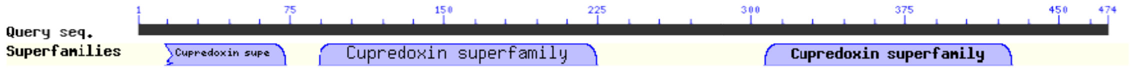
8	SMil_00003460	L-ascorbate oxidase homolog	Non	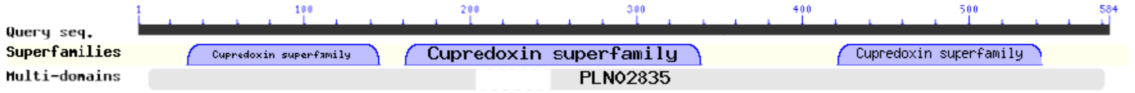
9	SMil_00003461	L-ascorbate oxidase homolog	Non	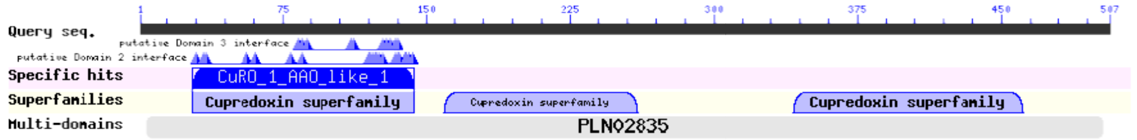
10	SMil_00004144	laccase	Partial	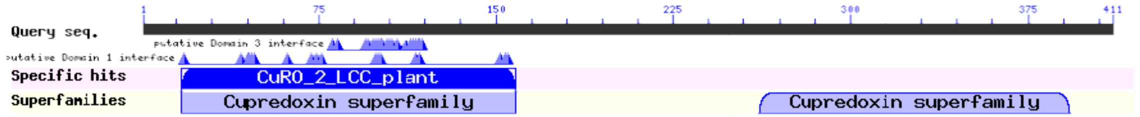
11	SMil_00005175	laccase	Partial	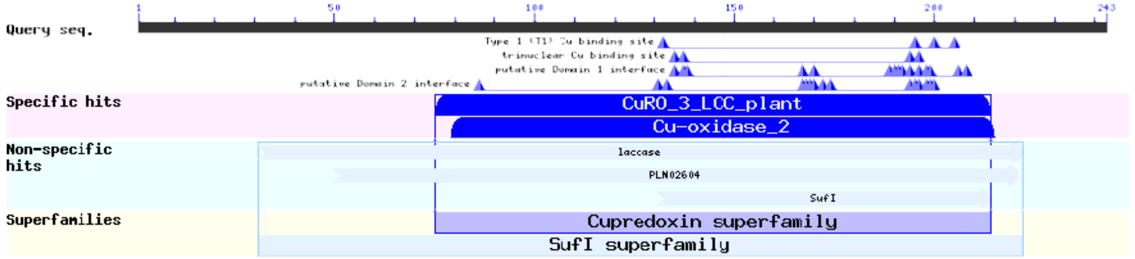
12	SMil_00005350	laccase	Partial	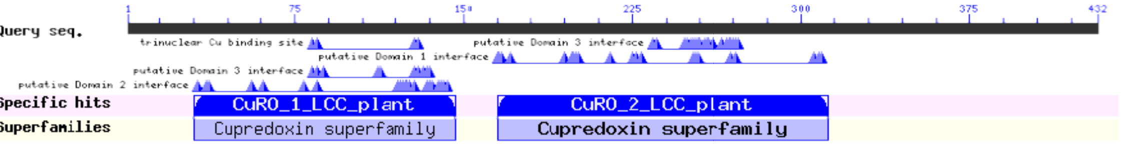
13	SMil_00005351	laccase	Partial	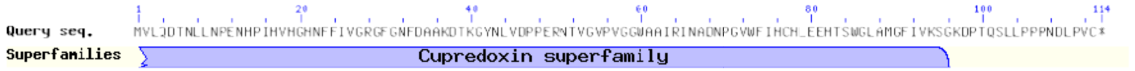

14	SMil_00005352	laccase	Partial	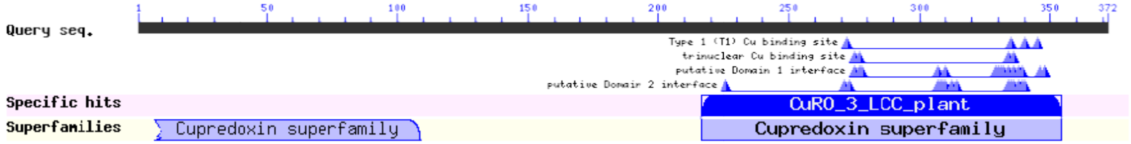
15	SMil_00006094	L-ascorbate oxidase homolog	Non	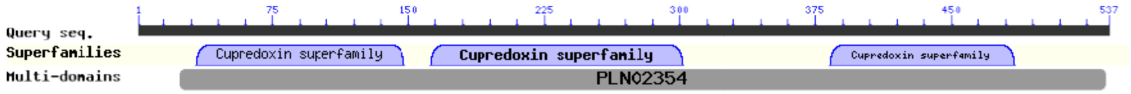
16	SMil_00006361	laccase	Full-length	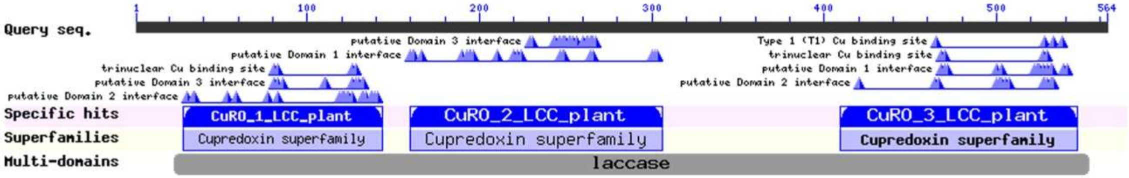
17	SMil_00007218	L-ascorbate oxidase homolog	Non	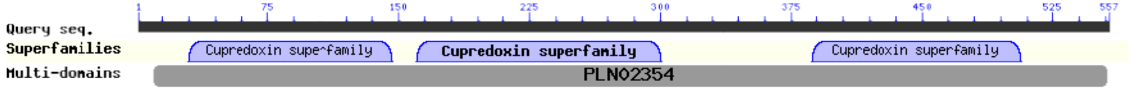
18	SMil_00007362	L-ascorbate oxidase-like	Non	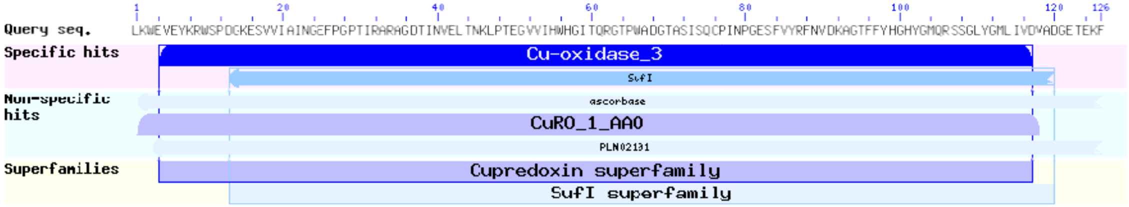

19	SMil_00007673	L-ascorbate oxidase homolog	Non	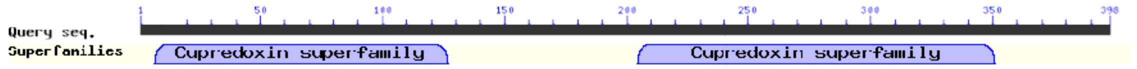
20	SMil_00008399	laccase	Full-length	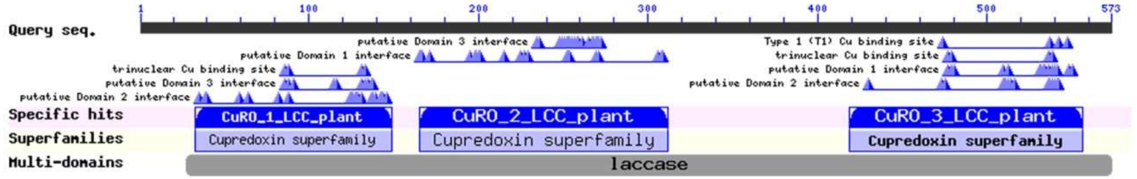
21	SMil_00008477	laccase	Partial	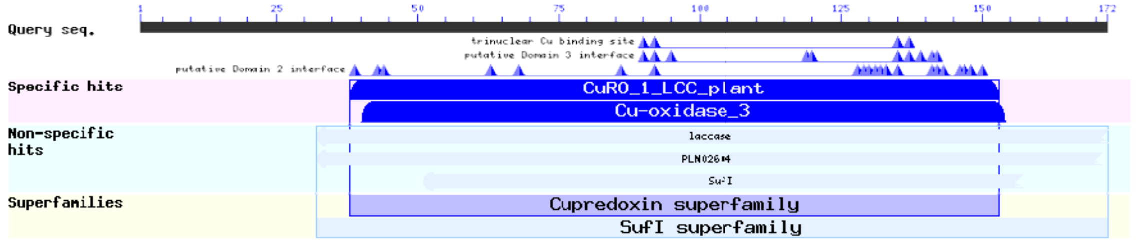
22	SMil_00008533	laccase	Full-length	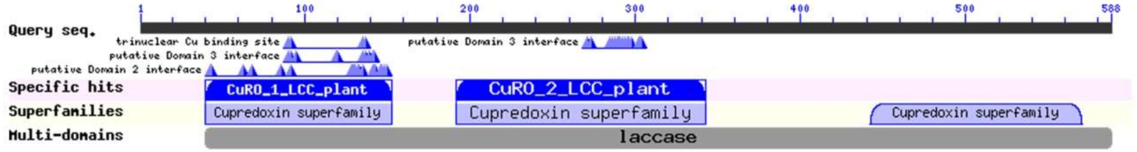
23	SMil_00008578	laccase	Partial	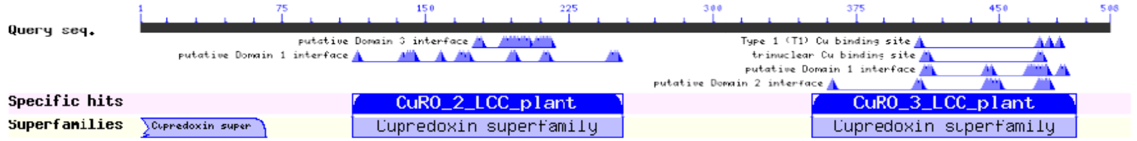
24	SMil_00009265	laccase	Full-length	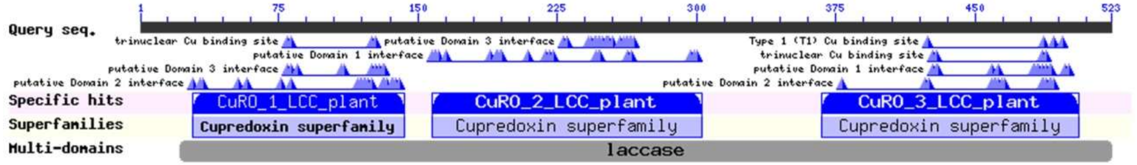

25	SMil_00009266	laccase	Full-length	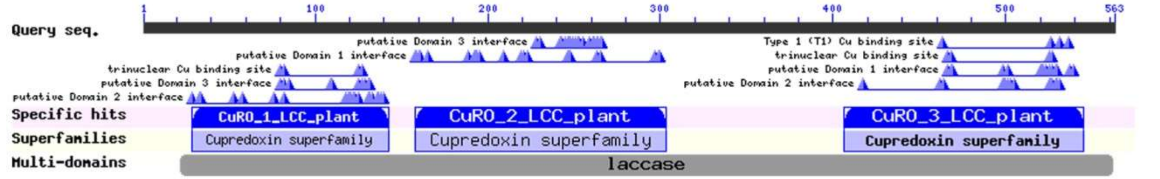
26	SMil_00009822	laccase	Full-length	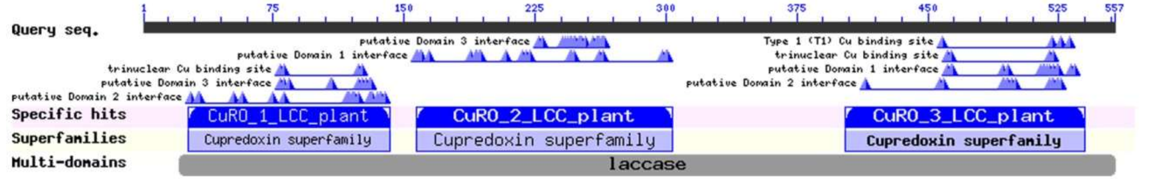
27	SMil_00011134	laccase	Partial	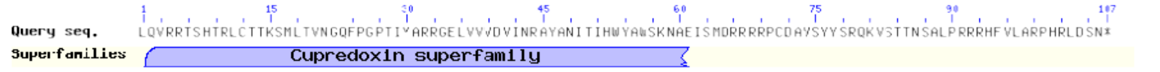
28	SMil_00011367	laccase	Full-length	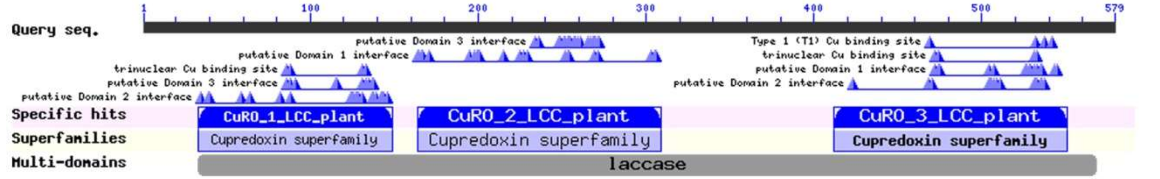
29	SMil_00011750	laccase	Partial	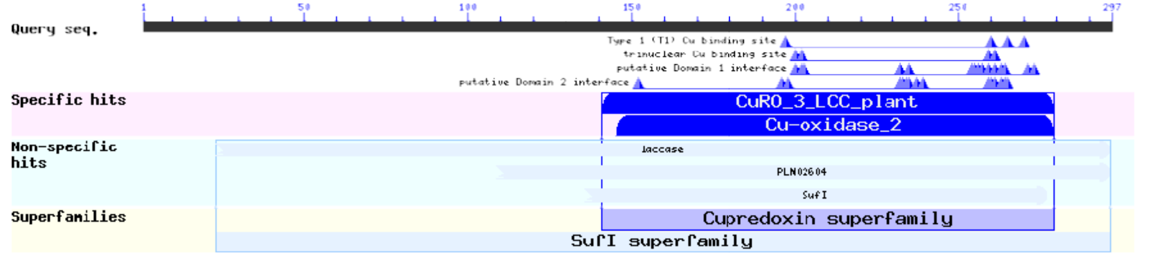

30	SMil_00012176	laccase	Partial	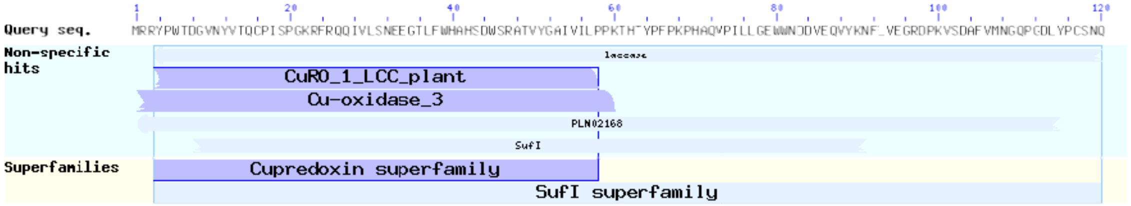
31	SMil_00012308	laccase	Full-length	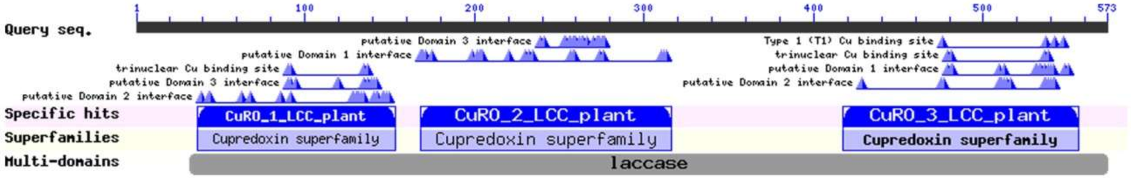
32	SMil_00012563	laccase	Partial	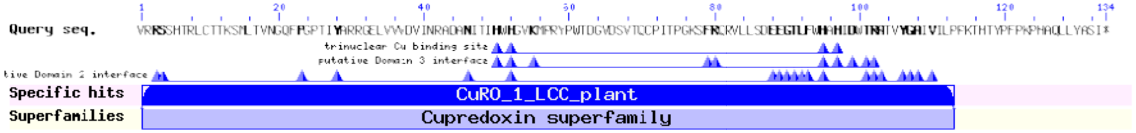
33	SMil_00012564	laccase	Partial	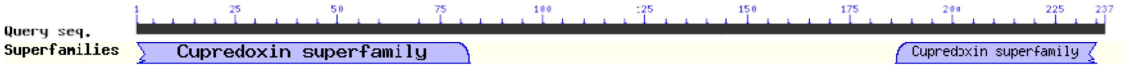
34	SMil_00012565	laccase	Partial	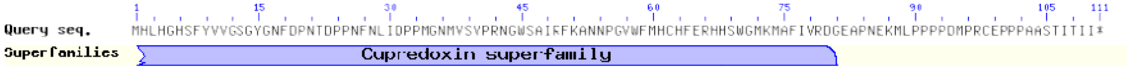
35	SMil_00012566	laccase	Partial	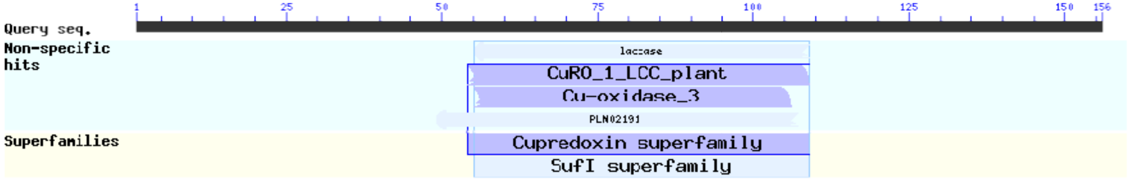

36	SMil_00012903	laccase	Partial	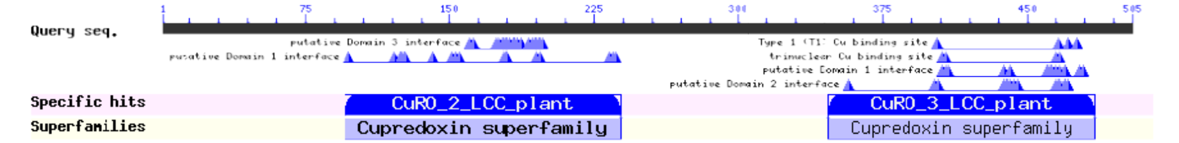
37	SMil_00013111	laccase	Full-length	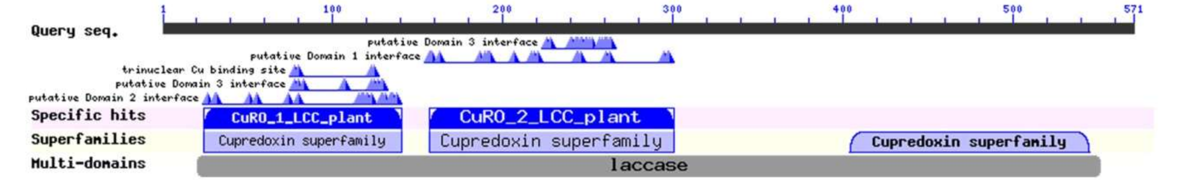
38	SMil_00013362	L-ascorbate oxidase homolog	Non	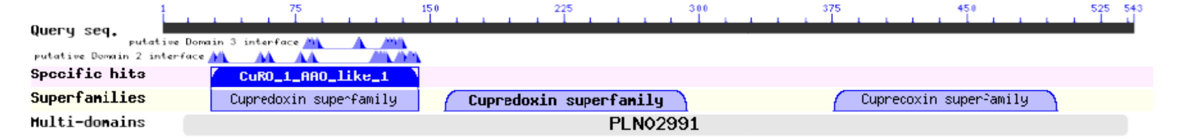
39	SMil_00014458	multicopper oxidase	Non	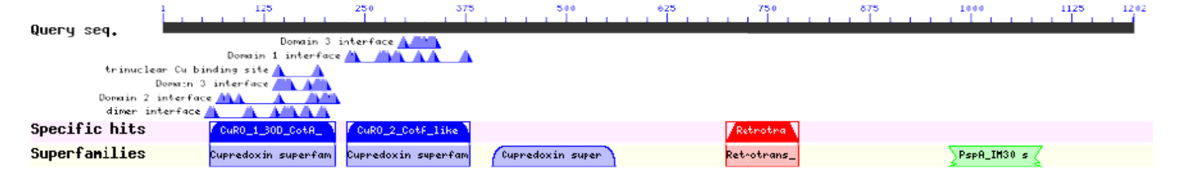
40	SMil_00014492	L-ascorbate oxidase homolog	Non	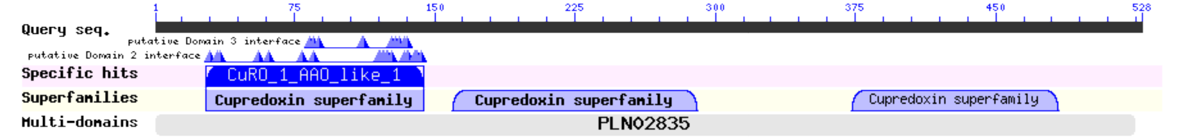
41	SMil_00014625	monocopper oxidase-like protein	Non	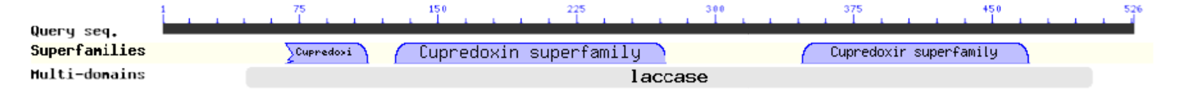

42	SMil_00014633	monocopper oxidase-like protein	Non	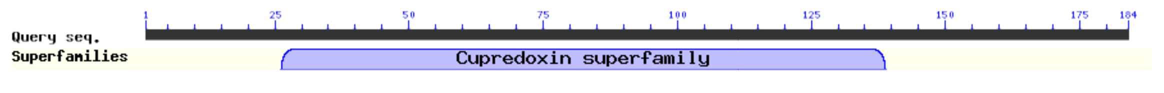
43	SMil_00014634	monocopper oxidase-like protein	Non	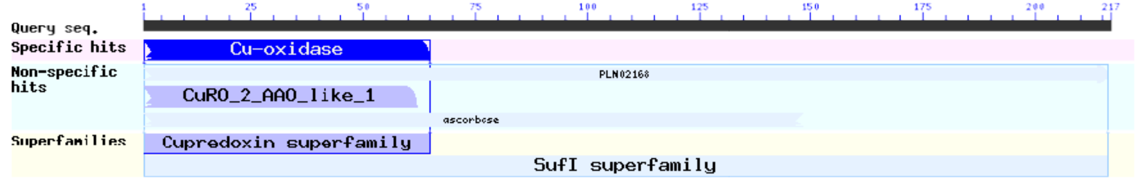
44	SMil_00014635	monocopper oxidase-like protein	Non	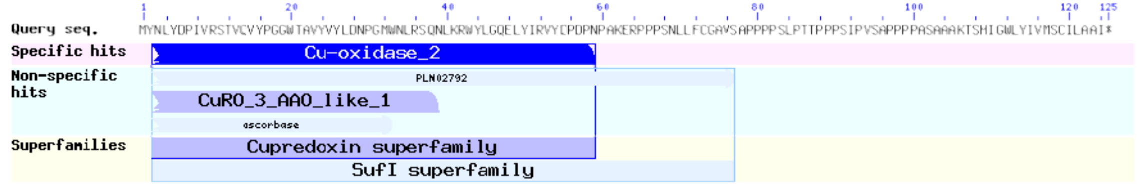
45	SMil_00014706	laccase	Partial	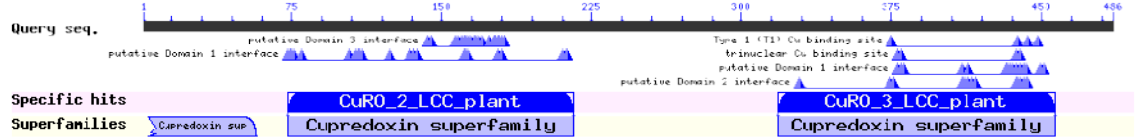
46	SMil_00014707	laccase	Partial	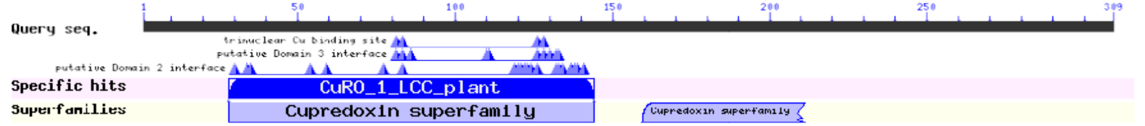
47	SMil_00016165	laccase	Partial	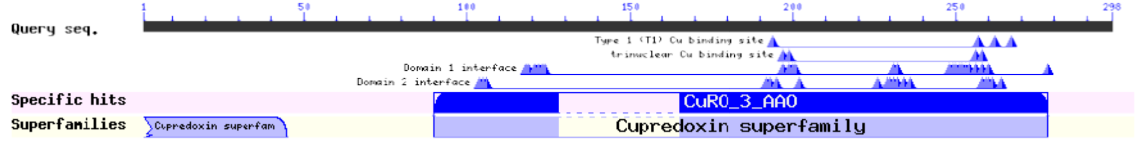

48	SMil_00016166	L-ascorbate oxidase-like protein	Non	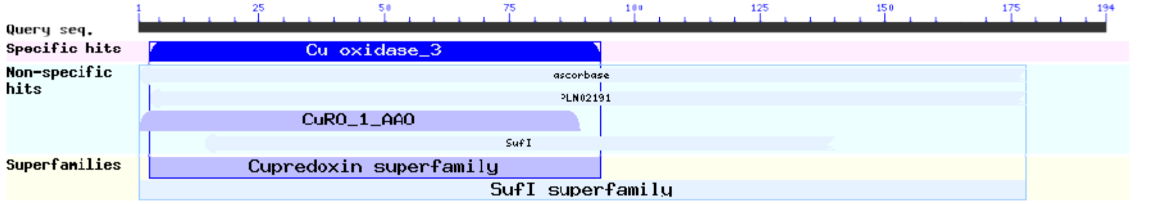
49	SMil_00016208	laccase	Partial	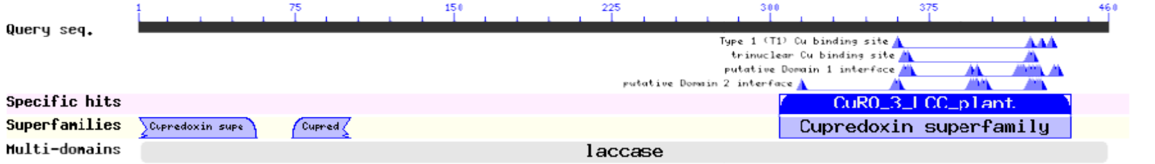
50	SMil_00017786	laccase	Full-length	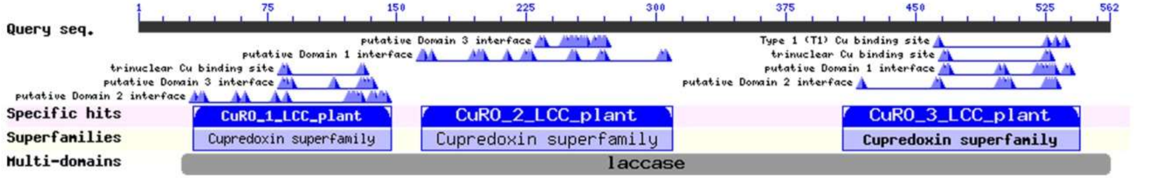
51	SMil_00018228	L-ascorbate oxidase homolog	Non	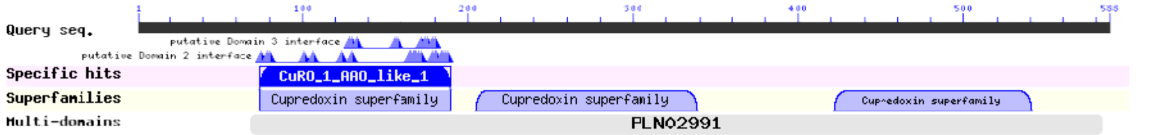
52	SMil_00018453	L-ascorbate oxidase-like protein	Non	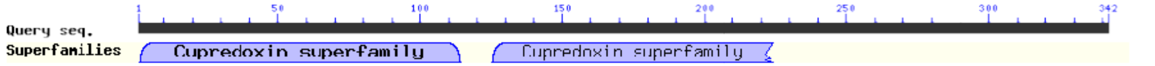
53	SMil_00019236	laccase	Partial	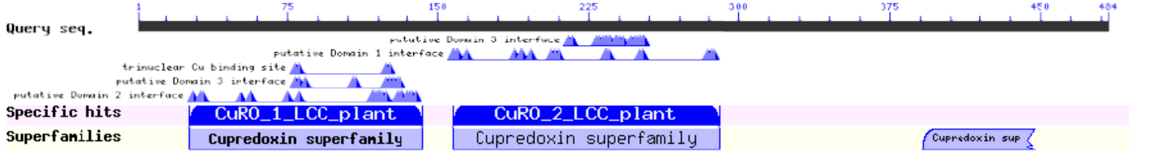

54	SMil_00019237	laccase	Full-length	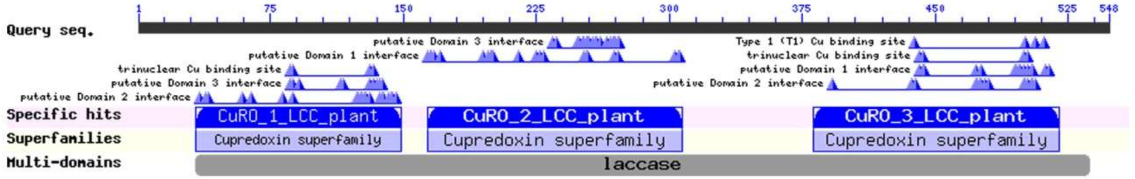
55	SMil_00020322	L-ascorbate oxidase homolog	Non	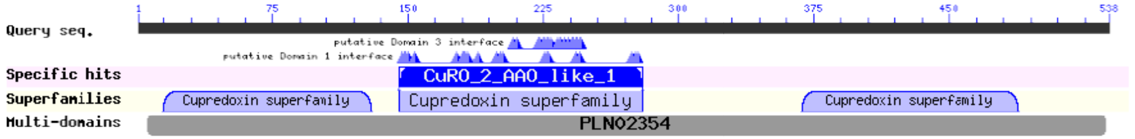
56	SMil_00020571	L-ascorbate oxidase homolog	Non	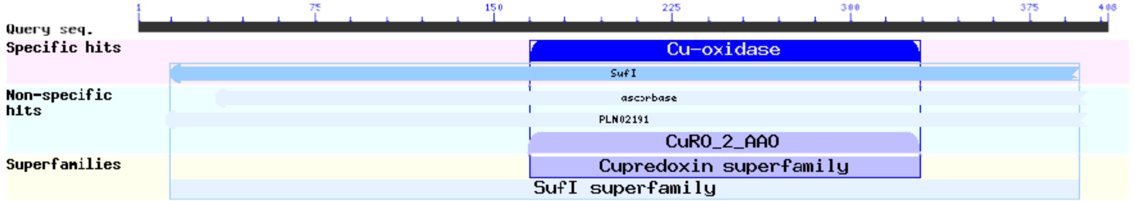
57	SMil_00020653	laccase	Full-length	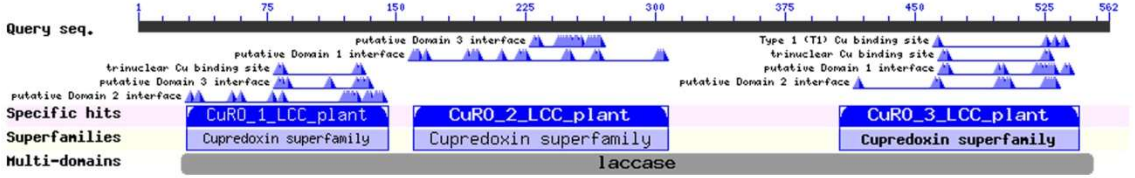
58	SMil_00020657	laccase	Full-length	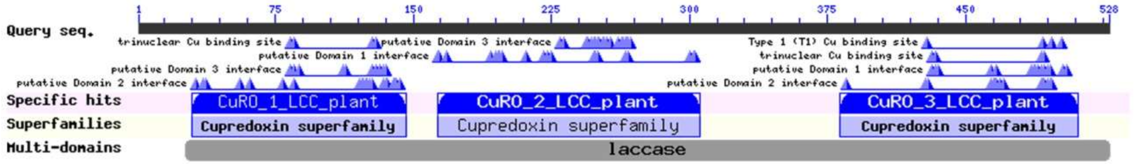

59	SMil_00020929	laccase	Partial	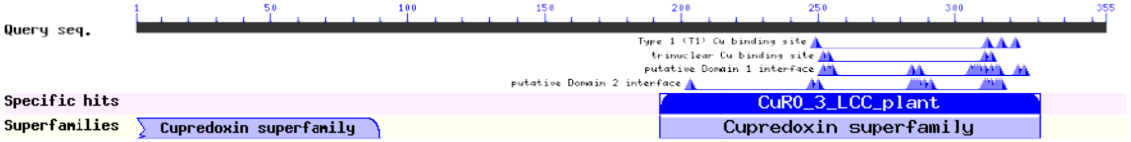
60	SMil_00021273	laccase	Partial	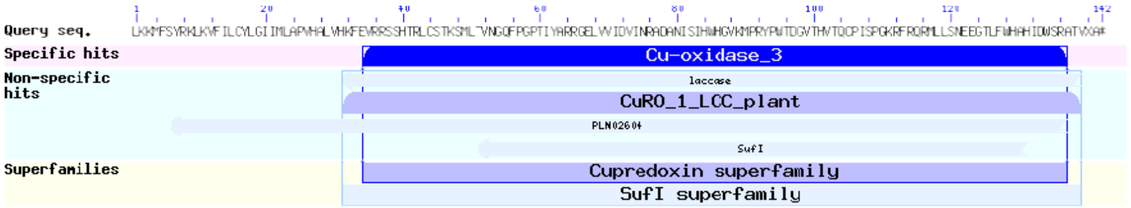
61	SMil_00021274	laccase	Full-length	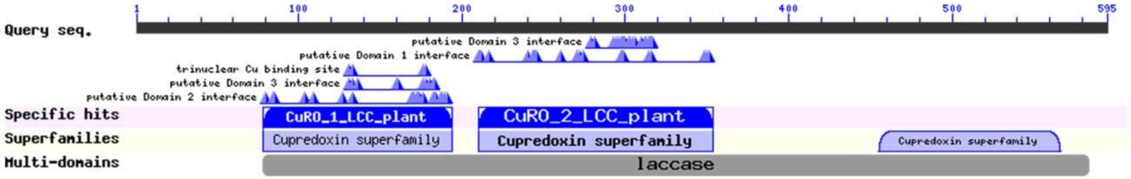
62	SMil_00021476	laccase	Partial	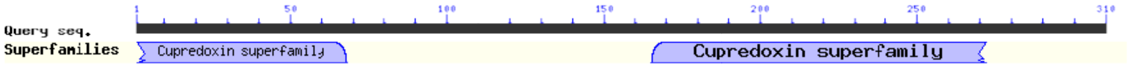
63	SMil_00021756	monocopper oxidase-like protein	Non	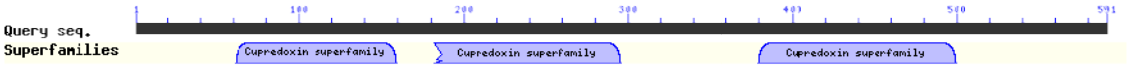
64	SMil_00021810	laccase	Full-length	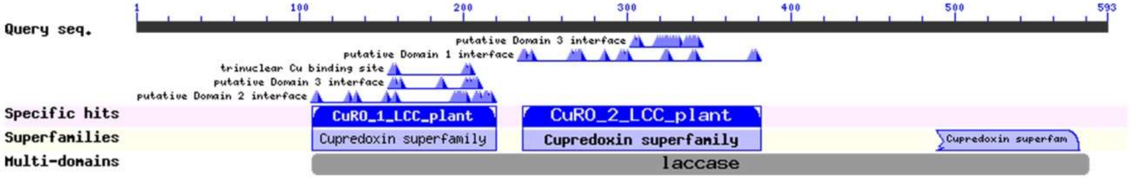

65	SMil_00022417	laccase	Full-length	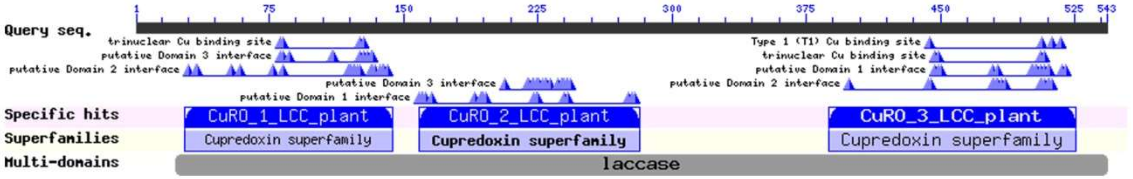
66	SMil_00022697	laccase	Partial	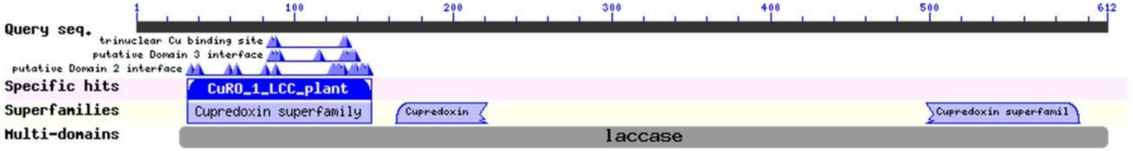
67	SMil_00023003	laccase	Full-length	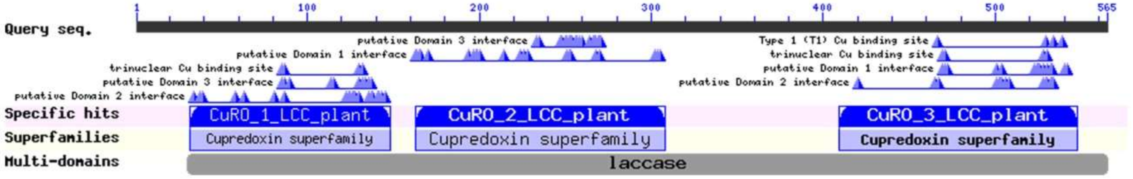
68	SMil_00023004	laccase	Full-length	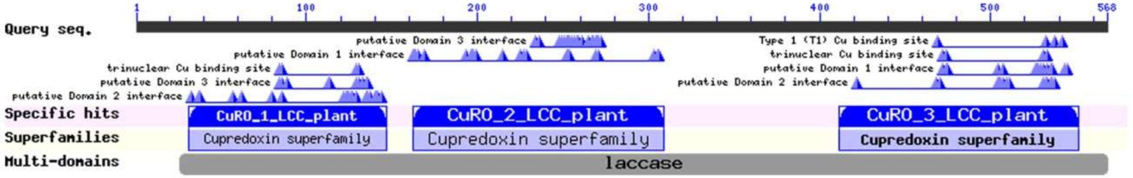
69	SMil_00023210	laccase	Full-length	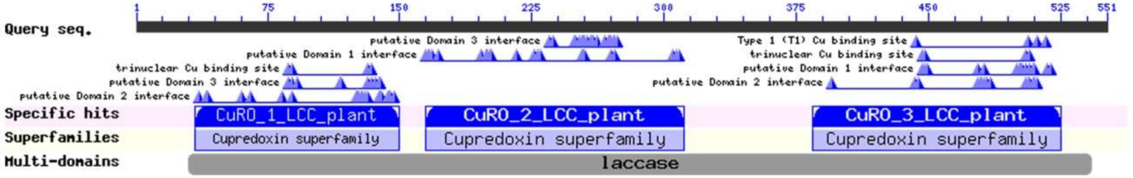
70	SMil_00023406	L-ascorbate oxidase-like protein	Non	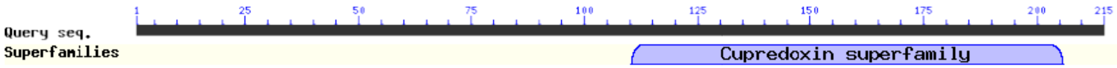

71	SMil_00023712	laccase	Full-length	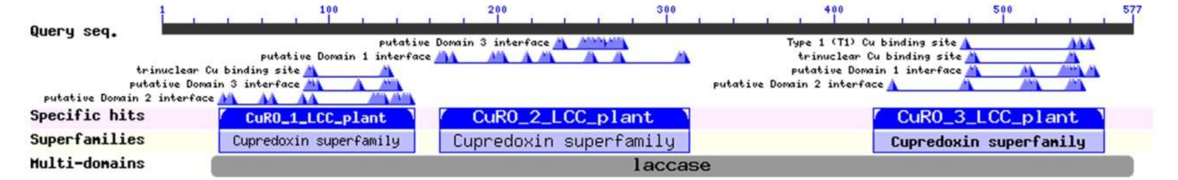
72	SMil_00023714	laccase	Full-length	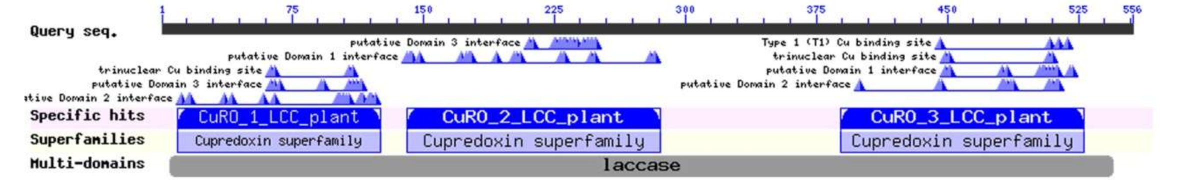
73	SMil_00023969	laccase	Full-length	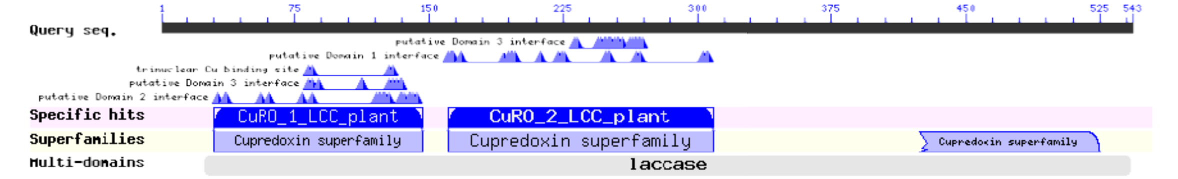
74	SMil_00023999	L-ascorbate oxidase homolog	Non	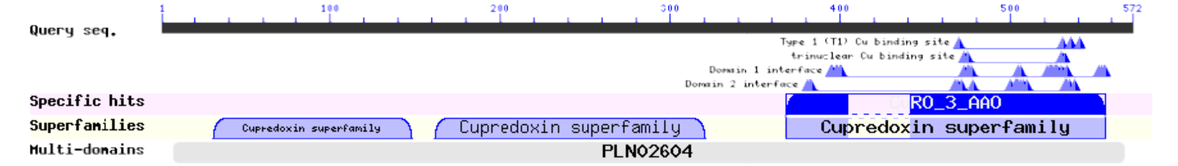
75	SMil_00024180	laccase	Full-length	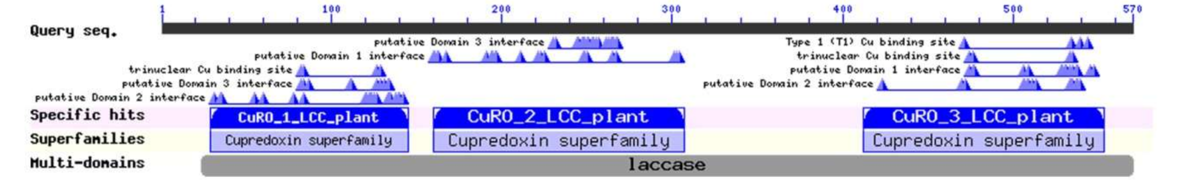

76	SMil_00024767	laccase	Partial	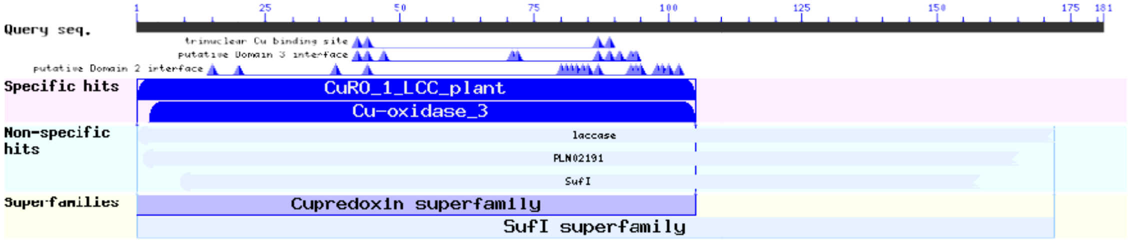
77	SMil_00024768	laccase	Partial	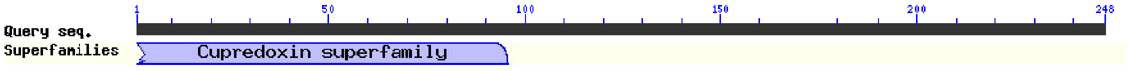
78	SMil_00024769	laccase	Partial	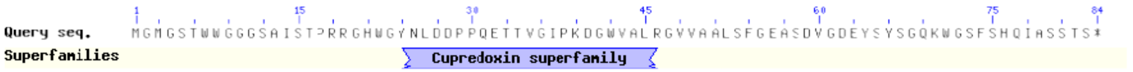
79	SMil_00025256	laccase	Partial	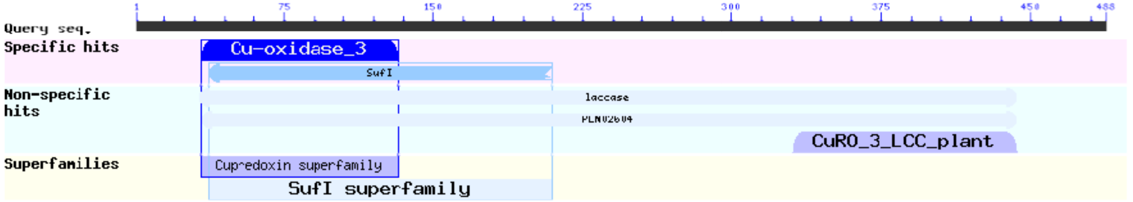
80	SMil_00025257	laccase	Full-length	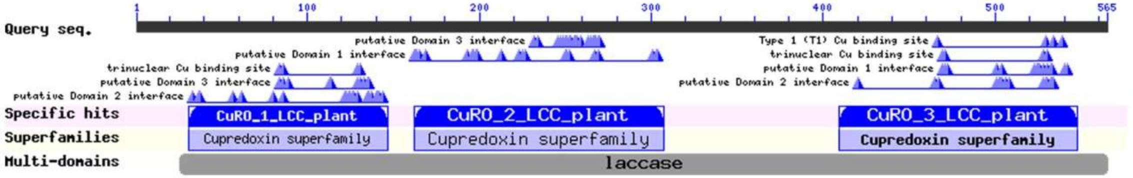
81	SMil_00026127	laccase	Partial	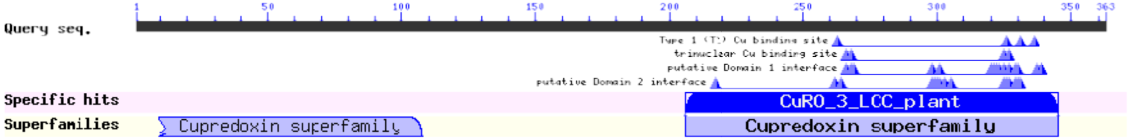

82	SMil_00026204	L-ascorbate oxidase homolog	Non	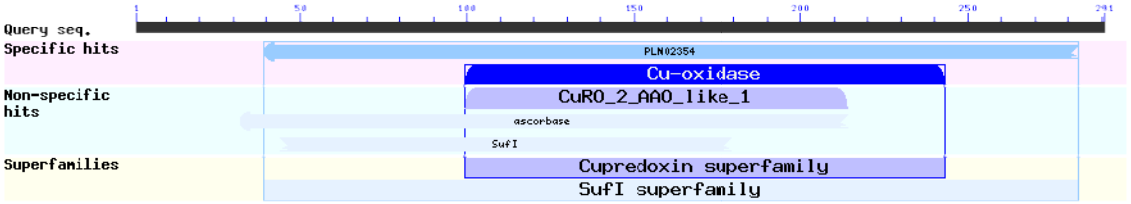
83	SMil_00026205	L-ascorbate oxidase homolog	Non	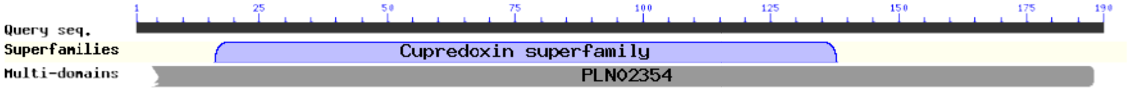
84	SMil_00026282	laccase	Full-length	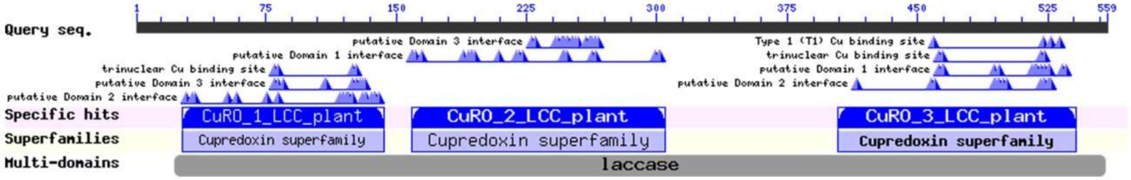
85	SMil_00026302	laccase	Partial	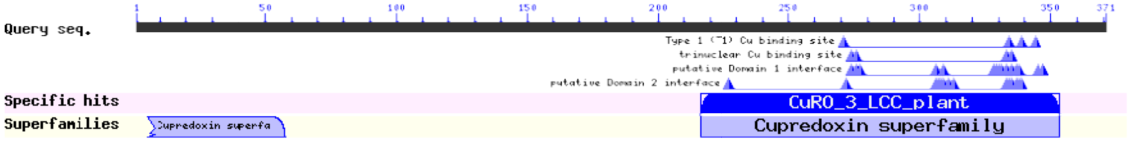
86	SMil_00026839	laccase	Partial	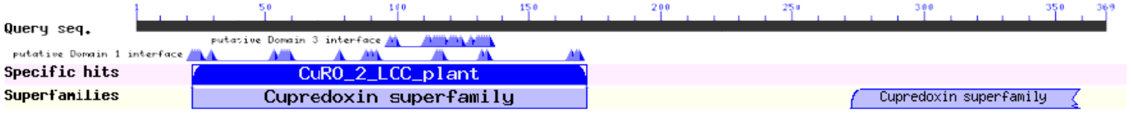
87	SMil_00026840	laccase	Partial	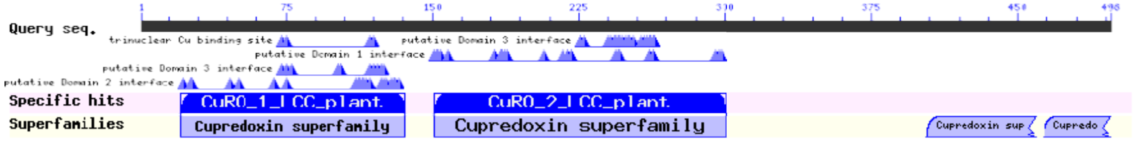

88	SMil_00027081	laccase	Partial	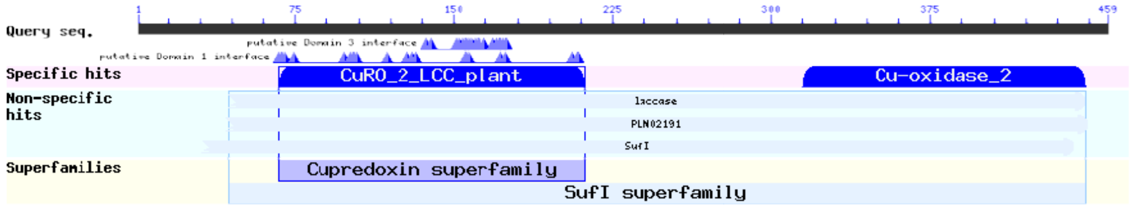
89	SMil_00027743	monocopper oxidase-like protein	Non	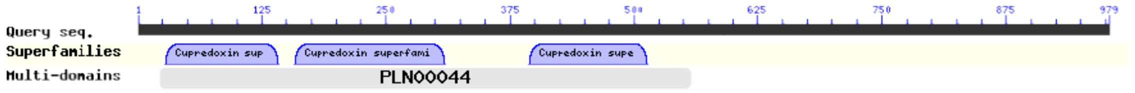
90	SMil_00028068	laccase	Partial	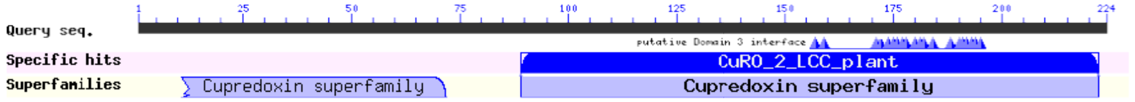
91	SMil_00028093	laccase	Full-length	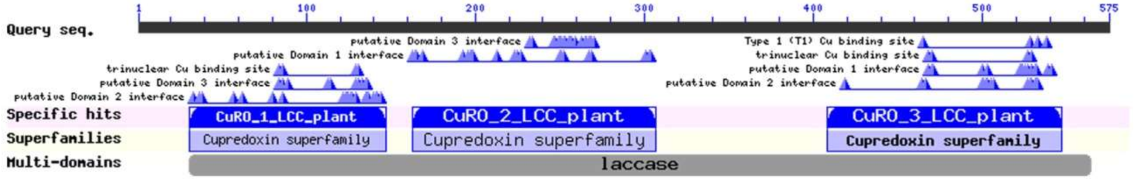
92	SMil_00028376	laccase	Partial	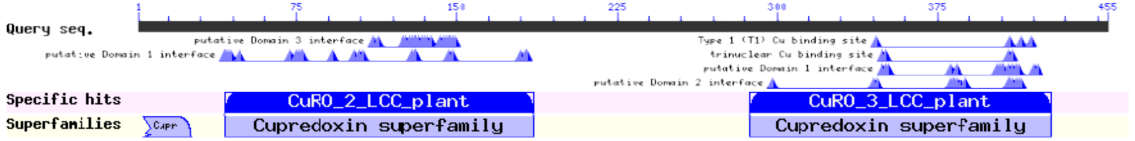
93	SMil_00028534	laccase	Full-length	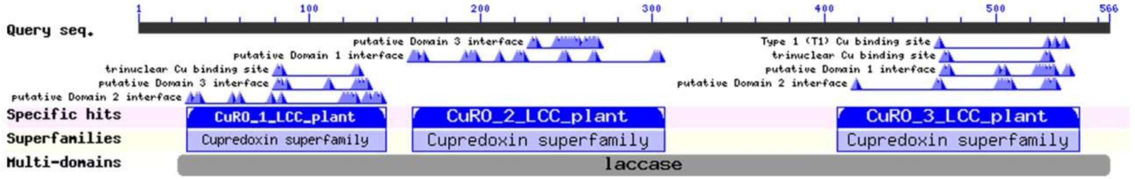

94	SMil_00028944	L-ascorbate oxidase	Non	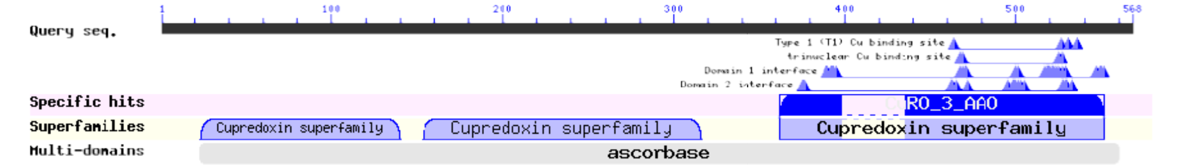
95	SMil_00028975	laccase	Partial	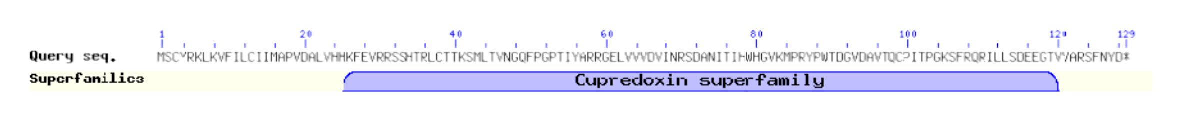
96	SMil_00029322	laccase	Partial	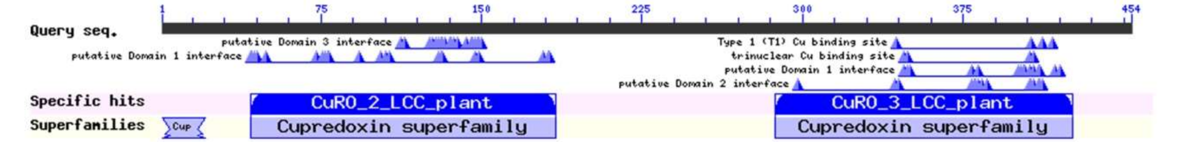
97	SMil_00029573	L-ascorbate oxidase-like protein	Non	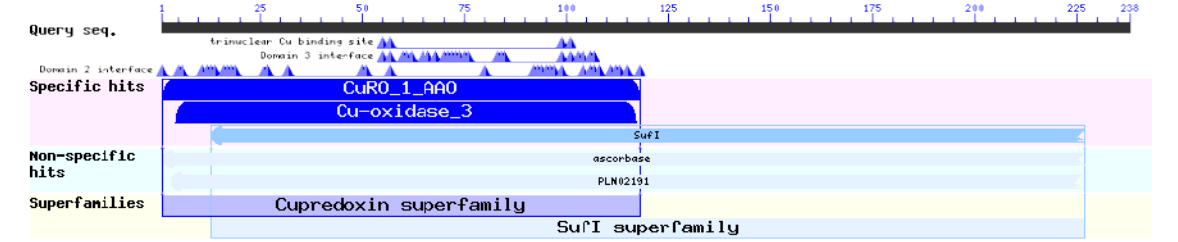
98	SMil_00029658	laccase	Partial	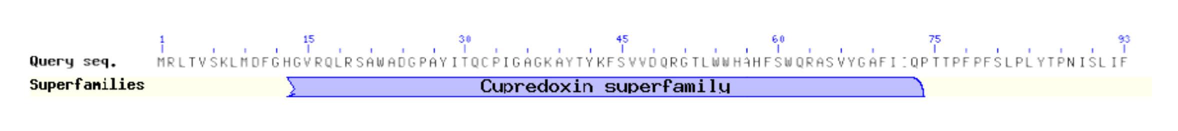
99	SMil_00029822	monocopper oxidase-like protein	Non	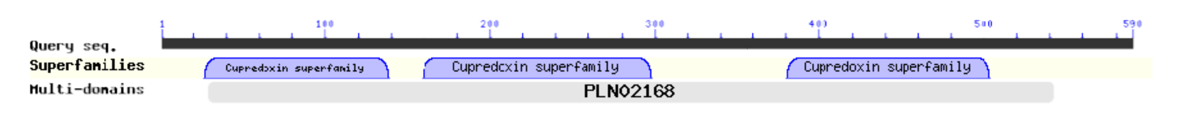
100	SMil_00030222	laccase	Partial	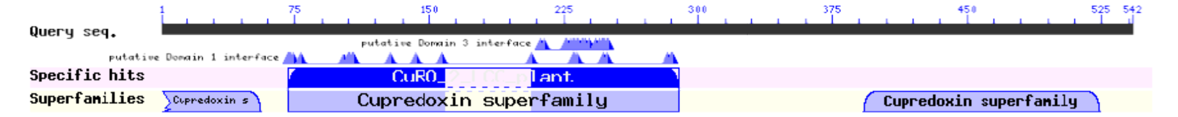

101	SMil_00030284	laccase	Partial	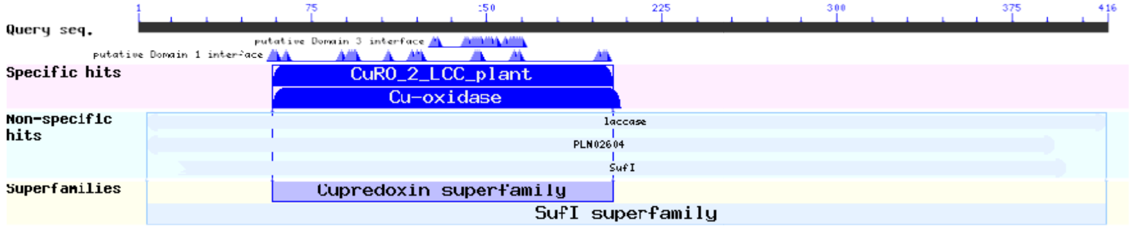

Supplementary Table 5. Comparison of secondary structures in 29 SmLACs.

Name	α-helix (%)	β-turn (%)	Extended strand (%)	Random coil (%)
SmLAC1	17.10	9.08	28.97	44.85
SmLAC2	15.13	11.48	27.65	45.74
SmLAC3	19.68	9.22	28.37	42.73
SmLAC4	19.02	9.60	28.80	42.58
SmLAC5	21.43	8.67	29.93	39.97
SmLAC6	17.02	10.71	29.83	42.45
SmLAC7	20.46	9.43	28.65	41.46
SmLAC8	18.85	9.52	30.52	41.11
SmLAC9	19.69	10.88	27.63	41.80
SmLAC10	17.45	11.52	31.76	39.27
SmLAC11	18.04	10.33	27.50	44.13
SmLAC12	20.64	10.68	29.00	39.68
SmLAC13	18.25	10.40	29.56	41.79
SmLAC14	14.77	12.10	30.78	42.35
SmLAC15	20.64	9.66	26.52	43.18
SmLAC16	18.82	12.61	27.90	40.67
SmLAC17	16.86	9.27	27.15	46.71
SmLAC18	14.55	8.66	32.23	44.57
SmLAC19	17.70	10.44	28.32	43.54
SmLAC20	16.90	10.04	28.87	44.19
SmLAC21	16.52	9.26	27.95	46.28
SmLAC22	16.64	9.36	29.81	44.19
SmLAC23	16.73	10.43	28.42	44.42
SmLAC24	19.74	9.96	26.57	43.73
SmLAC25	20.35	12.63	27.54	39.47
SmLAC26	12.74	10.62	29.73	46.90
SmLAC27	18.07	8.94	30.95	42.04
SmLAC28	16.17	11.48	29.57	42.78
SmLAC29	20.32	9.01	27.74	42.93

Supplementary Table 6. Sequence relatedness of SmLACs.

	1	2	3	4	5	6	7	8	9	10	11	12	13	14	15	16	17	18	19	20	21	22	23	24	25	26	27	28
SmLAC1																												
SmLAC2	92.5																											
SmLAC3	44.9	45.4																										
SmLAC4	41.9	42.7	49.4																									
SmLAC5	43.3	43.2	63.9	48.3																								
SmLAC6	54.5	54.3	46.6	45.9	46.7																							
SmLAC7	55.4	54.9	47.0	46.1	48.2	94.6																						
SmLAC8	42.1	42.3	52.3	55.9	49.1	46.6	46.8																					
SmLAC9	76.6	77.3	44.1	42.8	44.1	53.7	54.3	42.4																				
SmLAC10	40.7	40.2	48.8	49.2	45.7	41.3	43.4	47.0	41.2																			
SmLAC11	88.9	89.6	45.9	43.2	43.8	53.4	54.3	43.4	74.0	40.1																		
SmLAC12	61.6	61.4	43.8	42.0	46.3	55.5	56.7	41.0	60.0	42.6	59.7																	
SmLAC13	77.0	78.3	45.5	43.8	45.4	53.9	54.5	43.6	98.2	42.2	74.8	60.8																
SmLAC14	49.2	50.5	46.9	47.0	46.3	53.4	54.8	46.6	50.5	42.1	50.8	47.5	51.9															
SmLAC15	42.6	43.2	47.9	54.2	45.3	47.1	46.4	66.5	42.9	46.8	44.2	41.9	44.5	45.1														
SmLAC16	79.5	80.6	45.3	41.6	43.9	54.3	55.0	42.3	80.3	39.7	78.3	61.4	81.3	50.0	42.1													
SmLAC17	44.6	45.1	93.6	48.9	60.7	45.7	46.3	50.9	44.0	48.1	45.8	44.3	45.3	47.3	47.7	42.3												
SmLAC18	46.6	46.3	68.5	49.9	72.6	48.4	50.2	50.4	47.3	48.2	47.0	46.7	49.3	49.9	49.1	47.0	65.3											
SmLAC19	54.2	55.3	47.0	44.0	48.5	59.2	60.9	44.6	53.6	40.0	54.8	55.3	55.1	56.2	44.7	54.5	46.8	52.0										
SmLAC20	56.8	57.1	47.1	45.0	47.2	63.7	66.7	45.8	54.9	41.7	55.3	55.4	55.3	56.1	44.8	56.1	46.2	51.6	71.8									
SmLAC21	87.3	87.5	45.5	42.7	44.0	56.5	56.0	43.2	74.8	40.0	86.2	61.2	75.4	51.1	43.4	76.5	45.2	46.2	55.5	56.2								
SmLAC22	41.2	42.2	51.0	71.5	48.6	45.4	45.9	56.8	42.3	46.8	41.2	42.0	43.3	44.8	53.9	41.5	50.0	50.8	44.5	45.7	42.0							
SmLAC23	77.7	78.4	45.9	43.8	45.8	54.4	55.0	44.0	96.4	41.8	75.5	60.4	96.9	51.6	44.2	81.0	45.6	49.5	54.8	55.8	75.3	43.0						
SmLAC24	41.5	42.3	47.9	76.9	46.6	44.8	45.9	54.4	41.4	45.0	41.4	41.3	42.3	44.8	53.3	41.0	48.8	50.3	42.2	44.1	41.2	66.2	42.9					
SmLAC25	42.6	44.2	51.0	45.5	49.7	45.2	45.3	47.0	41.9	43.3	43.6	43.9	43.8	46.1	45.9	42.6	51.4	51.6	45.7	44.9	43.3	45.8	43.7	43.9				
SmLAC26	63.8	64.2	47.4	44.9	46.9	59.3	61.0	43.1	63.3	41.0	62.5	63.4	64.3	52.5	42.1	65.1	47.5	49.5	59.4	62.9	63.5	47.1	62.9	44.3	45.8			
SmLAC27	41.2	41.0	50.3	57.6	47.2	45.6	45.7	78.3	41.3	46.0	41.7	40.4	43.0	43.8	64.1	42.4	48.8	49.3	43.6	45.1	42.0	57.6	42.8	54.1	46.5	42.9		
SmLAC28	79.9	80.6	44.9	41.8	43.6	52.7	53.4	42.2	82.5	39.8	77.5	61.9	82.9	49.1	43.1	93.4	44.6	47.3	54.2	55.2	77.3	41.3	82.2	40.6	40.7	64.3	40.9	
SmLAC29	42.7	42.5	56.1	50.3	54.2	45.3	45.6	51.1	41.4	47.2	43.4	44.6	43.0	47.5	48.7	42.8	56.8	55.2	47.0	45.5	43.4	48.9	43.4	49.4	66.4	46.0	49.2	42.0

Supplementary Table 7. Putative regulatory elements in *SmLAC* promoters.

Name	Stress responsive								Hormone responsive						Tissue specific expression			
	Anaerobic induction	Defense and stress	Cold and dehydration	Heat stress	Low temperature	Drought	Wound	Fungal elicitor	ABA	MeJA	GA	SA	Auxin	Ethylene	Meristem	Endosperm	Seed	Shoot
SmLAC1	+	+			+		+	+	+	+		+			+	+		
SmLAC2		+		+				+	+			+			+	+	+	
SmLAC3	+	+		+			+	+	+	+			+			+	+	
SmLAC4		+		+		+				+	+				+	+		
SmLAC5				+	+					+	+	+				+		
SmLAC6		+	+	+			+	+		+	+	+				+	+	+
SmLAC7		+	+	+			+	+		+	+	+				+	+	+
SmLAC8	+			+		+	+			+	+		+			+	+	
SmLAC9		+		+	+				+	+	+	+			+	+		
SmLAC10						+		+			+			+		+	+	
SmLAC11	+	+		+		+	+	+	+			+			+	+		
SmLAC12	+			+					+	+	+	+			+	+		
SmLAC13	+					+			+	+						+		
SmLAC14	+			+	+	+				+	+	+	+		+	+		
SmLAC15	+	+				+			+	+	+		+		+	+		
SmLAC16	+			+					+		+	+						
SmLAC17		+							+									
SmLAC18		+													+	+		
SmLAC19				+			+	+		+	+	+		+	+	+		
SmLAC20		+			+	+			+	+		+		+	+	+		

SmLAC21	+	+	+		+			+	+		+			+	+	+
SmLAC22	+	+		+	+			+		+	+				+	
SmLAC23	+	+	+			+	+	+	+				+	+	+	+
SmLAC24	+				+	+		+	+	+	+				+	
SmLAC25	+	+		+	+			+	+			+		+	+	
SmLAC26	+	+		+	+					+		+	+		+	
SmLAC27	+	+	+	+	+				+	+		+		+	+	
SmLAC28	+	+	+	+	+			+		+				+	+	
SmLAC29		+			+		+	+					+		+	

Supplementary Table 8. The potential target laccase genes of miR397.

miRNA Acc.	Target Acc.	Expectation (E)	Target Accessibility (UPE)	Alignment	Inhibition
ptc-miR397a	SmLAC8.SMil_00009822	1.5	11.672	miRNA 21 GUAGUUGCGACGUGAGUUACU 1<br>::::::::::::::::: ::<br>Target 663 AAUCAACGCUGCACUCAACGA 683	Cleavage
ptc-miR397a	SmLAC24.SMil_00023969	2.0	16.472	miRNA 21 GUAGUUGCGACGUGAGUUACU 1<br>::::::::::::.::::: ::<br>Target 675 AAUCAACGCUGCGCUCAACGA 695	Cleavage
ptc-miR397a	SmLAC4.SMil_00008399	2.5	10.754	miRNA 21 GUAGUUGCGACGUGAGUUACU 1<br>::::::::: :::::::: ::	Cleavage

				Target 684 CAUCAACGCCGCACUCAACGA 704	
ptc-miR397a	SmLAC27.SMil_00026282	3.5	10.001	miRNA 21 GUAGUUGCGACGUGAGUUACU 1<br>:::::::::::.:: :: ::<br>Target 666 AAUCAACGCUGCGCUGAACGA 686	Cleavage
ptc-miR397a	SmLAC5.SMil_00008533	4.0	14.65	miRNA 21 GUAGUUGCGACGUGAGUUACU 1<br>:::::::::::::::::<br>Target 795 AAUCAACGCUGCACUCAACCA 815	Cleavage
ptc-miR397a	SmLAC25.SMil_00024180	4.0	9.34	miRNA 21 GUAGUUGCGACGUGAGUUACU 1<br>:::::::::::::::::<br>Target 672 AAUCAACGCUGCACUCAACAC 692	Cleavage
ptc-miR397a	SmLAC29.SMil_00028534	4.0	6.273	miRNA 21 GUAGUUGCGACGUGAGUUACU 1<br>:::::::::::::::::<br>Target 672 AAUCAACGCUGCACUCAACAA 692	Cleavage
ssl-miR397	SmLAC8.SMil_00009822	1.5	11.672	miRNA 21 GUAGUUGCGACGUGAGUUACU 1<br>::::::::::::::::: ::<br>Target 663 AAUCAACGCUGCACUCAACGA 683	Cleavage
ssl-miR397	SmLAC24.SMil_00023969	2.0	16.472	miRNA 21 GUAGUUGCGACGUGAGUUACU 1<br>:::::::::::.::::: ::	Cleavage

				Target 675 AAUCAACGCUGCGCUCAACGA 695	
ssl-miR397	SmLAC4.SMil_00008399	2.5	10.754	miRNA 21 GUAGUUGCGACGUGAGUUACU 1<br>::::::::: :::::::: ::<br>Target 684 CAUCAACGCCGCACUCAACGA 704	Cleavage
ssl-miR397	SmLAC27.SMil_00026282	3.5	10.001	miRNA 21 GUAGUUGCGACGUGAGUUACU 1<br>:::::::::::.:: :: ::<br>Target 666 AAUCAACGCUGCGCUGAACGA 686	Cleavage
ssl-miR397	SmLAC29.SMil_00028534	4.0	6.273	miRNA 21 GUAGUUGCGACGUGAGUUACU 1<br>:::::::::::::::::<br>Target 672 AAUCAACGCUGCACUCAACAA 692	Cleavage
ssl-miR397	SmLAC5.SMil_00008533	4.0	14.65	miRNA 21 GUAGUUGCGACGUGAGUUACU 1<br>:::::::::::::::::<br>Target 795 AAUCAACGCUGCACUCAACCA 815	Cleavage
ssl-miR397	SmLAC25.SMil_00024180	4.0	9.34	miRNA 21 GUAGUUGCGACGUGAGUUACU 1<br>:::::::::::::::::<br>Target 672 AAUCAACGCUGCACUCAACAC 692	Cleavage

1.2 Supplementary Text

Supplementary Text 1. The coding sequences of *S. miltiorrhiza* laccases.

>SmLAC1.SMil_00001393
atgttttattatcggaagcttaaggttttcatgctgtgctaccttggtattattatggctccagtccatgctttagttcatcataagtttga
agtgagaagatcttcacacaccaggctatgcaccaccaaaagcatgctaacggtaaacgggcagttcccagggccaactatat
atgcaagaaggggagaattggtgatagtcgatgttattaatcgtgccgatgcaaatattactatccattggcatggagtaaaaat
gccgagatatccatggacagatggcgtcgactctgtcacgcagtgtcctattactcccggcaaaagctttcgacaacgagttctg
ctctccgacgaagaaggcactttgttttggcacgcccacatcgattggactcgagctactgtgtatggcgccatcgtcattctacc
gcccaagacacacacttatcctttcccaaagcctcatgctcaacttcccatcttactaggagagtggtggaatgatgatatacaac
aagtttacgaggattttatagcccaaggaatcggtgccaaattgtctgatgctttcctcatgaatggtcaacctggagacctctatc
cctgctcaaaacaagatacattgaagttgagtgtggagccgggcaagacgtacctgatcagaatggttaacgcaatgatgaac
tacattatgtacttcaaaatcaaggaccacaacgtgacggtggtggggacggccggcgcctacacgaagccgctgaacaccg
atcacatcgcgatcgcccctggccagaccatagatttccttctggaagccaaccagccgcccagccgttattacatggccgccag
agtatacgccggccccgtggggtataattacatccccaccacaggaatcgtggagtacgtcggtaactacacccggccctcatc
actgctgcttccatctttccctgagttcgacgatggggtggccaagtccacccatttcagcaacaggctcagaagcctcgccgac
gacaactaccccatcgaagttcctatgaacatcacccacaacatgttcgtcactctctccatcaatacgtggcactgtctcacaaatt
cttgcgtgcacgactacaggctgctggccagcatgaacaacgagaccatgttgttgcccaaaacccaaaatattctcgaggcgt
atttcaaagggatcagaggagttttcacgcccaatttcccagcgatgacaccaataatattcgacttcacgcaaggttataagccg
gagaatgtggcgcccgcccatttcggaacggcggtgtacatgttggactacaactcggaagtcgagatggtgcttcaaggcac
caactatgggactggcgatggaatcgatcatcccgtgcatttacatggatacagcttctacgttgtcggatctggatacgggaac
ttccacccgaacacggatccgccaaactttaatctcattgacccgccgttggtgaacacggttgccgttccaagaagtggatgga
gcgctatcagatttaaggctaacaatccaggagtgtggttgatgcactgccatttcgagcgtcatcacagttggggaatgagga
tggcgttcatcgttacggacggggaaggccccaatgagaagatgttgcctccgcccccagatatgccccgctgtgaagcgccc
cctgcagccttaacaatttag
>SmLAC2.SMil_00001395
atgtttttcataggaagcttaaggttttcatgctgtgctaccttggtattattatggctccagtcgatgctttagttcatcataagtttg
aagtgagaagatcttcacacaccaggctatgcaccaccaaaagcatgctaacagtaaacgggcagttcccagggccaactata
tatgctcgaaggggagaattggtggtagtcgatgttattaatcgtgccgatgcaaatattactatccattggcatggagtaaaaat
gccgagatatccatggacagatggcgtcaactctgtcacgcagtgtcctattactcccggcaaaagctttcgacaacgagttctg
ctctccgacgaagaaggcactttgttttggcacgcccacatcgattggactcgagctactgtgtatggagccatcatcattctacc
gcccaagacacacacttatcctttccctaagcctcatgctgaatttcccatcttactaggagagtggtggaatgatgatgtgcaaca
agtttacgaggattctacagaactaggaatcggtgccaaattgtctgatgctttcctcatgaatggtcaacctggcgacctctatcc
ctgctcaaaacaagatacattgaagttgagtgtggagccgggcaagacttacctgatcagaatggttaatgcaatgatgaacta
cattatgtacttcaaaatcaaggaccacaacgtgaccgtggtggggatggacggcgcctacacgaagccgctgaacaccgatc
acatcgccatcgcccccggccagaccatagatttccttctggaagccaaccagccgcccagccgttattacatggccgccagag
tatacgccggccccgagtggaaaacttacatccacaccacaggaatcgtggagtacgtcggtaactacacccggccctggccg
tcatcaccggtgcttccatctttccctgagatcgacgatggggtggccaggtccacccatttcagcaacaggctcagaagcctcg
ccgacgacaactaccccatcaaagttcctatgaacatcacccacaacatgttcgtcactctctccatcaatacgtggccctgtctcac
aaattcttgcgtccacgtcttcgggctgctggccagcatgaacaacgtgaccatgttgttgcccaaaacccaaaatattctcgagg
cgtattacaaagggatcagaggagttttcacgaccaatttcccagcgatgacaccaataatattcgacttcacgcaaggttataa
gccggggaattttgcgcccgcccatttcggaacggctgtgtacatgttggactacaactcggaagtcgagatggtgcttcaagg
caccaactatggcgttggcgatggaatcgatcatcccatgcatttacatggacacagcttctatgttgtcggatctggatacggga
acttcgacccaaacacggatccgccaaactttaatctcattgacccgccgttggtgaacacggttgccgttccaagaagtggatg
gagcgctatcagatttaaggctaacaatccaggagtgtggttcatgcactgtcatttcgagcgtcatcacagttggggaatgaag

atggcgttcatcgttaaggacggggaaggccccaatgagaagatgttgcctccgcccccagatatgcccctctgtgaaccgccc
cctgcagcctcaacaatttag
>SmLAC3.SMil_00006361
atggagaagaagatgagctctttgtctgcgttggcattgttgctcctttggatgcctctagcaatggccaaactgcacacccatca
gttcgtggttgaagcaaccccagtgaagaggctgtgcaaaacccacaacacgatcacggtgaatgggatgtatccggggcca
acgttggaggtgaacaacggcgacactctggtggtgaaggttattaacagagctcgttacaacgttacaattcactggcatggc
gtgaggcaaatgagaagtgcatgggctgatggcccagaatttgtgactcagtgtccaattagagctggcaaaagttacacttat
cgtttcacaatccaaggacaagaaggcacgctgtggtggcacgcccacagctcgtggctgagagccaccgtctacggcggtc
tcatcattcggcccaaacaaggcgatgcatatcccttccctaagccccaccgtgaaactccccttcttcttggtgaatggtgggac
gccaatcccattgatgttgtgagagaagctagcagaagcggagctgctcccaatgtctccgatgcttacaccatcaacggccaa
cctggtgatctttacaactgctccgccaaacacactcttattgttccaatcaaccccggacaaactaatctactcagagtcgtcaact
ccgcactcaaccaagaactcttcttcaaagtcgccaaccacaagctcaccgtcgtcgccgccgacgcctcctacgtcaagcccttc
tccacctccgtcctcatgctcggccccggccagaccaccgacgtcctcatcaccgccaaccagccacccaacagataccacatcg
cagcacgtgcttacgcaagtgctcagggcgccccttcgacaacaccaccaccaccgccatccttcaataccacaacttctcctcg
ccatcggcaaagcctgtggccgccctcatgcccgctttcaacgacacccccaccgtcaccgccttcaccaggagcttgagaagc
cccaggagggtggaagtgcccaccaagatcgaccacaatctcttcttcacggtggggctgggcctcaacaactgcccgcgcg
gcgccagggcccgaaactgccagggccccaacggaacacgcttcgccgctagcatgaacaatgtgtcgtttgtgctgccctcc
aacttctcactgctgcaggcacatcagcaaggcttacgggggttttcaccaccgattttccggccaggccgcccacgcaattcg
attacaccggcaacgtcagccgctcgctgtggcagcctatgagaggcaccaggttgtataagctgaaatacggggaggccgt
gcaggttgtgttgcagggaacgagcatcttcacggcggagaatcatcccatccatcttcatggctacgatttctacatcgtcgctg
agggattcgggaatttcaatcccaccagagatacgcccaaattcaatctggtggacccacctatgcggaatacggctagtgtac
ctgttggtggttgggccgtcataagatttgttgctgataatccaggagtgtggctgatgcattgccacttggatgtccatatcactt
ggggattagccatggccttcctcgtcgataatggcgtcggcgaattgcaaaccttacagccgcctccaccagatcttcccctttgtt
ga
>SmLAC4.SMil_00008399
atgggtggccctcaattagttgcatgtatagcactgttttgtgcattgatggcgttgtgcctgcttcctcaccatgcaaatgccatca
ccaggcgctacgacttcaatatcaaaatgcaaaatgtaacacgattatgccacacgaagagcatggtgactgtgaacgggaaa
ttcccagggcctcgtgtagtagcaagggagggagatcgcctactcataaaagtcaccaatcatgtccccaacaatatcaccatcc
actggcatggaattaggcaactccggagtggatgggcagatgggccggcgtatatcacacagtgccccatacaaactgccca
aacctatgtctacaatttcacacttgttggccaaagaggaaccctttttggcatgcacatatttcctggcttagagctactctttacg
gtcctatcatcatcctccccaagataaatgttgattatccattccagaaaccctacaaggaactccccatcatatttggagaatggtt
caatgccgacccggaagccatcatcaagcaagctctagccagcggcggcggccccaacgtctccgacgcctacaccatcaac
ggccttccgggcccactctacaactgttctgccaaagatacattcaaattgaaggtgcaggcaggcaagacgtacctcctccgc
atcatcaacgccgcactcaacgacgagctcttcttcagcatcgccaaccacactctcacggtggtcgacgtcgacgcagtctacg
tcaagcccttccagaccaccaccatcctcatcacaccgggccagaccaccaacgtgcttctccacaccaaaccacgcttccccgcc
gccacattcctcatgaccgcccgcccctacgtcaccggccagggcaccttcgacaactccaccgtcgccggaatcctcgagtac
gaggcaccatcccttcacataaaaaacctccctctctacaagcccctcctcccgcctctcaacgacacctcattcgccgcccgcttc
gcggggaagctccgcagcctggcgagcgcggagttccccgcgaaggtgccgcggagggtggagaggcgcttcttcttcac
cgtgggattgggcacgagcccgtgcgacaggccggacgccacgtgtcagggcccgaacgggaccaaattcgcggcgtccg
ttagcaacgtgagcttcgtgcagcccacgacggccctcctggaggcccacttcacgggccggtcgcggggcgtgtacagccc
ggattttccggtcagcccgctccgctggttcaactacaccggcaccccaccgagcaacacggcggtcgggaacggcacgaag
ctggtggttttgccgttcaacgcgagtgtggaggtggtgatgcaggatactagtattcttggagccgagagccatcccctccatc
tccatggattcaatttttttgtggttggacagggtttcggaaactacgacccggttaatgacccgaatagcttcaatctggtcgacc
cagttgagaggaatacggttggtgtgccggcgggtggatgggttgccattcgtttttcgctgataatccaggtgtatggttcat

gcattgccatcttgaagttcacacgagttggggtttgaagatggcatggcttgttttggatgggaagcttccaaatcaaaagctcc
ttcctccaccgtccgatcttcccaaatgctga
>SmLAC5.SMil_00008533
atgtgtgatggaccggggaaagtcttcaagatactcgaatcttctgtgtcagcaatgaaggatgcggaggttggtgataggatt
ctcgaggcgctgcattcttgggatcctgccgaagttgtcgaagaaacgccggtgaataggctgtgcaaaactcgcaagataat
cacggtgaacggacagttcccggggccaaccattgccgcaagaaacggagactcactagccatcagagttaccaatcgagcc
cgttacaacgttaccatccactggcacggaatacgacagatgcggaccccatgggccgatgggcccgaattcgtgacgcaatg
ccccatcaggcccggcggaacctacacttaccgtttcaagatagagaaccaagagggcacattgtggtggcacgcccacagtc
ggtggctccgggccaccgtctacggcgccctcgttatttttccgaactcgagctcctcctatcccttctctgcacccaagattgatgt
tcctatcattcttgagtcaatcgataactatagcgatgatatctttattattatacatttgaaacgacaaataggtgagtggtggaata
gaaacatccttaaagttatgagacaagccctcttcaccggagctgccccaaatgtttcaaacgcctacacggtcaacggtcagcc
aggagatctctacaaatgctctagccaagttggccctctaaataaaaatcctggctccgtcactgtcaggcacaccaaaatctcag
tgaaccagggagacacggtgatgctaagagtaatcaacgctgcactcaaccaagagctcttcttcagcgtggccaaccacaaa
ctgacggtggtcggagccgacgccgcctacaacaggcccttcaccaccaacgtgatcatgaccacgaacgtcctcctcacggc
cgaccagccacccgcgcgctactacatggccgcgcgtgcctaccagtccgcacagaacgcggccttcgacaacaccaccacc
accgccatcctcgagtacaaaaccgccgccgccgccaaccccatcctgccccgactaccggccttcaacgacacggccacggt
caccgccttcacgagccagctcagagtcatacgagcccaagccaaagtcccgactcaactcgatgaaaatctattcttcgccttc
ggattaggcctcatcaactgcaatcccggccctcgatgccaagggcctaacaacacgcgctttgccgctagcgttaacaatgtat
cctttgtgctgccgagaaccgtttctcttctgcaagcctactaccaaaacataccgggagtttacaccaccgatttccctccttctccg
ccgcgcaagttcgactacacgggcaacgtgagccgggcgctgtggcagcccggctccggcacgaaaatgtacaagctcaag
ttcgggtcgaacgtgcagatcgtgttgcaggacacggccatcgtgacgaccgaggaccaccccatccatctacacggctacca
cttcttcgtcgtcggccaaggcttcgggaacttcaatccggccaccgacacggctaccttcaatctcgttgatccgccggttcgga
ataccatcaacgttccggtcggagggtgggccgtcatccgcttccacgccgacaatccagattcacatcttacttggggtttagc
gatggcattcatcgtcggaaatggagaagggccgtcgcaaaccgtcgagccgccgccggctgatctcccacaatgctga
>SmLAC6.SMil_00009265
atgaagtttctcatcgtgtatttttcgttgctttttttcatatgtggctttgtagcagttcatggaaagatcctaagacagagatttgtgt
tacaagatgccccatacacacgattgtgcggcagtaagagcatattaaccgtgaacggaaaatttcccgggcccaccatatatg
cgagagtgggagacacagtaattgtggacgtcatcaacagagctagtgaaaatattactattcactggcatggggtgaaacag
ccaagaaacccatggtcagatgggccggcttacataacccagtgcccgatccgaccgggaacgaatttcagccaaagaatcgt
gttgtctgatgaaatcgggactctatggtggcacgctcacatcgaatggtttcgcgctactgtctatggtgctctcgttgttttacct
aacttgggagatcaatatcccttccctaaacctgctgccgaaattcccatcatattaggagaatggtggaacagtgatataaggg
aagttctgagggaaggccttcaaagcggatcggattttaatgcatctaatgctttcctcatcaatggtcaacctggagatctatatc
cttgttccaattcagatacattcaaagtgaaagtggactctggcaaaacatacctacttcgaatgatcaacaatgtgatgaacaac
atcatgttcttcaaaatcgccaaccacaacctcaccgtagtcggctccgacgccgcctacacgaagccgttcacggccgagtacg
tggcaatatctccgggccaaaccatagatgtcttgttcgtagccaaccaacaaccaagccactactacatggcctctcggcccca
caacgtcgccgggaacttcgccaacacgaccacaaccgccatcctcgagtacaccggaaactacacggcaccctctggacaa
ggagcttctgtttaccctctcggtaaacatattcgaatgcgctctaaacgagacttgcaacggaccgttgcaaggccggttcaga
acgagcctcaacaacataacttcgtgcagccaagaatctccattcttgatgcctattataacggtgttaacggagtctacgaggaa
gatttccctcagaatccgcctttcccgttcaactacacgatggagttcgtgccacagctgctatggatacctcaaaatgggacaga
agttagagtggtggagtataatacctcagtagagcttgtcttccaaggaacaaacattcttggtgggacataccatcctatgcatc
tccatgggtacagcttctatgtggtcggaagaggcaacggaaactttgaccgggaaagggacccttccagctataatcttgtcg
atcctcctctgcagaacaccattgcagttccaagaaacgggtggacagcgattaggttcaaggcgaataatccaggtgtttggc
ttctgcattgccatctggagcgccacgtgacctggggaatggagatggcgttcatcgtcagagagggaaagagccaagacgc
taagatgctgcctccacctgcagacatgcccagatgctaa

>SmLAC7.SMil_00009266
atgaagtttctcatcgtgtatttttcgttgcttttttcatatgtggctttgtagcagttcatggaaagatcctaagacagagatttgtgt
tacaagatgccccatacacacgattgtgcggcagtaagagcatattaaccgtgaacggaaaatttcccgggcccaccatatatg
cgagagtgggagacacagtaattgtggacgtcatcaacagagctagtgaaaatattactattcactggcatggggtgaaacag
ccaagaaacccatggtcagatgggccggcttacataacccagtgcccgatccgaccgggaactaatttcagccaaagaatcgt
gttgtctgatgaaatcgggactctatggtggcacgctcacatcgaatggtttcgcgctactgtctatggtgctctcgttgttttacct
aacttgggagatcaatatcccttccctaaacctgctgccgaaattcccatcatattaggagaatggtggaacagtgatattaggg
aagttctgagggaaggccttcaaagcggatcggattttaatgcatctaatgctttcctcatcaatggtcaacctggagatctatatc
cttgttccaactcagatacattcaaagtgaaagtggactctggcaaaacatacctacttcgaatgatcaacaatgtgatgaacaac
atcatgttcttcaaaatcgccaaccacaacctcaccgtagtcggctccgacgccgcctacacgaagccgttcacggccgagtacg
tggcaatatctccgggccaaaccatagatgtcttgttcgtagccaaccaacaaccaagccactattacatggcctctcggccccac
aacgtcgccgggaacttcgccaacacgaccacaaccgccatcctcgagtacaccggaaactacacggcaccctcgtctcctctc
ctccccaccctccccgactttgcagacgaggtcgcctctgacagtttcacagcacggctgcgaagcttagcgacagaggctcac
cccattgacgtccctcaaacagtggacaaggagcttctgtttaccctctcggtaaacatattcgaatgcgctctaaacgagacttg
caacggaccgttgcaaggccggttcagaacgagcctcaacaacataacgttcgtgcagccaagaatctccattcttgatgcctat
tataaccgtgttaacggagtctacgaggaagatttccctcagaatccgcctttcccgttcaactacacgatggagttcgtgccaca
gctgctatggatacctcaaaatgggacagaagttagagtggtggagtataatacttcagtagagcttgtcttccaaggaacaaa
cattcttggtgggacataccatcctatgcatctccatggctacagcttctatgtggtcggaagaggcaacggaaactttgaccgg
gaaagggacccttccagctataatcttgtcgatcctcctctgcagaacaccattgcagttccaagaaacgggtggacagcgatta
ggttcaaggcgaataatccaggtgtttggcttctgcattgccatctggagcgccacgtgacctggggaatggagatggcgttca
tcgtcagagagggaaagagccaagacgctaagatgctgcctccacctgcagacatgcccagatgctaa
>SmLAC8.SMil_00009822
atggaagtctgggttcgtgttttgatcctcgtagcatgcatgtatccccttatggttgacggcgctgttcgccgttaccagttcaatg
tgatgatgaagaacacatctcgcctatgttcttccaaatccatcgtcaccgtcaacggaaaattccccggcccgacactctacgcg
agggaagacgacaccgtcctcgtccgagtcatcaaccgagtccagtacaacgtttccatccactggcatggaattaggcaactc
ggaacaggctgggccgacgggccggcatacatcacgcaatgcccgatccggcccggtcacagctacgtgtacaaattcacag
tcaccgggcaacgaggcacacttttgtggcacgcccacattctatggctgagggccaccgttcatggcgccatcgtcatcttgcc
taaacgaggggtcccctacccccttccctaagcccgatcacgagcacgtcgtcgttttagctgaatggtggaaatccgacactgaa
gccgtgatcaacgaagccatgaaatcgggcgtggcccccaacgtgtccgatgcacacaccatcaacggccatccgggcccac
tctcaaattgccccacaacaggtggtttctcactaaatgtgtcaccgggaaaaacctacatgctccgcttaatcaacgctgcactca
acgaagagctcttcttcaaaatcgccggccacaaactgacggtggtcgaggttgatgccacctacgtgaagccgttcaaaaccg
acaccgtcctcatcgcccccggccagaccacgaacgtcatcgtctcggccgatcagggctcaggcaagtacatggtggcggcc
tcgccgttcatggacaccatgatcgtggcggtggacaacatgacggccaccgccaccctgcactactccggcgcccttgccaac
tccccgactaccttcacccgaccgccggctcggaacgccacccccgtcgccaactccttcaccgactccctccggagcctaaact
ccaacaaattcccggccaacgtgccgcaaaagatcgaccactccctgctcttcgccgtcgggctcgggctcaacccgtgcccga
cgtgcaaggcgggcaacggcagcagagtcgtcgcgagcatcaacaacgtgacgttcgtgatgcccacgatggctctgctgca
ggcgcacgtcttcaacatcaagggggtcttcaccgccgacttcccgggaaaccctccgacgccgttcaactacaccggcgcgc
cgccagcgaatatggcgacgacgagcgggacgaaggtgtataggctcgcgtacaactcgactgttgaagttgtgttgcagga
taccggcatcatcgcccctgaaaaccaccctgttcatctgcatggattcaatttcttcgcggtagggaagggattgggcaatttca
atcccaaaaccgatccacagaaattcaatctcgttgatcctgttgagaggaacacaatcggagttccttctggtgggtgggtcgc
tattagattccgagccgataatccaggggtgtggtttatgcattgccatttggaggtgcacacgacatgggggctaaaaatggc
atttttggtggacaatggaaagggcccaaatcagtctattttgccgccacctaaagatttgccaaaatgttga
>SmLAC9.SMil_00011367
atggtttcttataggaagcttaaggttttcatcatgtggtacattggtattattatgtttttgctcggaggcatgggtccagtccatgct

ttagttcataagtttgaagtgagaagatcttcacacaccaggctatgcaccaccaaaagcatcctaacgataaacgggcagttcc
cagggccaactatatatgctagaaggggagaattggtggtaatcgatgttattaatcgcgccgatcaaaatataagtatccattg
gcatggagtaaaaatgccgagatatccatggacagatggcgcgagccatgtgacgcagtgtcctattagtcccggcaagagg
tttagacaacggatgttgctctccaacgaagaaggcactttattttggcacgcccacagcgattggtctcgagctactgtgtatgg
cgccatcatcattctaccgcccaagacacacacttatcctttccctaagcctcatgctgaagtgcccatcttactaggagagtggtg
gaatgccgatgtgcaagaagtttacgaggattctatagcccaaggaatcgatgccaatttttctgatgctttcctcatgaatggtca
acctggcgacatgtacccctgctcaaaacaagatacattcaagttgagtgtggagccgggcaagacttacctgatcagaatgat
caacgcaatgttgagctacattatgtacttcaaaatcaaggaccacaacgtgacggtggtgggcatggacggcgcctacacga
aaccgctgaacaccgatcacattgcgattgcccccggccagaccatagatttccttctggaagccaaccagccgcccagccgtta
ttacatggccacaaaaaatatacgccagcccctggtggcatacttacgtccccaccaccggaatccttgagtacgttggtaactaca
tcccccccctcatcactggagtttccatatttccctaaattcggtggctatggctgggccgagtccatccatttcagcagcaagctca
gaagcctcgccaacgacaagcaccccatcaaagttcctatgaacatcacccacaacctcttcttcactctcaccatcaatatgtggc
cctgtgagacaaattcttgcgagaggaacaacagggtgctggccagcatgaacaacatctccatgctgtccccgctaacccaaa
atattcttgaggcctattacaaagggatcagaggagttttcacgcccgatttcccggcgaagccagcaagaatattcaacttcac
gcaaggtcataagtcattgtatgaggggcgcaccgaattcggaacggctgtgtatatgttggactataactcagaagtggagat
cgtgtttcaaggcaccaactttggcgagggaagcgatcatcccatgcatttacatggatacagcttctacgttgtcggagctgga
tacggggacttcgatccgaacagggatccgcaaaactttaatctcattgacccgctcttgatgaacaccgttaccgttccaagaaa
tggatggagcgctatcagatttaaggctaacaatccaggagtgtggtacatgcactgccatttcgatcgtcatcagacatgggg
aatgaagatggtgttcatcgttaaggacggggaagcccccaatgagaagatgttgcctccgcccccagatatgccccgctgtca
accgcccccctcaacagttattatttcggatttaa
>SmLAC10.SMil_00012308
atggcagtcacaaccactgccctcgtgtgtgcattgttgagtttgctttccatctctcatagcgttcatggaatcaagagatggcct
ggcggggcttccaccagattctacgacttcaaggtgcaaacgaagagggtgacgaagttgtgcaacagcaaggagatcgtg
accattaatgagatgtaccccggtccagtggtttacgctcaagaagacgatcgtctcatcatccgagtctccaatttgacgcctcac
aatgccacaattcactggcatggggtccgacaacggttgtcgtgctggtccgacggcccatcttacgtgacacaatgtccgatcc
aaccggggcagactttcagttacgagttcaccctggttcaacaaaagggcaccttttctggcatgctcatgtctcgtggctccgg
gctacggtttatggtgccttgatcgtttaccccagacccggtgtaccctaccctttcccttaccccctaccaggagcatattgttattcta
ggggaatattggttgagagatcctctaaagatcgagcaggcagttttagctagcggcggaggcgctcccatcgcggatgctta
cacgatcaacggccaccccggccccaactataactgctccgtcaacggtaagcctcaagtcatgaacgtggtacccgggaaga
cctacctgctgaggttgatcaacgcgggattgaacacagaaaacttctttgcaattgctaatcacaagctcaccatagtcgaagc
agatgcagagtacacaaaggcgctccacaccgacaaggtgatgctcgcgccgggccagaccctcaacgtcctcgtcacagca
gatcagcccataagtagatactccatggctgtcgggccatacatgtctgcaagaaacgtaccgtttcagaacataacctcagtag
gatattttcagtacctaggtgcaacaccaaacagcatatctttaccagccatgctacctagtttcaacgacaatcttgccgttaaaac
tgtaatggacgggctccggggcctcaacacctcggatgttcctaaagtaattgacaagaacttgtttgtcaccataggtgtgaat
gtgaataagtgtaatagaaaaaaccccaagaaaaactgccaaggcactaatggtggggtgtttgctgcttccatgaacaatata
agtttcattagacctaatgtttcacttttagaagcttattacagggataaagctggctacttcactgatgatttccccggggcacccctt
aaaattttacgattttgttaacggggcgcctaacaatgctcctaatgacaccaacgcgatgaatgggacgaggttgtttgtgcttg
agtatgggacaagggttcaactcattttgcaggatactggcactgtctcgactgagaatcatccaattcatcttcatggctacagtt
tctacgtcgtggggtatggcacgggtaactacaatccggatacggctaatttcaacttgatggatccgccttacatgaacaccatt
ggagttcctgttggtggatgggcagcaattagatttacagctgataatccaggggtctggtttatgcattgccatctggagataca
tttatcatggggtttgtctgtggcaattattgtcaagaatgggcaaggaccactcgagaccctcccccaccctccagctgattaccc
tcggtgctag
>SmLAC11.SMil_00013111
atgttttttcataaggttttcatgctgtgctaccttggtattattatggctccagtccatgctgtagttcatcataagtttgaagtgagaa

gaacttcacacacaaggctatgcaccaccaaaagcatgctaacggtaaacgggcagttcccagggccaactatatatgctcga
aggggagaattggtggtagtcgatgttattaatcgtgccgatgcaaatattactatccattggcatggagtaaaaatgccgagat
atccatggacagatggcgtcgacgctgtcacgcagtgtcctattactcccggcaaaagctttcgacaacgagttctgctctccga
cgaagaaggcactttgttttggcatgcccacatcgattgggctcgagctactgtgtatggcgccatcgtcattctaccgcccaaga
cacacacttatcctttccctaagcctcatgctcaacttcccatcttactaggagagtggtggaatgctgatgtgcaacaagtttacga
ggattttatagccggaggagcgagtgcccaattgtctgatgctttcctcatgaatggtcaacctggcgaccagtatccctgctcag
aacaagatgcattgaagttgagtgtgcagccgggcaagacttacctgatcagaatggttaacgcaatgatgaacttcattatgta
cttcaaaatcaaggaccacaacttgacggtggtggggacagacggcgcctacacgaagccgctgaacaccgatcacatcgcg
atcgcccccggccaaaccatagatttccttctggaagccaaccagccgcccggccgttattacatggccgccagagtatacgcc
ggccctgtggggtataattacatccccaccacaggaatcatggactacgtcggtaactacacccggccccgcccctcatcaccg
gtgcttccatctttccctgagatcgacgatggggccaggtccacccatttcagcaacaagctcagaagcctcgccgacgacaact
acccaatcaaagttcctatgaacatcacccacaacatgttcgtcactctctccatcaatacgtggccctgtcccacaaattcttgcgt
gttcggctacatgctgcgggccagcatgaacaacgtgaccatgtcgttacccgaaacccaaaatattcttgaggcctattacaaa
gggatcagaggagttttcacgcccaatttcccagcgatgacaccaataatattcgacttcacggaaggttataggcgggggaat
gtggcgcccgcccaatccggaacggctgtgtacatgttggactacaactcggaagtcgagatggtgcttcaaggcaccgacta
tggcgttggagatggagtcgatcatcccatgcatttacatggacacagcttctacgttgtcggatctggatacgggaacttcgac
ccaaacacggatccgccaaactttaatctcattgacccgccgttggggaacatggttgccgttccaaaaaaatggatggagcgct
atcagatttaaggctaacaatccaggagtgtggttcatgcactgtcattatgagcgtcatcacagttggggaatgaagatggcgt
tcatcgttagggacggggaaggccccaatgagaagatgttgcctccgcccccagatatgcccctctgtgaaccgccccataca
gcctcaacaatttag
>SmLAC12.SMil_00017786
atgttttcggctacaaaggccttcattctgtgctacttaggtgttattatgtttggaggcgcaacgcaagttaatgcttcaattcgttg
ttgtacgtttgaagtggtaaattctgcacacactagattatgcaccaaaaagactatgctgaccataaatggacagttcccgggg
ccaaccatatatgctaggaggggagatctgattacaatgaatattgtgaatcgcgccgatcaaaatatcagcatccactggcatg
gagtaaaaatgccccagtatccatggtcagatggcaccaactatgtgacgcagtgcccaattgaacctggacaaaagtttaccc
aacaaattattctctccgacgaggaaggcacattgtggtggcacgctcacagcgattgggctcggaattctgtgtatggcgccat
tgtcattctaccgcccaggaccgagtcttttcctttccccatccctcatgcccattttccccccatcctaataggagagtggtggaata
ccgagattgaaacagtcttcaaggagtttctcgagactggtggagatcctgaagtttctaatgctttcctcttcaatggccaaccag
gcgacttgtatccatgctcaaaccaagatacgtacaagttgcgagtggaatatgggaaaacttacttgatcaaattggttaatgc
ggcgctggacaacatcatgttcttcaaaattgccaaccacgagctcaccgtggttggcgctgacggcgcctacacgaagccgtt
gacgaccgattacgttgcgattccccccggccagaccctcgacttgctgctggaggctaaccagccacccagccattattacatg
gccaccagaatgtacaccagcgaagacgtccacagccccaccaccccccaccacagcaatcatcgagtacgtggggaactaca
cgccgccgccatcaccacttcttccaagtttacccgatgtcaaagactcagcggcggtcaacaacttcatcaaccgactgagaag
tctaggcaacgacgtggatgttcctaagaacgtggaggaattcatgtactacactctatcgatgaatctaatgccgtgtctgaaaa
gcacgtgttcggggtcgactaggtttctggcaagcgtgaacaacgtgtcgttcgtgcttccccgactccgactggacattctaca
ggcttattacagagggatcaatggagtttacacagaggatttcccagacttcccttatttatcttacgacttcaccggcgttgtgcta
cccgaagaagaggcggggacgaacttcggaacaggggttcggatgctggagtataacaccacggtggaagtggtgcttca
aggagccaaccatctcatcgatcatcccatacatctacacggatacaggttctacgtggtcggatacggtttagggaactacaac
agaatcagggatgtggccaactacaatctggtcgacccgccgttgatgcaaaccattgtagtgcccaaaaacggatgggccgc
aataagatttaaggcaaacaatccgggagtgtggtacatgcattgccatatcgagcgtcatatcagctggggaatgggaatgg
tgttcatcgtcaaagatggggaacgtccccatgagaagatgttgccacctccgccggatatgccacgttgctga
>SmLAC13.SMil_00019237
atggtttcttataggaagcttaaggttttcatcatgtggtacattggtattattatgtttttgctcggaggcatgggtccagtccatgct
ttagttcataagtttgaagtgagaagatcttcacacaccaggctatgcaccaccaaaagcatcctaacgataaacgggcagttcc

cagggccaactatatatgctagaaggggagaattggtggtaatcgatgttattaatcacgccgatcaaaatataagtatccattg
gcatggagtaaaaatgccgagatatccatggacagatggcgcgagccatgtgacgcagtgtcctattagtcccggcaagagg
tttagacaacggatgttgctctccaacgaagaaggcactttattttggcacgcccacagcgattggtctcgagctactgtgtatgg
cgccatcatcattctaccgcccaagacacacacttatcctttccctaagcctcatgctgaagtgcccatcttactaggagagtggtg
gaacgctgatgtgcaacaagtttacgaggatgctatagcccaaggaatcgatgccaatttttctgatgctttcctcatgaatggtc
aacctggcgacatgtacccctgctcaaaacaagatacattcaagttgagtgtggagccgggcaagacttacctgatcagaatga
tcaacgcaatgttgagctacattatgtacttcaaaatcaaggaccacaacgtgacggtggtgggcatggacggcgcctacacg
aagccgctgaacaccgatcacattgcgattgcccccggccagaccatagatttccttctggaagccaaccagccgcccagccgt
tattacatggccaccaaagtatacgccagcccctggtggcatacttacgtccccaccaccggaatcctcgacagcaagctcaga
agcctggccaacgacaagcaccccatcaaagttcctatgaacatcacccacaacctcttcttcactctcaccatcaatatgtggccc
tgtgagacaaattcttgcgcgaggaacaacagggtgctggccagcatgaacaacatctccatgctgtccccgcaaaccctaaat
attcttgaggcctattacaaagggatcggaggagttttcacgaccgatttcccggcgaagccagcaagaatattcaacttcacgc
aaggtcataagtcattgtatgaggggcgcaccgaattcggaacggctgtgtatatgttggactataactcggaagtggagatc
gtgtttcaaggcaccaactttggcgagggaagcgatcatcccatgcatttacatggatacagcttctacgttgtcggagctggat
acggggacttcgacccaaacagggatccgcaaaactttaatctcatcgacccgctcttgatgaacactgttaccgttccaagaaa
tggatggagcgctatcagatttaaggctaacaatccaggagtgtggtacatgcactgccatttcgatcgtcatcagacttgggga
atgaagatggtgttcatcgttaaggacggggaagcccccaatgagaagatgttgcctccgcccccagatatgccccgctgtcaa
ccgccccctcaacaattattatttaggatttaa
>SmLAC14.SMil_00020653
atgcttctgaaatatcactgcaatattttactaatcttaatcttcgcaaactccatctgcgtaaaagcatccgacattcgacgctacag
cttcgttgttagggaagctttatacgagagattatgtgagagaaagtggattttgacagtgaacgggaaattcccaggcccaag
cctcaaggttcatgcaggggagaccatagttgtggacgttctgaacaggggaaattacaacattaccctccactggcacggagt
taagcagcctaggaatccgtgggcagacggccccgcatatatcacccagtgtccgatccagccgggcaagcgcttcagtgag
aagatcatattttccaaggaagaagggacgctgtggtggcacgcccacagcgactggtcgcgcgccacggtccacggcctca
tcatcgtgtacccgaggcccggaggctcgtacccattcccgaagcccgatgcagaggttcccatcattctaggtgagtggtgga
aagaggatgtgatgaaggtgttggaggagtttgttgcttcgggaggccagccaagggactccgatgcctataccataaatggc
cagcctggggagctctacccttgctccgcgcctcgtcgcttcgtcatgaaagttgaccccggcaagacctacctcctgcgcatag
tcaacgcggacatgaacgagatcctcttcttcggggtggcgcgccaccacctcaccgtcgtcggcaccgacggctgctacacg
gagccgctcacgagaggctacatcgccatcagccccggccagaccatggactgcctcctccgctccgaccaaccaccggccc
gctactacatggctgcctgcccctacgtcaacggcgccggaatcagcttcgacaacaccaccgccaccgccatcctccgctacg
cgggatcatcccgccacaccccacccgaattccccaaactaccctcccccaacgacacctccgccgccctaaacttcacctacag
catcaaaagcctaatctcgaagctccacccaatcgtagtccctcaaaagatcaacaagcgcatcaccaccaccgtctccgtcaac
gtcttcccatgcgaggcctgcgccggccccaacgggacccgcctggccgccgccatgaacaacatcagcttctcggccccgcc
ggtcgacatcctcgaggcctactattaccaaatcccgggcatttttagcaagaaattcccggaatctccaccattggtgttcaatta
cacagccgatttcatccccttggagctggaaatccctaaaaagggcactaaagtcgcaatgctgaggtacaactccaacatcga
ggtagttttccaaggtacaaaccaagtgggtgggctggatcaccccatgcatctgcatggattcagtttcttcgttgttggttggg
gatttggcaactttgatcccaacaaagatcccttgagctataatctcgtcgacccgcagaagcgtaacaccgtcatcgtccccaga
aatgggtggacggccatcaggttcactgccgacaatccaggggtgtggttcttgcattgccatttcgagcggcacctgacgtgg
gggatggagacggtgttcatcgtgaggagcggaagagggccggctgcgcgtgtgctcccaccgccgccagatatgccgcc
gtgttga
>SmLAC15.SMil_00020657
atggggggggaaagttggcgtcgctctagctctcatgctcctcacactcttctcctcttcgtcgcgtgttgactcgagaatccgacac
tataagttcacggtggtgaggaagaacacgacgaggctgtgtgcgagcaagcccatcatcacggtgaacggaaaattcccgg
ggcccactctctatgcacgggaagatgatacagtcattattagagttgtgaaccatgtcacagacaatgtcacaatacactggca

cggagttcggcagctccggacggggtgggccgacgggccggcttatatcacgcagtgcccgatccaaacaggtcagagcta
catttacaagtttaccctgacggggcagagagggactctgctttggcatgcgcatatcaactggcagagagccacggtgcacg
gcgccattgtgatcctgccgaggctcggagttccgtatccatttcccgagcctcacaaagaagagatcatcatactaggagagt
ggtggaaggctgatgtggaagctgtgataaaccaagccatgcaagcaggcctaccgccaaacgtatccgatgcacacaccat
caatggccttcccggcccttctccaaattgctcttacgcagggtatagtctccatgtagagagcgggaaaacataccttctacgca
ttataaacgcagcagtaaacgaagagctcttcttcaagatcgccgggcaccacctcaccgtcgtcgaagtcgacgccgcctata
cgaagcccttcaaaaccgacaccgtattcatcgggccgggccagaccaccacggccctcctcgccgccgaccggcccgccgg
caactacatcatcgccatctccccttcatggacaccatcgtcgccaccgacaaccagatcgccaccgccaccctccgctacgac
ggcgccgcccctccgccgccgccgccgtcgccgccgacgtccccgcccgcaacgcgacggcggtcacggccgcgttcatg
gacggcctccggagcctcagcccggcggcggcgccgtcccggccggcgacgtcgaccactcgctcctcttcgccatcggcgt
cggcgtcaacccgcgcggtactacaacgtgagcggggtgtacacggcggactttccgcggcgccgccggcgccgtacgact
acaccggggcggcgccggcgaacatgaggacgacgacggggacgagggtttaccgagtggagtacaacgcgacggtgc
agatcgtgctgcaggggacggcgatgatagcgccggagagccacccgacgcatctgcacggattcaatttctacgccgtggg
gagaggagttgggaattatgatccgattaatgatccaaagaggtttaatcttgatgatcctgttgagaggaacaccataagtgtg
cctaccggtggatggactgctataaggtttagggctgataatccagggatgtggtttttgcattgccatttggaagtgcacacaa
catgggggcttaagatggcatttttggtggaaaatgggaaaggcccaaatcagtcaatcttgcctcctccaaaagatcttccaaa
atgttga
>SmLAC16.SMiI_00021274
atgcaggaacaagattttcgaagatggtttgttgctgttagaggtttggagattggggagtccgaatttggtgctccgttgtgga
gttggtctggtgggagtttattttcttgtgccatttcccttgttttttcacttatgttctgtattcttggtacctctggaagcttaaggtttt
catcctgtgctaccttggtattattgtgctcgctccagtgcatgctttagttcataaatttgaagtgagaagatcttcacacacaagg
ctatgcagcaccaaaagcatgctaacagtaaacgggcagttcccagggccaactatatatgctagaaggggagaattggtggt
aatcgatgttattaatcgcgccgatgcgaatataagtatccattggcatggagtcaaaatgccgagatatccatggacagacgg
cgtcacccatgtgacgcagtgtcctattagtcccggcaagaggtttagacaacggatgttgctctccaacgaagaaggcacttta
ttttggcacgcccacatcgattggtctcgagctactgtgtatggcgccatcatcatcctaccgcccaagggacacacttatcctttcc
ctaagcctcatgctgaacttcccatcttactaggagagtggtggaatgctgatgtgcaacaagttttcgagaagtctataaacgaa
ggagtagctgccgatttttctgatgctttcctcatgaatggtcaacctggcgacctgtatccctgctcaaaacaagatacattgaag
ttgagtgtggagccgggcaagacttacctgatcagaatggttaacgcgatgatgaacttcattatgtactacaaaatcaaggacc
acaacgtgacggtggtgggcatggacggcgcctacacgaagccgctgaacaccgatcacatcgcgatcgcccccggccaga
ccatagatttccttctggaagccaaccagccgcccggccgttattacatggccgccagagtatgggccagcccctattggcatac
ttacatccccaccacaggaatcgtcgagtacgtcggtaactacacccggccttcgtcaccggcgcttccctctttcccgggattcca
aggctgggcggagtccacccatttcagcaagaggctcagaagcctcgccaacgacaactatcccatcaaacttcctatgaacat
cacacaccacctcttcttcgctctctccatcaatgtggggccctgtttctcaaattcttgcaagaatggcgacaggctgctggccag
catcaacaacatctccatgctgtacccggaaaccctaaatattcttgaggcctattacaaagggatcggaggagttttcacgacc
gatttcccggccaagccgtcaacaatattcgacttcacgcaaggctataagccagaggataggggcatcggaatcgatcatccc
atacatttacatggatacaacttctacgtgatcggaactggattcgggaacttcgacccaaccacggatccgccaaactataatct
cgttgacccgccgttgatggacacggttgccgttccgaaaaatggatggagcactatcagatttaaggctaacaatccaggagt
gtggtacatgcactgtcatttcgaacgtcattacacttggggaatgaagatggtgttcatcgttagagacggggaaggccccaa
tgagaagatgttgcctccgccccttgatatgccgcgctgtgaaacgccaaatcaacaattattgtttaggatttaa
>SmLAC17.SMiI_00021810
atgggggaaaatggggttttaggtctcaaaatcgcgactggggcccacttgggggaagccccgccttcttccggcggtccc
ggtccgtgtccgcggccttcaaacgtaggcaaggatcaggggacccgcggccgctggtggtgaccgcggataatatttacaa
acttgcaaaactcagttttgctttcagcatccctacctccaaaagaaaagaaataaagcagctagaaattaatggagaagaagat
gagctctttgtctgcgttggcattgttgctcctttggatgcctctagcaatggcgaaactgcacacccatcagttcgtgcaacccca

gtgaagaggctgtgcaaaacccacaacacgatcacggtgaatgggatgtatccggggccaacgttggaggtgaacaacggc
gacactctggtggtgaaggttattaacagagctcgttacaacgttacaattcactggcatggcgtgaggcaaatgagaagtgca
tgggctgatggcccagaatttgtgactcagtgtccaattagagctggcaaaagttacacttatcgtttcacaatccaaggacaag
aaggcacgctgtggtggcacgcccacagctcgtggctgagagccaccgtctacggcggtctcatcattcggcccaaacaagg
cgatgcatatcccttccctaagccccaccgtgaaactccccttcttcttggtgaatggtgggacgccaatcccattgatgttgtgag
agaagctagcagaagcggagctgctcccaatgtctccgatgcttacaccatcaacggccaacctggtgatctttacaactgctcc
gccaaacacactcttattgttccaatcaaccccggacaaactaatctgctcagagtcgtcaactccgcactcaaccaagaactcttc
tccaaagtcgccaaccacaagctcaccgtcgtcgccgccgacgcctcctacgtcaagcccttctccacctccgtcctcatgctcgg
ccccggccagaccaccgacgtcctcatcaccgccaaccagccacccaacagataccacatcgcagcacgtgcttacgcaagtg
ctcagggcgccccccttcgacaacaccaccaccaccgccatccttcaataccacaacttctcctcgccatcggcaaagcctgtggcc
gccctcatgcccgctttcaacgacacccccaccgtcaccgccttcaccaggagcttgagaagccccaggagggtggaagtgcc
caccaagatcgaccacaatctcttcttcacggtggggctgggcctcaacaactgcccgcgcggcgccagggcccgaaactgcc
agggccccaacggaacacgcttcgccgctagcatgaacaatgtgtcgtttgtgctgccctccaacttctcactgctgcaggcaca
tcagcaaggcttacgggggggggaacgagcatcttcacggcgagaatcatcccatccatcttcatggctacgatttctacatcgtc
gctgagggattcgggaatttcaatcccaccagagatacgcccaaattcaatctggtggacccacctatgcggaatacggctagt
gtacctgttggtggttgggccgtcataagatttgttgctgataatccaggagtctggttgatgcattgccacttggatgtccatatc
acttggggattagccatggccttcctcgtcgataatggcgtcggcgaattgcaaactttacagccgcctccaccagatcttcccctt
tgttga
>SmLAC18.SMil_00022417
atgcaacgcaaaattcattttcttgccaattttgttgctctaatcctctctctcactcttgcaaatgccgagactcatcaccatcaatttgt
cgttcagacaactccggtgaagcggctgtgcagaactcgcaacataatcactgtcaacggccagtttccggggcccactttgga
ggtcagagacggcgacgccctcgtcatcaaagtcatcaacgctgctcgctacaacgttaccattcactggcacggggttcggca
gatgcgaacgccttgggctgacgggcctgagtacgtgactcagtgcccgattcagcccggggccagctacacctaccgattca
ccatcgagaatcaggaaggaaccttgtggtggcatgctcacagcagatggcttagagctacagtctatggagccctcattattc
accccaaactcggtgttccctatcctttccccaagcctaacacacagattcctatacttcttggagaatggtgggatagagacatca
tcagtgtgcaacgacaggcgattttcacgggcgcggcacccaacgtctccgatgcatacaccatcaatggccagccgggaga
cctctacagatgctcaaccaaagaactcttcttcactgttgccaaccacatgctaaccgtggtcgggaacgacgccgcctacaac
cggcccttcgccaccagagtggtcatgatcggaccagggcagaccaccaatgttctgctcactgccgatcagccagccggccg
ttattacatggcggcacgtgcatacgccaccgctcaaaacgcagcgtttgacaacacaactaccactgcagtcttggagtatgac
tctgctcctcgcccttccacaactgggaagcaaagacctttgctgcctgcacttccaagattcaacgatacaaatacagcaactgc
atatactagccagctcagaagcctgccatccaagaacaccaaagtcccaactcaaatcgatgagaacctcttcttcacagtcgga
ttaggcctcgtcaactgcaccccccggcccccgatgccaggggccgaacaacacgcggttcgctgccagcatgaacaatatctc
gttcgtgcttccacgtagcacctcgctcttacaagcctactaccaaaatataccgggcgttttcaccacagattttccatcggttcctc
ctgttgcatttgactacacaggcaacgtgagccgggggctgtggcagccgcagcgcgggactaagctgtacaagctcaagta
tggctcaaatgtgcaaatagtgctgcaagacactgccattgtctcaaccgaggaccatccccctgcatctccatggctaccatttcta
tgttgttggccagggttttggcaacttcaaccccagtagagacactgcaagattcaaccttgttgatccacctattcgcaacaccat
caacgtgcccgttggaggctggaccgtgattcgttttgttgcagacaatccaggggtttggctgatgcactgtcacatagactcc
catctcagatggggcttggccatgtcattcttggtcgaaaatggaccggggaagatgcagtctgttgaggctcctccacctgatc
tccctccttgttag
>SmLAC19.SMil_00023003
atgggaactaccaagacgttgatcttgcaattctcagcagtttttctcttacatagtgaggtgatttatagctatgctatgactcatca
tcactataacttcgacgtggtggaaacaccatacaataggttgtgcagcaacaagactatactgactgtaaatgggcaatttccg
gggccaactatatatgtcactgagggagacaccattgtcgtagtagtcgccaacaaagcaacacaaaacataaccattcactgg
catggggtgaagcaaccaagatatccgtggtccgacggccctgaatacataactcaatgcccgattcgaccggggactaatttc

agtcagaagatcgaattctctgacgagatcgggactctttggtggcacgcacacagtgactggtcaagggctacggtgcatgg
agcagtgattatatatcccaagaagaaggatgattatccttatcatgtgccctacgcacaaatcccaattatattaggggagtggt
ggaaaagtgatgttcaagctgttcttagtgaatttcttgaaaatggaggagagcctaataattctgatgcttttctcataaatggtca
accaggttacttgtacccttgctctacacaagatacgttcaagctcaccgtcgaccacggcaagagatacctaattcgaatggtta
acgccgtgatgaacaccatcatgttcttcaaaatcggcggccacaacgtcaccatcgtcggctccgacggcgcgtacctgaagc
ccttcacctccgattacatcgccatctcccccggccagactatcgacttcctcctcctcgccgaccaaaaccctagccgctactacat
ggctgcccgtgcctacgccgtcgccggcaacttcgacaacaccaccaccaccgccctaatcgagtaccgctccgccgcccccg
acacccctccgccgctccccccctctcccggaatccaacgacaccctggcgtcggccgatttcaccgcgaaattcaaaatcctaacc
caaagaaaccgcccaatcaacgttcccttaaatccttcaaataaattattcttcaccctttcaatgaacaccataccatgttcaaatag
cacatgtttagggccatttggcgataggttttccgcaagcgtgaataaccaaacattcaatctgccaagaatctcaatcctcgaag
cttattacaaagggatcaacggcgtctacggtggggacttcccgggcaggccgccgctggccttcaactacacggaggcgatc
gtgccgagggcgctgtgggtcccggacaactcgacgaaggtgaggtacctcgattataattcgacggtggagatcgtttttca
ggggaccaacgcggtgcgaggcgtggatcatccgatgcatttgcacgggtatagcttctacgttgtgggtcgtggatttggga
atttcgatgtgaagagagagatcccaggaattataatcttgtcgatccgccattgatgaacaccattgcagttcctgtaaatggctgg
accactatcagattcagggccgataacccaggagtttggctgttgcattgccatttggagagacatataagctggggaatgatg
atggtgttcatcattaaaaatgggaagaccccagcagcgaaaatggaccctccacctccagatttcccaagatgctga
>SmLAC20.SMil_00023004
atgggatccacaaagacgacgagaatcttgcattttctagcatttttctcttagctggagtgatttctatccatgccaagacccatc
atcaaacctttgtagtgagtgatgctccatatagtaggctgtgtagcaacaagagcatattgactgtaaatggaaagtttcctgga
ccgactgtacgtgtaactgagggtgataccattgcaattgttgttgtgaatagagcaagagaaaatataaccattcattggcatg
gagtgaagcagccgagatatccgtggtcggacgggccggaatatataacgcagtgcccgattcagcccggatctaattttagt
cagaagattgtgctgtcggatgagatcgggactatgtggtggcatgcgcatagcgactggtcgcgtgctacggtgcatggtgc
gctcatagtgtatcctcttatccagaatgattatcctttccctgtgcctgatgaagaagttcccatcatattaggggagtggtggaa
aagtgatattcaggctgttctcactgaatttcttcaaactggaggagaccctaataattctgatgcatttcttataaatggacaaccg
ggtgatttgtatgaccttccatgctcaaccaaagacacgttcaagctgagtgtggaccacgggaagagatacctaattcgaatg
gttaacgccgtgatgaacaccatcatgttcttcaaaatcggcggccacaacgtcaccatcgtcggctccgacggcgcgtacctg
aagcccttcacctccgactacatcgccatctccccgggccaaaccatcgacttcctcctcctcgccgaccaaaaccctagccactac
tacatggcctctagagcctacgccgtcgccggagatttcgacaacaccaccaccagcgcccgcgtggagtactccggaaacta
caccccgccggcgtccccactgctcccaaccctccccgatttcaccgacacggccgcatcagtgaacttcaccgcccgactccgc
agcctcgcctacaaaaaccacccaatccaagtgccgttgaacgtgacgacgaatctcctcttcaccctctccatcaacaccagaaa
ctgccccaacgcggactgcttggggccgcagggcaaccgcctcctcgcgagcgtcaacaacatcacgtttcagtcgccgcgg
attgcgatcctgcaggcctattacgagcggatcagcggcgtctacagcgcgaatttcccgagcaacccccgtttccgttcaact
acacctcggactccgtcccgcgggacctgtgggagccgcggaacgggacgagggtgagggtgctcgactataactccacg
gtggagctcgtctttcaaggaactagtacggtaaacgcgccgattgatcatcccatgcatctgcatggccacagcttctatgttgt
aggatggggatttgggaatttcaacagcactcgggacccaccgaactacaatctcgtcgacccgccgcttcagaacaccatcgc
ggtgcctagagccggttggaccgcgattagattccaagcaaataatccaggggtttggctaatgcattgccatttcgagagaca
tataagctggggaatggagatggtgtttattactagaaatgggaaaggcgcgaacgaaacgatgcttcctccaccgtcggattt
ccctatgtgctga
>SmLAC21.SMil_00023210
atgcacaccactgaaaaaatgttttgttataggaagctcaaggttttcatgctgtgctaccttggtattattatggctccagtccatgc
tgtagttcatcataagtttgaagtgagaagaacttcacacacaaggctatgcaccaccaaaagcatgctaacggtaaacgggca
gttcccagggccaactatatatgctcgaaggggagaattggtggtagtcgatgttattaatcgtgccgatgcaaatattactatcc
attggcatggagtaaaaatgccgagatatccatggacagatggcgccgacgctgtgacgcagtgtcctattactcccggcaaa
agctttcgacaacgagttctgctctccgacgaagaaggcactttgttttggcacgcccacatcgattgggctcgagctactgtgta

tggagccatcgtcattctaccgcccaagacacacacttatcctttccctaagcctcatgctgaatttcccatcttactaggagagtgg
tggaatgatgatgtgcaacaagtttacgaggattttatggcctcaggagccgctccccaattgtctgatgctttcctcgtgaatggt
caaccaggcgacctctatccctgctcaaaacaagatacattgaagttgagtgtggaaccgggcaagacttacctgatcagaatg
gttaacgcaatgatgaacttcattacgttcttcaaaatcaaggaccacaacgtgacggtggtgggcatggacggcgcctacacg
aagccgctgaacaccgatcacatcgcgatcatccccggccagaccatagatttccttctggaagccaaccagccgcccagccgt
tattacatggccgccagagtatacgccggcgccccccttggggcgtacttacctcccaaccacagtaatcgtggagtacgtcggt
aactacacccggccctcatcactgctgcttccgtctttccctgaattcgacgatttccgtgaggccagtccacccatttcagcaaca
ggctcagaagccttgccgacgacaactaccccatcaaagctgctgggcagcatgaacaacgtgaccatgttgttgcccaaaac
ccaaaatattcttgaggcctattacaaagggatcagaggagttttcacgcccgatttcccagcgatgacaccaataatatacaact
tcacgcaaggttataagccggagaatttggcgcccgcccatttcggaacggcggtgtacatgttggactacaactcggaagtc
gagatggtgcttcaaggcaccgactatgggtatggcgatggaatcgatcatcccatgcatttacatggacacagcttctacgttg
tcggaactggatacgggaacttcgacccgaacacggatccgccaaactttaatctcattgacccgccgttggggaacatggttg
ccgttccaagaaatggatggagtgctatcagatttaaggctaacaatccaggagtgtggttcatgcactgtcatttcgagcgtcat
cacagttggggaatgatgatggcgttcatcgttaaggacggggaagcccccaatgagaagatgttgcctccgcccccagatat
gccccgctgtgaagcgccccctgcagcctcaacaatttag
>SmLAC22.SMil_00023712
atgttgcctgttgcttcgttgccagcagccacgacagccctcacgttaggcattcttctttctataatgctgcctcagcccgcatcctg
catcaccagacactacacatttaatattgtgcaacacaacgtgacacgactgtgcaaaacgaagagcattattagcgttaacggg
aagtttccgggacctagactcatcgcaagagaaggcgatcgtgtgttagttaaggtggtcaacaacgtgactaacaatgtatcc
attcactggcatggaatacggcaacttaatagcggatgggctgatgggccggcttatgtaacacaatgcccgatacaaacggg
ccagagctacacgtacaacttcaccatcacagggcagagaggtactctgttctggcatgctcacatctcatggcttagagcaact
ctttacggaccaataatcatcctcccaagacgcaacgacacctatcccttcaagaaaccatacaaggaagtccccatcatatttgg
ggagtggtggaatgcggatcctgaggcagttattaatcagtctcttcagacggggggcggtccaaacgtttccgatgcatacac
cattaatggccttcctggacccttgtacaattgttcctctaatgatacattcaggctgagagtaaagccagggaagacgtacctgc
ttcgtctgataaatgctgccatgaacgatgaactatttttcagcattgccaaccataccgtcaccatagttgaagccgatgcagtgt
atgtcaaaccatttgctactagcgtggtggtcattacccctggccaaacaacgaatgttctgctcaagacaaagcctcaccaccca
aacgctacattcctcatggctgcaaggccgtatttcacaggccagggcacttttgacaattcaacagttgctgctttacttgaatat
gaagaccggccagatcactcgtttaacaagataaagacacttttcaagcctactctaccaccaataggtgccactagctttgttgc
caatttcaccaagaagttccggagcttaaataacccaaaatatacgatagcaactgtcccactgaaagtagacaagcatttttttctt
cactgtgggacttgggtcaagcccatgccctcaaaatgcaacgtgtcaaggacccaacgggacaaaattttcagctgcagtcaa
taatatttcttttgttcttcctacaacagctatgctacaagcccacttcttcgaaaactcttcaggagtctacaccacagatttcccaac
caatccaccgaatccattcaattacacgggaactccaccgaacaacactgtagtgagcaatggaactcgggttgtggctcttcctt
tcaatgctagtgtggagctagttcttcaagatacgagcattcttggagctgagagtcatcctcttcacctacatggtttcaatttcttt
atagtcggtgaaggattcgggaattatgatccaactaatgaccctgcaaagtttaacctggtggatcctgtcgaaaggaataca
gtaggtgttccatctggtggctgggtagcccttcgattcctagcagacaatccaggagtatggtttatgcactgccactttgatgta
cacacaagttgggggttgagaatggcgtggattgtacgggacgggccgctagcaagtcaaaaactaccaccgatcccatctg
atcttcccaagtgttga
>SmLAC23.SMil_00023714
atgttgttcagtgcagctttagttcatcataagtttgaagtgagaagatcttcacacaccaggctatgcaccaccaaaagcatccta
acgataaacgggcagttcccagggccaactatatatgctagaaggggggaattggtggtaatcgatgttattaatcgcgccga
cgcaaatataagtatccattggcatggagtaaaaatgccgagatatccatggacagatggcgcgagccatgtgacgcagtgtc
ctattagtcccggcaagaggtttagacaacggatgttgctctccaacgaagaaggcactttattttggcacgcccacagcgattg
gtctcgagctactgtgtatggcgccatcatcattctaccgcctaagacacacacttatcctttccctaagcctcatgctcaagtgccc
atcttactaggagagtggtggaatgctgatgtgcaacaagtttacgaggatgcaatagcgcaaggaatcgatgccaatttttctg

atgctttcctcatgaatggtcaacctggcgacatgtacccctgctcaaaacaagatacattcaagttgagtgtggagccgggcaa
gacttacctgatcagaatgatcaacgcaatgttgagctacattatgtacttgaaaatcaaggaccacaacgtgacggtggtggg
catggacggcgcctacacgaagccgctgaacaccgatcacattgcgattgcccccggccagaccatagatttccttttggaagc
caaccagccgcccagccgttattacatggccaccaaagtatacgccagcccctggtggcatacttacgtccccaccaccggaatc
ctcgagtacgttggtaactacacgcccccctcatcactggagtttccatattaccctaaattcggtggctatggctgggccgagtcc
atccatttcagcagcaagctcagaagcctcgccaacgacaagcaccccatcaaagttcctatgaacatcacccacaacctcttctt
cactctcaccatcaatatgtggccctgtgagacaaattcttgcgagaggaacaacagggtgctggccagcatgaacaacatctc
catgctgtccccgcaaaaccaaaatattcttgaggcctattacaaagggatcggaggagttttcacgaccgatttcccggcgaag
ccagcaagaatattcaacttcacggaaggtcataagtcattgtatgaggggcgcaccgaattcggaacggctgtgtatatgttg
gactataactcagaagtggagatcgtgtttcaaggcaccaactttggcgagggaagcgatcatcccatgcatttacatggatac
agcttctacgttgtcggagctggatacggggacttcgacccaaacagggatccgcaaaactttaatctcatcgacccgctcttgat
gaacactgttaccgttccaagaaatggatggagcgctatcagatttaaggctaacaatccaggagtgtggtacatgcactgcca
tttcgatcgtcatcagacttggggaatgaagatggtgttcatcgttaaggatggggaagcccccaatgagaagatgttgcctcc
gccccctgatatgccccgctgtcaaccgccccctcagaaattattatttaggatttaa
>SmLAC24.SMiI_00023969
atgggtttgcaaatttctatagcattatttgcagcattcttggcaatgctccaccttcctctccctgctgaagccattacgaggcacta
caacttcaacatcatgatgcaaaacgtgacgcgattatgccacacgaggagcatagtcacggtgaacgggaaattccccgggc
ctcgggtggtggctagggagggcgaccgcctcgttatcaatgtgaccaaccttgttcctaacaatatcaccatccattggcatgg
gattaggcaacttcgaagcggttgggccgatgggccggcgtatataacccaatgtcccattcaaaccgggcaaagctatgtcta
caatttcacaatagtcggacaaaggggcactctttggtggcacgctcatgcctcttggcttagatccaccgtctatggccctattat
catccttcctaaaaataatgtggcctatcctttccccaagccatacaaacaaattcccatcatctttggagaatggttcaataccgata
ccgaagctataattagccaagctcttcaaaccggcggcggccccaacgtctccgacgcctacaccatcaacggacttcccggcc
ccttgtacaactgctccaccaaagatacattcaggctgaaggtaaaggcgggaaaaacctacctcctccgcgtaatcaacgctg
cgctcaacgacgagctcttcttcagcatcgccaaccacacgctcaccgtcgccgatgtcgacgctgtctacgtcaaacccttcgaa
accgacaccattctcatcgctcccggccagaccaccaaccttctcctccgcaccaaagccgccgctcccaccgctgcattcctcat
gaccgcgaggccctacgtcaccggctccggcacctttgacaactccaccgtcgccggaatccttgaatacgaatccaccccttcc
acgaagcacctcccccttcttcaaaccaaccctgccgccgctcaacgacaccgccttcgccaccaacttctccagccgcctccgca
gcctcgccaccccacagttcccggcgaacgtgccgcagagcgtggacagacgcttcctcttcacggtgggattaggcacgaa
gccgtccgacgacggcgctgctgcaggcccacttcacggggcggtcgggcggcgtgtacgcggcggacttcccctacagcc
cgctgcggtggttcaactacaccggcagcccgcccaacaacacgatggtcgggaatggaacgaaattgatggtgttgcgttca
acacgagcgtggaggtggtgatgcaggataccagcattttaggggcggagagccacccccctccacctccatggatacaatttc
ttcatcgtcggacaaggcttcggcaactacgaccccattaacgaccccaagcggttcaatctggtcgacccggtcgaacggaac
acggtcggtgtgccgcccggtggatgggttgctattcgcttcttggccgataatccaggtgtgtggtttatgcattgtcacttgga
agttcacacgagctggggattgaagatggcatggcttgttttggacggaaagcttcccaatcaaaagctgcttcctccgccttcc
gatcttcctaaatgttga
>SmLAC25.SMiI_00024180
atgcgtcgttccttggtatttctagcatgggccttggctcttttagcttgctcttcgtcatttgcctcagctcggatcgtgcgacatacg
tttaatgtgggaaatctgactgtgaatcggttatgtcgtgatcaagtaatcactgcggtgaatggcggtcttcccggaccagccat
tgtagctcgagatggtgatacgcttgttgttcgtgttaacaacatatcaccatataatgtgactattcattggcatggggtgttccag
ttaatgagcgcatgggccgatgggcctgaatatatcacccagtgtccgattcgacccggtcaaagctacacttatagattcaacg
tgagccgccaagaaggaactttgttgtggcacgcacacttcaaagcgctccgagccacgatccatggagccttaataatccgac
ccgcacgaggtcgcacgtacccatttccagcaccattccgtgaaattccaatcgtcctcggcgagtggtggaatgccgatatcat
ggacatcgaggaggaagccgtctctcttggccgaccgcctaacgtctccaatgcttttactattaacggccagcccggagatctc
ttcccatgctcctcaaacaacacggttaggttcacgttagtacaaggaaggacctatcttctacggataatcaacgctgcactcaa

cactccattattcttcaagatcgccaatcacaaattcacagtggtagccatcgacgcgtcctacaccgacccctacgacagcgacg
ttttggttttatcaccgggacagaccattgatgccctgatgaccgccgatcaagcgccggcccgctactacatggccgcgagcg
cctacgtcgtccctccacttgctccctacgtcaacatctccaccaccgcaatcgtgtcctacattggagcagcggcgtcgactgcg
gcgcccctaatgcccgttatgcccgccgccaacgactccgacacggggcacaggtttttaagcaacttgaccggtttgaccagt
agcccgcattggaccccggtcccactggagatagacgagcgcatgttcgtggtggtggggctaggcctcaccccatgcgaga
acccgagcggggtttgtggtgggcccaacgggttgaatatagcggcgtccatgaacaatgtgtcgttcgagctccccacagg
gctatcgatcatggaagccttggtgaacaacgtgagcgggatatacacgaccgatttccccgacaacccaccggtgacgttcg
actacacgagcccgagcaatgcacagaacccggagtttctggggacggtgaaggggacgagggtgaagcagttcaagtac
aatgcaacagtggagatggtgttccagaacacggcggtgctgtcgtcagatgatcacccaatgcacttgcacggcctcaatttct
acgtggtggcgcaaggctttgggaactacaatccccaaactgatggggcgaatttcaacttggtgaatccacaagagcggaat
actgtggtggttcctgccttcggatgggtggtgattaggttccgagctaacaatccgggtgtatggttcgtgcattgtcacataga
cggacacgtgccgtggggtatggctaatgcttttatcattgaaaatgggcccacaccggatacaactttgcctccacctcctgcg
gatcttccacaatgctag
>SmLAC26.SMil_00025257
atgttgtcgactgcaaagcttttcttcctctggttcttaggtatcattctgctcggaggcgtaacgccgagccatgccgcaactcgtc
gctatcggtttgtgctggggaattctaatcactctaggatgtgtaccaacaagacaatgctgacgataaatgggcagttcccggg
cccaaccatatatgctagaagaggagatctcgtcatagtcgatgttatcaacagtgcagaccataatataaccatccactggcat
ggagtgaaaatgccgcgatatccatgggcagacggccccgagttcgtgacacagtgccccatcagccccggcacgagcttca
ggcaacgggtcgtcctctccgacgaggaaggcactctgtggtggcacgctcacagcgagtggtatcggaattctgtctacggc
gccttcattattctgccgcgcaggacagagacatatcctttcgctaagcctcatgctgaagttcccatcttactaggagactggtttc
atggcgacgtgcaggaaatcatggagacattcctcgccaccggcggcgatccagaagtttccgatggtttcctcatcaacggcc
aacctggcgacttgtatccatgctcaagccaagacacattcaagctgagggtggagccgggaaaaacctatatgatcagacta
gtaaacgcggtgatgaacaacatcatgttcttcaagatcgccaaccacaatgtgaccgtggtggccaccgacgccgcctacacg
aaacccctgcgctccgactacatcgccatctccccggggcagaccatcgacctcctcttcgtggccaaccagccccccagccgct
actacatggccgccagagtctactccagcggcggcgacttcgataacaccaccaccaccggcatcatcgagtacgtgggaaac
tacacccctccggccacgccggccctcccgtccttcccgccgttcaacagcacggccgcgtcccacaacttcacctcccaactaa
gaagcctggccaacgcaaaataccccatcgacgtccccagaaatatgtcggacatcctgttcttcaccctctccatcaatctccgg
ccctgccccaacgattcctgcgcggggcccttcgcggagcggctgatggcgagcatcaacaacataacgctgcagctgccca
agaccgacttccttcaggcctactaccggcgactcagcggcgtctacacgacggatttccccgataacccgggcttcgtcttcaa
ctacacgcaaacaacaatacccagggatctggcaaggccgcagaacgggacggcggtcaacattctcgacttcaatgccacg
gtggagctcgtcttccaagggaccaacctcgccggagggattgaccaccccatgcatttgcacggctacagcttctacgtcgtc
ggatccggcttcgggaacttcaaccggacccgggacccgcccaattataacctcgtcgacccgccgttgatggagaccatcgc
cgtgccggaaaatggatggacggcgattaggtttaaggctaacaatccaggagtgtggttcatgcactgccatttcgagcgtca
cgtgagctggggaatggggatggtgtttatcgttagggacgggccaggcgccaacgagaagatgctgccgccgccaccgg
atatgccacgctgttaa
>SmLAC27.SMil_00026282
atggattcttggatgattagggctttcatcctcgctgcttgcttgcttcctatgatggtcgaatctagagttcgccattacaagttcaa
cgtggtaatgaaaaatactagtcgcttgtgttcgacgaaaccaattgttactataaatggaaactttccaggaccaaccatttatgc
gcgagaagacgacacagtgctagttaaggtggtcaaccatgtcaactacaatgtctccatccattggcacggaatcaggcagct
tcggacagggtgggccgatgggcctgcatatatcacccagtgtccgattcagactagccaaagctttgtctacaacttcaccatc
acgggccagagaggcacgctgctttggcacgctcacatcctgtggctcagggccaccgtccacggcgccctcgtcatcttgcct
aagcttgggattccttaccctttccccaaacctgaccacgaacgagtcgtcatcctcgctgaatggtggaaatcggacactgaag
atgtgatcaatgaggccatgaattccgggctcgcccccaacgtctccgatgcccacaccatcaacggctatcccgggccttcccc
taattgttcctccatagatgggtaccaactagatgttagagcgggcaaatcatacctcctccgcctaatcaacgctgcgctgaacg

aagagctcttctacaagatcgccggccacaatttcaccgtcgtggaagtcgacgccacttacgtgaaacccttccagacggaca
ccgtggtcatcgccccgggccaaaccaccaacgtcatcatcacggccaacaagcgcggcggcaagttcgccatgacggcctc
caccttcatggacacccccgtggtggccatcgacaacgtaaccgccatggccacgctccactactccggcacgctctcggccag
ccccaccactctgaccgccaccccggcccgcaacgccacccccgtggccgacaccttcgccacctccctccgcagcctcaactcc
aaaacctaccccgcaaacgtcccgcaaagggtggaccgctcgctcttcttcgcagtcgggctcggcgtcaacgcctgcccgac
ctgcaaagccgccaacggaagccgagtggtggcgagcgtcaacaacatcacgtttgtgatgccgaccaccgctttgctccagg
cacatttcttcaacatcacgggagtctacaccaccgacttccccgggaacccgcccacgcccttcaactacaccggcgccccgcc
gcagggcttagccaccaccaccgccaccaaggtttaccggctgccgtacaacgccacggttcaggtggttttgcaggacaccg
ggatcatcgcggcggaaaaccaccccgttcatctccatggattcaatttcttcgtggtgggcaaaggattggggaatttcaactc
gaaaacggatccgaaaaagttcaacctggtggatccggtggagaggaacacggtggcggtgccgtcgggtggatgggtgg
ccatcagattcacggccgacaatccaggagtgtggttcatgcattgccacttggaagtgcacaccacgtgggggctcaaaatg
gccttccaagtcgataacggaaagggcccaaatcagtcgctcttgccgccgcctcaagatttacccacatgctcataa
>SmLAC28.SMiI_00028093
atgttttcttataggaagcttaaggttttcatcctatgcttcttaggtattattatgctcggaggcatgggtccagtccatgctttagttc
ataagtttgaggtgagaagatcttcacacaccaggctatgcagcaccaaaagcatgttaacagtaaacgggcagttcccaggg
ccaactatatatgctagaaggggagaattggtggtaatcgatgttatcaatcgcgccgatgcaaatataagtatccattggcatg
gagtaaaaatgccgagatatccatggacagacggcgtcacccaagtgacgcagtgtcctattagtcccggcaagaggtttaga
caacggatgttgctctccaacgaagaaggcactttattttggcacgcccacatcgattggactcgagctactgtgtatggcgccat
catcattctaccgcccaaggcacacacttatcctttccctaagcctcatgctcaacttcccatcttactaggagagtggtggaatgct
gatgtgcaacaagttttcgagaagtctatagccgaaggagtagctgccgatttttctgatgctttcctcatgaatggtcaacctgg
cgacctgtatccctgctcaaaacaagatacattgaagttgagtgtggagccgggcaagacttacctgatcagaatggttaacgc
aatgatgaactacattatgtacttcaaaatcaaggaccacaacgtgacggtggtgggcatggacggcgcctacacgaagccgc
tgaacaccgatcacattgcgattgcccccggccagaccatggatttcctgctggaagccaaccagccgcccagccgttattacat
ggccggcagagtatgggccagcccctattggcatacttacatccccaccacaggaatcgtcgagtacgttggtaactacacccc
cccctcgacaccggtgcttccctctttccctggattccaaggctgggcggagtccacccatttcagcaagaggctcagaagcctg
gccaacgacaactatcccatcaaacttcctatgaacatcacacacaacctcttcttcactctcaccattaatgtggggccctgtttctc
aaattcttgcaagaatgacggcaggctgctggccagcatcaacaacatctccatgctctacccggaaacccaaactattcttgag
gcctattacaaagggatcggaggagttttcacgcccgatttcccggccaagccatcaacaatattcgacttcacgcaaggttata
agcccgaggatgaggggcacacccaattcggaacggctgtgtatatgttggactataactcggaagttgagatcgtgcttcaa
ggcaccaacttcggcatcggaatcgatcatcccatacatttacatggacacaacttctacgttatcggaactggattcgggaactt
cgacccaaccaccgatccgccacgctataatctcattgacccgccgttgatggacacggtttccgttccaaaaaatggatggagc
actatcagatttaaggctaacaatccaggagtgtggtacatgcactgccatttcgaacgtcattacacttggggaatgaagatgg
tgttcatcgttaaggacggagaagcccccaatgagaagatgttgcctccgcccccagatatgccccgctgtgaagcaccatctc
aacaattattgtttaggatttaa
>SmLAC29.SMiI_00028534
atggagcgctctttgatgttttcagcatgggttttggctcttttggtttgtttgtcgtcatttgcttccgctgctatcgtggagcacacat
ttcacgtgaaaaatcttacagtgaacaaattatgcagaaatcaagttataacggcagtaaatggtagtctcccagggcccactctt
cgagtgcacgagggtgataccttagtcgttcatgtgtttaacaagtcgccgtataacctaactattcattggcacgggattttccaa
atccttagcgggtgggctgacgggccggagttcgctacccaatgcccgatccgacccggtcagagctacacctacagatttaac
ataacaggtcaagagggaactctttggtggcatgcacacgtcggatggctccgagccacagtttacggagccttggtaatccg
acccagatccggtcactcattcccatttccaaagccacacagagaaatccccattgtccttggggagtggtggaatgctaatgtta
ttgacgtagaaaaccaagccttagccacgggtgccgcacctaacttatccgatgcttacactattaatggccatcccggaaatctc
tacccgtgctcatcaaacgatacgttcagactaacagtggtgcacggaaagacatatctcctacgcataatcaacgctgcactca
acaatcaactttttttcaaaatagcgaatcataacctcaccgtagttgcggtggacgcctcctacacgaacccctacaccaccgac

gtggtgttggtggcgccggggcagaccaccgacgtcctcctcactgccgaccaaacgccggcgagatactacatggcggcg
agcgcctaccaaagtgccgccggcgtcccgttcgacaacaccacgaccaccgggatcgtggcctacagcggaacgacgccg
tccgcgccgatcatgcccctcctgccggccttcaacgacacgccgacggcccacagattcttcagcaacctaaccgcattagtctc
gagccgattttgggcccccgttccccgccacgtggatgagcgcatgttcgttacaatcgggctgggcctgtcggcctgcgacct
cccccagtgtcagggcccgttcgggctgaagttggccgccagcatgaacaacgcgtctttccagttcccgacgaggctgtccat
gctggaggccttctttagaggcgtcggcggtatttacaccgccgactttcccgataacccgccggtgcgattcgactacaccaac
tctagcaatagcctcaaccaggcgctgctgaatacgacgaaatcgaccaaggtgaagaaggtcaggttcaattcgacggtgg
aggtggtgctgcagaacacggcgttcctcggagtggaaaatcaccccattcatttgcacggattcaatttctatgttttggctcaa
ggttttggcaattataaccctgcacttgatactaggaagttcaattttgtgaatccgcaggagaggaacaccatcggcgttccggt
cggtggatgggccgtgataaggtttcgcgccaataatccaggtgtgtggttgattcactgtcacctggatgtgcacttgccgtgg
ggtctagcaacggctttcgtggtggagaacgggcctacgccagcgacaacgctacctccgccgccggctgattttcccaagtg
ctaa
